# Supplementary material for: Carbon peaking prediction scenarios based on different neural network models: A case study of Guizhou Province
Source: PLoS One. 2024 Jun 25;19(6):e0296596. doi: 10.1371/journal.pone.0296596 (PMC11198898; doi:10.1371/journal.pone.0296596)
Supplement: S1 File — (DOC) [file pone.0296596.s001.doc]

Current location:[Home page](http://hgk.guizhou.gov.cn/index.vhtml) > [Statistical publications](http://hgk.guizhou.gov.cn/publish/channels/c6/c6_1.html)> [Statistical Bulletin](http://hgk.guizhou.gov.cn/publish/channels/c7/c7_1.html)> [Statistical Communique on the National Economic and Social Development of Guizhou Province in 2022](http://hgk.guizhou.gov.cn/publish/articles/c7/2023/05/a1016/a1016.html)

Statistical Bulletin

Statistical Communique on the National Economic and Social Development of Guizhou Province in 2022

Date: 2023-05-17 Article Source: Provincial Bureau of Statistics No.: [[Big](javascript:doZoom(20)) [Medium](javascript:doZoom(16)) [Small](javascript:doZoom(12)) ]

Guizhou Provincial Bureau of Statistics National Bureau of Statistics Guizhou Investigation Team

(17 May 2023)

2022 is a very unusual year in the history of Guizhou's development. The whole province has thoroughly implemented the spirit of the 20th National Congress of the CPC and the spirit of General Secretary 's important speech on inspecting Guizhou, comprehensively implemented the important requirements of "epidemic prevention, economic stability and development security", adhered to the overall situation of high-quality development, coordinated epidemic prevention and control and economic and social development, coordinated development and security, and made every effort to focus on the "four new". Efforts should be made to build "four districts and one highland", and every effort should be made to solve multiple constraints and difficult problems, so as to make new achievements in promoting high-quality development.

First, comprehensive

According to the unified accounting results of GDP, the province's GDP in 2016 was 4.58 billion yuan, an increase of 1.2% over the previous year. Among them, the added value of the primary industry was 286.118 billion yuan, an increase of 3.6%; the added value of the secondary industry was 711.303 billion yuan, an increase of 0.5%; the added value of the tertiary industry was 10190 1.37 billion yuan, an increase of 1.0%. The added value of the primary industry accounted for 14.2% of the GDP of the region, an increase of 0.2 percentage points over the previous year; the added value of the secondary industry accounted for 35.3% of the GDP of the region, an increase of 0.1 percentage points over the previous year; The added value of the tertiary industry accounted for 50.5% of the GDP, down 0.3 percentage points from the previous year. Per capita GDP was 52321 yuan, an increase of 1.2% over the previous year. The total labor productivity was yuan 103797 per person, an increase of 1.6% over the previous year.


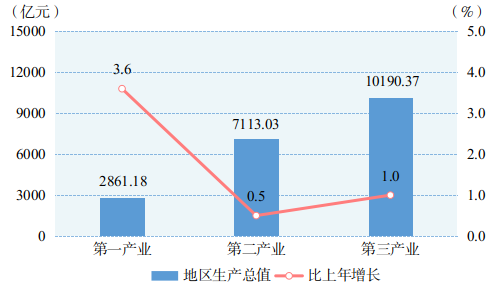


**Figure 1 The added value of the three industries and their growth rate**

At the end of the year, the permanent population of the province was 38.56 million, an increase of 40000 over the end of the previous year. Among them, the urban permanent population was 21.14 million, accounting for 54.81% of the permanent population at the end of the year, an increase of 0.48 percentage points over the end of last year. There were 425,000 births in the whole year, with a birth rate of 11.03 per thousand, and 282,000 deaths, with a death rate of 7.32 per thousand. The natural growth rate was 3.71 per thousand.

**Table 1 Number and Composition of Resident Population at the End of 2022**

| **Indicator name** | **Absolute number (10,000 persons)** | **As a percentage of the resident population at the end of the year**  **Specific gravity (%)** |
| --- | --- | --- |
| **Resident population at the end of the year** | 3856 | 100 |
| **By urban and rural areas** |  |  |
| **Towns** | 2114 | 54.81 |
| **Rural** | 1742 | 45.19 |
| **By gender** |  |  |
| **Male** | 1971 | 51.12 |
| **Female** | 1885 | 48.88 |
| **By age** |  |  |
| **0-15 years old (including less than 16 years old)** | 947 | 24.56 |
| **16-59 years old (including less than 60 years old)** | 2307 | 59.83 |
| **60 years old and above** | 602 | 15.61 |
| **# 65 years and above** | 468 | 12.14 |

At the end of the year, 18.78 million people were employed in the province, of which 9.81 million were employed in cities and towns, accounting for 52.2% of the province's total employment. In the whole year, 609200 new jobs were created in cities and towns, down 5.9% from the previous year. Among them, 151 thousand and 600 unemployed people were re-employed, an increase of 4.8%; 74 thousand and 900 people with employment difficulties were employed, down by 3.0%.

The consumer price of the whole province rose by 1.6% over the previous year. Producer prices rose by 5.7%. The purchasing price of industrial producers rose by 11.2%.

**Table 2 Increase and Decrease of Consumer Price in 2022 over the Previous Year**

| **Indicator name** | **Increase or decrease over the previous year (%)** |
| --- | --- |
| **Consumer prices** | 1.6 |
| **# Food, tobacco and alcohol** | 1.0 |
| **Clothes** | 0.6 |
| **Live** | 0.5 |
| **Daily necessities and services** | 0.8 |
| **Traffic and communication** | 5.4 |
| **Education, culture and entertainment** | 1.5 |
| **Health care** | 0.3 |
| **Other supplies and services** | 1.4 |
|  |  |

At the end of the year, the total number of market entities in the province was 4 million 368 thousand and 700, an increase of 12.4% over the end of last year. The registered capital of market entities was 12.75 trillion yuan, an increase of 36.4%. In the whole year, 745400 new market entities were established, an increase of 6.3% over the previous year.

II. Agriculture

The total output value of agriculture, forestry, animal husbandry and fishery in the whole year was 490.867 billion yuan, an increase of 4.2% over the previous year. Among them, the total output value of planting industry was 331.370 billion yuan, an increase of 3.5%; the total output value of forestry was 34.00 billion yuan, an increase of 3.9%; the total output value of animal husbandry was 94.140 billion yuan, an increase of 5.7%; The total output value of fishery was 7.960 billion yuan, an increase of 6.5%.

**Table 3 Gross Output Value and Growth Rate of Agriculture, Forestry, Animal Husbandry and Fishery in 2022**

| **Indicator name** | **Absolute number (100 million yuan)** | **Year-on-year growth (%)** |
| --- | --- | --- |
| **Gross output value of agriculture, forestry, animal husbandry and fishery** | 4908.67 | 4.2 |
| **Planting** | 3313.70 | 3.5 |
| **Forestry** | 340.00 | 3.9 |
| **Animal husbandry** | 941.40 | 5.7 |
| **Fishery** | 79.60 | 6.5 |
| **Agriculture, Forestry, Animal Husbandry and Fishery Professional and Auxiliary Activities** | 233.97 | 6.3 |

The sown area of grain was 41,830,500 mu, an increase of 0.04% over the previous year; the grain output was 11,146,400 tons, an increase of 1.8% over the previous year. The planting area of vegetables in the whole year was 21.88 million mu, down 3.7% from the previous year, and the output of vegetables was 32.7597 million tons, up 2.3% from the previous year. The area of edible fungi harvested in the whole year was 363,300 mu, an increase of 4.2% over the previous year; the output of edible fungi was 797,600 tons, an increase of 1.6% over the previous year. The annual tea picking area was 5.4148 million mu, an increase of 1.7% over the previous year; the tea output was 266,200 tons, an increase of 8.3% over the previous year. The annual garden fruit picking area was 7 million 427 thousand and 200 mu, an increase of 1.5% over the previous year. The annual output of garden fruits was 6.2937 million tons, an increase of 7.9% over the previous year.

At the end of the year, there were 15.4267 million pigs, an increase of 0.8% over the end of the previous year; there were 4.9224 million cattle, an increase of 2.7%; there were 3.5915 million sheep, a decrease of 7.1%; the 12246 of poultry was 0.12 million, an increase of 1.9%. In the whole year, 19.8473 million pigs were slaughtered, an increase of 7.3% over the previous year; 1.7391 million cattle were slaughtered, a decrease of 3.4%; 2.6744 million sheep were slaughtered, a decrease of 4.5%; the 18422 of poultry was 0.68 million, an increase of 4.2%. The output of pork, beef, mutton and poultry was 2,390,500 tons, up by 5.9% over the previous year, and the output of eggs was 336,000 tons, up by 21.2%. Milk output was 37 thousand and 300 tons, down 24.1%.

The annual output of aquatic products in the whole province was 268 thousand and 400 tons, an increase of 2.4% over the previous year. Among them, the output of aquaculture products was 264 thousand and 100 tons, an increase of 2.7%.

**Table 4 Output and Growth Rate of Main Agricultural Products in 2022**

| **Indicator name** | **Absolute number (10,000 tons)** | **Year-on-year growth (%)** |
| --- | --- | --- |
| **Grain production** | 1114.64 | 1.8 |
| **# Summer grain** | 257.61 | 0.7 |
| **Vegetables and edible fungi** | 3355.73 | 2.3 |
| **Pork, beef, mutton and poultry meat** | 239.05 | 5.9 |

III. Industry and Construction

In the whole year, the total industrial added value of the province was 549.313 billion yuan, an increase of 0.7% over the previous year. The added value of industries above scale decreased by 0.5% over the previous year. Among the industries above the scale, the added value of state-owned holding enterprises increased by 17.8%, joint-stock enterprises decreased by 0.3%, foreign and Hong Kong, Macao and Taiwan-invested enterprises increased by 3.8%, and private enterprises decreased by 28.8%. In terms of categories, the mining industry decreased by 18.4%, the manufacturing industry increased by 4.1%, and the production and supply of electricity, heat, gas and water increased by 2.6%.

Of the 19 key industrial sectors monitored in the province, the added value of 6 industries has maintained growth. Among them, the added value of computer, communication and other electronic equipment manufacturing industry increased by 45.9% over the previous year, that of wine, beverage and refined tea manufacturing industry by 32.6%, that of electrical machinery and equipment manufacturing industry by 31.2%, that of railway, ship, aerospace and other transport equipment manufacturing industry by 12.8%, and that of tobacco products by 6.7%.

**Table 5 Growth rate of added value of major industries above designated size in 2022**

| **Indicator name** | **Year-on-year growth (%)** |
| --- | --- |
| **Industrial added value above designated size** | -0.5 |
| **# Coal mining and washing industry** | -17.6 |
| **Non-metallic mining and dressing industry** | -27.8 |
| **Agricultural and sideline food processing industry** | -25.3 |
| **Wine, beverage and refined tea manufacturing** | 32.6 |
| **Tobacco products industry** | 6.7 |
| **Chemical raw materials and chemical products manufacturing industry** | -14.4 |
| **Pharmaceutical manufacturing** | -12.6 |
| **Non-metallic mineral products industry** | -40.2 |
| **Ferrous metal smelting and calendering industry** | -26.0 |
| **Non-ferrous metal smelting and calendering industry** | -9.6 |
| **Automobile manufacturing** | -8.3 |
| **Electrical machinery and equipment manufacturing** | 31.2 |
| **Computer, communication and other electronic equipment manufacturing** | 45.9 |
| **Production and supply of electricity and heat** | 2.8 |
|  |  |

In the whole year, the province produced 2.7104 million smart TV sets, an increase of 16.1% over the previous year; ten kinds of non-ferrous metals were 1.5912 million tons, an increase of 10.7%; 212,600 tons of dairy products, up by 20.4%.

**Table 6 Output and Growth Rate of Major Industrial Products above Designated Size in 2022**

| **Indicator name (unit)** | **Absolute number** | **Year-on-year growth (%)** |
| --- | --- | --- |
| **Power generation (100 million kWh)** | 2184.06 | -2.6 |
| **# Wind power** | 105.33 | 3.0 |
| **Solar power generation** | 93.73 | 27.4 |
| **Biomass and garbage power generation** | 21.14 | 16.7 |
| **Raw coal (10,000 tons)** | 12813.63 | -4.0 |
| **Phosphate rock (containing 30% of phosphorus pentoxide) (10,000 tons)** | 1902.15 | -23.0 |
| **Beverage wine (thousands of liters)** | 97.28 | -8.9 |
| **Cigarettes (100 million)** | 1177.59 | 0.5 |
| **Chinese patent medicine (10,000 tons)** | 6.34 | -8.0 |
| **Dairy products (10,000 tons)** | 21.26 | 20.4 |
| **Chili products (10,000 tons)** | 32.54 | -37.7 |
| **Guitar (10,000)** | 107.23 | -31.4 |
| **Agricultural nitrogen, phosphorus and potassium chemical fertilizers (converted into pure) (10,000 tons)** | 247.37 | -18.7 |
| **Rubber tire casing (10,000 pieces)** | 659.42 | -7.9 |
| **Cement (10,000 tons)** | 6428.02 | -31.3 |
| **Pig iron (10,000 tons)** | 380.63 | -1.0 |
| **Steel (10,000 tons)** | 607.33 | -25.3 |
| **Ferroalloy (10,000 tons)** | 198.00 | -23.4 |
| **Ten kinds of non-ferrous metals (10,000 tons)** | 159.12 | 10.7 |
| **Primary aluminum (electrolytic aluminum) (10,000 tons)** | 140.61 | 5.7 |
| **Household refrigerators (10,000 units)** | 156.64 | -3.3 |
| **Integrated circuit (10,000 pieces)** | 38842.19 | -21.5 |
| **Electronic components (100 million)** | 68.14 | -10.4 |
| **Vehicles (10,000)** | 6.57 | -7.5 |
| **Smart TV (10,000 sets)** | 271.04 | 16.1 |

At the end of the year, the installed capacity of power generation in the whole province was 80.8727 million kilowatts, an increase of 6.8% over the end of the previous year. Among them, the installed capacity of thermal power was 37.9272 million kilowatts, an increase of 6.2%; the installed capacity of hydropower was 22.8232 million kilowatts, basically the same as the previous year; the installed capacity of grid-connected wind power was 5.9196 million kilowatts, an increase of 2.0%; The installed capacity of grid-connected solar power was 14.2027 million kilowatts, an increase of 24.9%.

In the whole year, the business income of industrial enterprises above the provincial scale reached 1064.950 billion yuan, an increase of 3.6% over the previous year. The total profits of industrial enterprises above designated size in the year totaled 132.032 billion yuan, up by 21.5% over the previous year. The annual profit margin of operating income was 12.4%, 1.8 percentage points higher than that of the previous year. The cost per 100 yuan of business income was 75.77 yuan, down 2.14 yuan. At the end of the year, the asset-liability ratio of industrial enterprises above designated size was 61.0%, down 1.3 percentage points from the end of last year.

At the end of the year, there were 2420 qualified general contracting and specialized contracting construction enterprises in the province, an increase of 249 over the end of the previous year. Among them, there were 144 construction enterprises with special-grade and first-grade qualifications, an increase of 14, and 1057 enterprises with second-grade qualifications, an increase of 110. There are 1219 three-level qualified enterprises and other qualified enterprises, an increase of 125.

IV. Investment in fixed assets

The investment in fixed assets of the whole province (excluding farmers) decreased by 5.1% over the previous year. Among them, investment in the primary industry decreased by 0.4%, investment in the secondary industry increased by 9.1%, and investment in the tertiary industry decreased by 10.0%. Industrial investment increased by 9.1%.

**Table 7 Growth rate of investment in fixed assets by industry (excluding farmers) in 2022**

| **Indicator name** | **Year-on-year growth (%)** |
| --- | --- |
| **Investment in fixed assets (excluding farmers)** | -5.1 |
| **# Agriculture, forestry, animal husbandry and fishery** | 4.7 |
| **Mining industry** | 5.9 |
| **# Coal mining and washing industry** | 6.0 |
| **Manufacturing** | 28.0 |
| **# Wine, Beverage and Refined Tea Manufacturing** | 35.3 |
| **Chemical raw materials and chemical products manufacturing industry** | 0.9 |
| **Pharmaceutical manufacturing** | 42.9 |
| **Ferrous metal smelting and calendering industry** | 26.9 |
| **Non-ferrous metal smelting and calendering industry** | -12.1 |
| **Production and supply of electricity, heat, gas and water** | -19.7 |
| **Transportation, storage and postal services** | 19.6 |
| **Management of water conservancy, environment and public facilities** | -3.8 |
| **Education** | 1.0 |
| **Health and Social Work** | 3.3 |
| **Information transmission, software and information technology services** | 26.7 |
| **Leasing and business services** | -35.5 |
| **Scientific research and technical services** | 95.8 |

V. Market Consumption

The total retail sales of social consumer goods in the whole province decreased by 4.5% over the previous year. According to the statistics of business places, the retail sales of consumer goods in cities and towns decreased by 4.9%, and the retail sales of consumer goods in rural areas decreased by 1.4%. According to the statistics of consumption types, the retail sales of commodities decreased by 4.7%, and the catering income decreased by 2.4%.

In the whole year, the retail sales of corporate enterprises (units) above designated size in the whole province decreased by 4.2% over the previous year. Among them, the retail sales of Chinese and Western medicines increased by 9.2%, petroleum and products by 4.7%, household appliances and audio-visual equipment by 0.8%, grain, oil and food by 1.2%, and cultural and office supplies by 6.4%. In the whole year, the retail sales of commodities realized by corporate enterprises (units) above designated size through public networks decreased by 6.7% over the previous year.

**Table 8 Growth rate of retail sales of corporate enterprises (units) above designated size in 2022**

| **Indicator name** | **Year-on-year growth (%)** |
| --- | --- |
| **Retail sales of corporate enterprises (units) above designated size** | -4.2 |
| **# Cereals, oils and foodstuffs** | -1.2 |
| **Alcohol and tobacco** | -10.1 |
| **Clothing, shoes and hats, knitted textiles** | -26.8 |
| **Cosmetics** | -1.0 |
| **Gold, silver and jewelry** | -12.7 |
| **Daily necessities** | -18.5 |
| **Sports and entertainment** | -6.0 |
| **Books, newspapers and magazines** | -1.7 |
| **Household appliances and audio and video equipment** | 0.8 |
| **Chinese and Western medicines** | 9.2 |
| **Cultural office supplies** | -6.4 |
| **Furniture** | -22.4 |
| **Communication equipment** | -15.2 |
| **Petroleum and its products** | 4.7 |
| **Construction and decoration materials** | -15.7 |
| **Cars** | -6.0 |
|  |  |

VI. Foreign Economy

The total import and export volume of the province in the whole year was 79.286 billion yuan, an increase of 21.2% over the previous year. The total export value was 51.981 billion yuan, up 6.7%, and the total import value was 27.306 billion yuan, up 63.6%. Of the total export volume, the general trade was 38.091 billion yuan, up 2.2%, and the processing trade was 9.359 billion yuan, up 37.4%. Of the total imports, general trade was 14 billion 876 million yuan, an increase of 74.4%; Processing trade was 7.372 billion yuan, an increase of 55.2%.

**Table 9 Total Import and Export Volume and Its Growth Rate in 2022**

| **Indicator name** | **Absolute number (100 million yuan)** | **Year-on-year growth (%)** |
| --- | --- | --- |
| **Total imports and exports** | **792.86** | **21.2** |
| **Total imports** | 273.06 | 63.6 |
| **# General trade** | 148.64 | 74.4 |
| **Processing trade** | 73.72 | 55.2 |
| **Total exports** | 519.81 | 6.7 |
| **# General trade** | 380.91 | 2.2 |
| **Processing trade** | 93.59 | 37.4 |
|  |  |  |

In the whole year, 105 foreign-invested enterprises were newly established in the province, and the actual amount of foreign capital used was 531 million US dollars. The total investment of foreign-invested enterprises at the end of the year was 215.568 billion US dollars. The registered capital at the end of the period is 166.964 billion US dollars, of which 153.885 billion US dollars are subscribed by foreign parties.

VII. Transportation

At the end of the year, the provincial highway traffic mileage was 209600 kilometers, an increase of 1.2% over the end of the previous year. Among them, 8331 kilometers of expressways were open to traffic, an increase of 4.0%. At the end of the year, the length of inland waterways was 3,954 km.

The total volume of railway, highway and waterway cargo transportation in the whole year was 94998 5900 tons, down 2.1% from the previous year. The cargo turnover was 141.727 billion ton-kilometers, down by 1.3% over the previous year. Civil aviation cargo and postal throughput was 84 thousand and 800 tons, down 29.3% from the previous year.

**Table 10 Cargo Transport Volume and Growth Rate of Various Transport Modes in 2022**

| **Indicator name (unit)** | **Absolute number** | **Year-on-year growth (%)** |
| --- | --- | --- |
| **Total volume of goods transported (10,000 tons)** | 94998.59 | -2.1 |
| **Railway** | 6671.98 | -8.3 |
| **Highway** | 87870.39 | -1.4 |
| **Water transport** | 456.21 | -18.5 |
| **Cargo turnover (100 million ton-kilometers)** | 1417.27 | -1.3 |
| **Railway** | 680.18 | -0.8 |
| **Highway** | 722.91 | -0.5 |
| **Water transport** | 14.18 | -40.3 |
| **Cargo and mail throughput of civil aviation (10,000 tons)** | 8.48 | -29.3 |
|  |  |  |

The total volume of railway, highway and waterway passenger transport in the whole year was 20322 5900, down 21.4% from the previous year. The passenger turnover was 31.987 billion person-kilometers, down by 22.0%. Civil aviation handled 12.3157 million passengers, down 44.6% from the previous year.

**Table 11 Passenger traffic volume completed by various modes of transport in 2022 and its growth rate**

| **Indicator name (unit)** | **Absolute number** | **Year-on-year growth (%)** |
| --- | --- | --- |
| **Total volume of passenger transport (10,000 persons)** | 20322.59 | -21.4 |
| **Railway** | 4580.23 | -29.3 |
| **# High speed rail** | 3763.78 | -26.4 |
| **Highway** | 15537.09 | -18.2 |
| **Water transport** | 205.27 | -44.5 |
| **Passenger turnover (100 million person-km)** | 319.87 | -22.0 |
| **Railway** | 207.20 | -18.9 |
| **# High speed rail** | 166.14 | -15.3 |
| **Highway** | 112.24 | -27.1 |
| **Water transport** | 0.43 | -52.0 |
| **Civil aviation passenger throughput (10,000 person-times)** | 1231.57 | -44.6 |
|  |  |  |

VIII. Postal Communication

In the whole year, the total business volume of the postal industry in the province was 9.627 billion yuan, an increase of 9.6% over the previous year. In the whole year, 24.0881 million pieces of mail business were completed, down 2.9% from the previous year; 492 million pieces of express delivery were received and sent, up 23.7%; Express business revenue was 7.267 billion yuan, an increase of 9.0%.

The total volume of telecommunications business in the whole province was 44.76 billion yuan, an increase of 25.4% over the previous year. At the end of the year, there were 44.479 million mobile phone users and 38,000 Gbps of Internet bandwidth, up by 35.7%. The length of optical cable lines was 1.67 million km, an increase of 24.0%; The number of 5G base stations has reached 84693.

Ix. Finance and banking

The total fiscal revenue of the province in the whole year was 319.269 billion yuan, a decrease of 6.5% over the previous year, and an increase of 6.2% in the same caliber after deducting the VAT rebate factor (the same below). The general public budget revenue was 188.641 billion yuan, a decrease of 4.2% over the previous year and an increase of 6.8% over the same caliber. Among them, tax revenue was 102 billion 176 million yuan, down 13.2%, an increase of 5.3% in the same caliber.

In the whole year, the general public budget expenditure of the province was 585 billion 136 million yuan, an increase of 4.7% over the previous year. Of this, transportation expenditure was 37.248 billion yuan, an increase of 10.7%; health expenditure was 58.359 billion yuan, an increase of 7.7%; social security and employment expenditure was 73.846 billion yuan, an increase of 7.2%; culture, tourism, sports and media expenditure was 12.358 billion yuan, an increase of 5.5%; Expenditure on education was 115 billion 533 million yuan, an increase of 2.3%.

At the end of the year, the balance of RMB deposits in financial institutions in the whole province was 3276.105 billion yuan, an increase of 9.0% over the same period last year. Among them, household deposits 16320 50 million yuan, an increase of 14.8%. At the end of the year, the balance of RMB loans of financial institutions was 40223 109 million yuan, an increase of 12.3% over the same period last year. Among them, household loans amounted to 1212.711 billion yuan, an increase of 8.5%.

**Table 12 Balance and Growth Rate of RMB Deposits and Loans of Financial Institutions at the End of 2022**

| **Indicator name** | **Absolute number (100 million yuan)** | **Year-on-year growth (%)** |
| --- | --- | --- |
| **Balance of various deposits** | **32761.05** | **9.0** |
| **# Domestic deposits** | 32751.08 | 9.0 |
| **Household deposits** | 16320.50 | 14.8 |
| **Deposits of non-financial enterprises** | 8604.83 | 3.9 |
| **Balance of various loans** | **40223.09** | **12.3** |
| **# Domestic loans** | 40222.46 | 12.3 |
| **Household loans** | 12127.11 | 8.5 |
| **Short-term loan** | 3303.65 | 10.7 |
| **Medium and long-term loans** | 8823.45 | 7.7 |
| **Loans to enterprises (institutions)** | 28095.34 | 14.0 |
| **Short-term loan** | 3374.57 | 18.3 |
| **Medium and long-term loans** | 23625.71 | 12.2 |

In the whole year, the original premium income of insurance companies in the whole province was 50.424 billion yuan, an increase of 1.6% over the previous year. Of this total, the premium of property insurance was 22.993 billion yuan, and that of life insurance was 27.431 billion yuan. The annual compensation expenditure was 21.808 billion yuan, an increase of 5.4% over the previous year. Among them, 14.374 billion yuan was paid for property insurance and 7.434 billion yuan for life insurance.

X. People's livelihood and social security

In the whole year, the per capita disposable income of residents in the whole province was 25508 yuan, an increase of 6.3% over the previous year. By permanent residence, the per capita disposable income of urban residents was 41086 yuan, an increase of 4.8%; The per capita disposable income of rural residents was 13707 yuan, an increase of 6.6%.

**Table 13 Composition and Growth Rate of Per Capita Disposable Income of Urban and Rural Residents in 2022**

| **Indicator name** | **Absolute number (yuan)** | **Year-on-year growth (%)** |
| --- | --- | --- |
| **Per capita disposable income of urban residents** | 41086 | 4.8 |
| **Wage income** | 23448 | 4.3 |
| **Net income from operations** | 7232 | 7.1 |
| **Net income from property** | 3309 | -2.8 |
| **Net income transferred** | 7097 | 8.2 |
| **Per capita disposable income of rural residents** | 13707 | 6.6 |
| **Wage income** | 5585 | 4.8 |
| **Net income from operations** | 4219 | 7.8 |
| **Net income from property** | 117 | -6.4 |
| **Net income transferred** | 3787 | 8.5 |

The annual per capita consumption expenditure of residents in the whole province was 17939 yuan, down 0.1% from the previous year. By permanent residence, the per capita consumption expenditure of urban residents was 24230 yuan, down 4.4%; The per capita consumption expenditure of rural residents was 13172 yuan, an increase of 4.9%.

At the end of the year, every 100 urban households owned 54.85 household automobiles, up 3.7% from the end of the previous year, and 50.86 air conditioners, up 5.9%. At the end of the year, every 100 rural households owned 26.24 household automobiles, up 11.4%, and 57.9 motorcycles, down 1.4%. There were 81.3 water heaters, up 4.8%.

At the end of the year, the per capita housing area of urban residents in the province was 42.64 square meters, an increase of 0.62 square meters over the end of the previous year. The per capita housing area of rural residents is 45.44 square meters, a decrease of 4.39 square meters.

**Table 14 Number of Durable Consumer Goods Owned per 100 Households and Its Growth Rate at the End of 2022**

| **Indicator name (unit)** | **Absolute number** | **Increase over the end of last year (%)** |
| --- | --- | --- |
| **Number of durable consumer goods owned per 100 urban households** |  |  |
| **Water heater (set)** | 101.73 | 1.2 |
| **Air conditioner (set)** | 50.86 | 5.9 |
| **Computer (set)** | 53.38 | 2.8 |
| **Mobile phone (unit)** | 299.28 | 0.2 |
| **Color TV set** | 106.27 | 1.0 |
| **Refrigerator (cabinet) (set)** | 105.48 | 1.3 |
| **Motorcycle (unit)** | 22.61 | 1.2 |
| **Family car (unit)** | 54.85 | 3.7 |
| **Number of durable consumer goods owned per 100 rural households** |  |  |
| **Washing machine (set)** | 100 | 1.5 |
| **Motorcycle (unit)** | 57.9 | -1.4 |
| **Family car (unit)** | 26.24 | 11.4 |
| **Color TV set** | 100 | 1.6 |
| **Water heater (set)** | 81.3 | 4.8 |
| **Fixed telephone (unit)** | 0.21 | -50.3 |
| **Mobile phone (unit)** | 319.8 | 2.2 |
| **Refrigerator (cabinet) (set)** | 101.8 | 2.9 |

At the end of the year, 19.3553 million urban and rural residents participated in basic old-age insurance, an increase of 0.4% over the end of the previous year. The number of urban workers participating in basic old-age insurance was 7.707 million, an increase of 1.9%. 3.387 million people participated in unemployment insurance, an increase of 5.5%. The number of people participating in basic medical insurance was 42.2123 million. The number of people participating in industrial injury insurance was 5.9384 million, an increase of 12.1%.

At the end of the year, there were 578,900 urban residents receiving the minimum living allowance in the province; the monthly per capita allowance was 679 yuan, an increase of 3.5% over the previous year. At the end of the year, 1.7019 million rural residents received subsistence allowances; The annual per capita security standard was 5292 yuan, an increase of 15.8% over the previous year.

At the end of the year, there were 1030 social service institutions providing accommodation in the province, including 937 old-age institutions and 34 child welfare and relief and protection institutions. At the end of the year, there were 92,400 beds in civil affairs institutions providing accommodation, including 83,200 beds in old-age care institutions and 4,254 beds in child welfare and relief and protection institutions.

Xi. Tourism and culture

In the whole year, the province received 492 million tourists, with a total tourism income of 524.564 billion yuan.

At the end of the year, there were 9 5A-level tourist attractions and 143 4A-level tourist attractions in the province. At the end of the year, there were 81 key cultural relics protection units, 437 key villages (towns) of rural tourism above grade, and 8957 standardized units of rural tourism above grade. At the end of the year, the number of guest rooms was 884,100, and the number of beds in guest rooms was 1,436,200.

At the end of the year, there were 101 art performance groups, 23 art performance venues, 133 museums, 99 public libraries, 1701 mass art galleries and cultural centers (stations) in the province. At the end of the year, the comprehensive population coverage rate of television was 99.24%, and that of radio was 99.02%. 136 million copies of books and 14.25 million copies of periodicals were published in the whole year.

XII. Education and Science and Technology

At the end of the year, there were 6,470 primary schools with 3,915,400 students, 1,902 junior middle schools with 1,898,500 students, and 494 senior middle schools with 950,600 students. There are 183 secondary vocational schools with 524,400 students, 75 institutions of higher learning with 894,700 students, and 10 postgraduate training units with 36,800 postgraduates.

**Table 15 Enrollment, Enrolment and Growth Rate of Enrolled Students of All Levels and Types of Schools in 2022**

| **Indicator name** | **Absolute number (10,000 persons)** | **Year-on-year growth (%)** |
| --- | --- | --- |
| **Enrollment of all kinds of schools at all levels** |  |  |
| **# Graduate students** | 1.34 | 9.8 |
| **Regular institutions of higher learning** | 27.93 | -4.0 |
| **Adult Higher Education** | 2.51 | -13.4 |
| **Secondary Vocational Education** | 28.38 | 79.8 |
| **Ordinary high school** | 30.85 | -6.8 |
| **Junior middle school** | 66.58 | 3.6 |
| **Ordinary primary school** | 60.92 | -2.4 |
| **Number of students in schools of all levels and types** |  |  |
| **# Graduate students** | 3.68 | 16.1 |
| **Regular institutions of higher learning** | 89.47 | 1.5 |
| **Adult Higher Education** | 7.31 | -16.0 |
| **Secondary Vocational Education** | 52.44 | 31.9 |
| **Ordinary high school** | 95.06 | -1.6 |
| **Junior middle school** | 189.85 | 5.5 |
| **Ordinary primary school** | 391.54 | -1.2 |

At the end of the year, the province had five national science and technology cooperation bases, 93 academician workstations, six national key laboratories and one national key laboratory. In the whole year, 855.4 technology contracts were registered, up by 53.0% over the previous year, and the transaction value of contracts was 39.073 billion yuan, up by 35.1%. 193 scientific and technological achievements at or above the provincial and ministerial levels were registered, down by 3.0%; 293 patents were granted, down 25.2%.

XIII. Health and Physical Education

At the end of the year, there were 29150 medical and health institutions in the province, including 2826 hospitals and health centers; There are 344 professional public health institutions, including 101 centers for disease control and prevention. At the end of the year, there were 309703 beds in medical and health institutions, including 290,489 beds in hospitals and health centers. At the end of the year, there were 321,400 health technicians, including 109,400 practicing (assistant) doctors and 147,100 registered nurses.

**Table 16 Medical and health institutions, beds, health technicians and their growth rate at the end of 2022**

| **Indicator name (unit)** | **Absolute number** | **Increase over the end of last year (%)** |
| --- | --- | --- |
| **Health institutions (unit)** | 29150 | -0.5 |
| **# Hospitals and health centers** | 2826 | Flat |
| **Health technical personnel (10,000 persons)** | 32.14 | 3.9 |
| **# Medical Practitioner (Assistant)** | 10.94 | 3.8 |
| **Registered nurse** | 14.71 | 3.9 |
| **Beds in health institutions (PCs.)** | 309703 | 4.3 |
| **# Hospitals and health centers** | 290489 | 4.1 |
|  |  |  |

Guizhou athletes won 105 awards in major international and domestic sports competitions throughout the year. Among them, two world champions and 33 gold medals in the highest level competitions in China were won. There are 108,300 sports venues in the province.

XIV. Ecological Construction and Environment

At the end of the year, there were 88 nature reserves in the province. Among them, there are 11 national nature reserves. At the end of the year, the area of nature reserves was 847,000 hectares. The afforestation area of the whole year was 183,300 hectares, and the forest coverage rate of the whole province was 62.81% at the end of the year.

The proportion of days with good air quality in the central cities of the province was 99.1%. The excellent rate of water quality in the exit sections of major rivers is 100%. The sewage treatment rate of cities (counties) and the harmless treatment rate of domestic waste increased to 98.2% and 99.3% respectively. The energy consumption of 10000 yuan GDP decreased by 3.7% compared with the previous year. A total of 321400 hectares of soil erosion control area have been added.

Note:

1. The data in this bulletin are preliminary statistics.

2. The growth rate of GDP, industrial added value above scale and its classified items is calculated at comparable prices, which is the actual growth rate; Unless otherwise specified, other indicators are nominal growth rates calculated at current prices.

3. Gross regional product refers to the total value of all final products and services produced by all resident units in a region in a certain period of time.

4. The statistical scope of industries above designated size is industrial enterprises with annual main business income of 20 million yuan or more.

5. Investment in fixed assets (excluding farmers) refers to the total amount of work completed in a certain period of time in the form of money to build and purchase fixed assets and the related costs. The statistical scope of investment in fixed assets (excluding farmers) is investment in fixed assets projects with a total planned investment of more than 5 million yuan and investment in all real estate development projects.

6. Units above the quota in the retail statistics of social consumer goods refer to wholesale enterprises (units) with annual main business income of 20 million yuan or more, retail enterprises (units) with annual main business income of 5 million yuan or more, accommodation and catering enterprises (units) with annual main business income of 2 million yuan or more.

7. Consumer price index refers to the relative number reflecting the trend and degree of changes in the prices of consumer goods and services purchased by urban and rural residents in a certain period of time.

8. Due to the rounding of some data, there is a difference between the total and the sub-total.

Source:

The employment, social security and other data in this bulletin are from the Provincial Human Resources and Social Security Department; the market subject and patent data are from the Provincial Market Supervision and Administration Bureau; the afforestation area, forest coverage and nature reserve data are from the Provincial Forestry Bureau; the aquatic product output data are from the Provincial Department of Agriculture and Rural Areas; the import and export data are from Guiyang Customs; The data of actually used foreign capital and foreign economic and technological cooperation are from the Provincial Department of Commerce; the data of highway transportation and water transportation are from the Provincial Department of Transportation; the data of railway transportation are from the National Bureau of Statistics, which are full-caliber data; the data of civil aviation are from the Provincial Airport Group Co., Ltd.; the data of postal services are from the Provincial Postal Administration; the data of telecommunications are from the Provincial Communications Administration; the data of finance are from the Provincial Department of Finance; Financial data are from Guiyang Central Branch of the People's Bank of China; securities and futures data are from Guizhou Regulatory Bureau of China Securities Regulatory Commission; insurance data are from Guizhou Regulatory Bureau of China Banking and Insurance Regulatory Commission; medical insurance data are from Provincial Medical Security Bureau; urban and rural minimum living allowance and social service data are from Provincial Department of Civil Affairs; The data of tourism, art performance groups and venues are from the Provincial Department of Culture and Tourism; the data of television and radio are from the Provincial Radio and Television Bureau; the data of books and periodicals are from the Publicity Department of the Provincial Committee; the data of education are from the Provincial Department of Education; the data of science and technology are from the Provincial Department of Science and Technology; the data of medical and health are from the Provincial Health Commission; the data of sports are from the Provincial Sports Bureau; the data of environment are from the Provincial Department of Ecological Environment; The data of comprehensive control of soil erosion are from the Provincial Department of Water Resources. The data of urban (county) sewage treatment rate and harmless treatment rate of domestic waste are from the Provincial Housing and Construction Department. The data of installed capacity of power generation comes from the Provincial Electric Power Industry Association.

[[Back to top]](javascript:scroll(0,0)) [[Print this page]](javascript:window.print();) [[Close this page]](javascript:window.close())

Previous:[Statistical Communique on R & D Investment in Guizhou Province in 2022](http://hgk.guizhou.gov.cn/publish/articles/c7/2023/10/a1022/a1022.html?locationhref=http://hgk.guizhou.gov.cn/publish/channels/c7/c7_1psSuffix&pagesize=15&curpage=1&curainum=1)
Next:[Statistical Communique on R & D Investment in Guizhou Province in 2021](http://hgk.guizhou.gov.cn/publish/articles/c7/2022/10/a858/a858.html?locationhref=http://hgk.guizhou.gov.cn/publish/channels/c7/c7_1psSuffix&pagesize=15&curpage=1&curainum=3)

Sponsor: Guizhou Provincial Bureau of Statistics Technical Support: Guizhou Jiawang Technology Development Co., Ltd.

Record No.: Qian ICP 19000889 No.: -3

Current location:[Home page](http://hgk.guizhou.gov.cn/index.vhtml) > [Statistical publications](http://hgk.guizhou.gov.cn/publish/channels/c6/c6_1.html)> [Statistical Bulletin](http://hgk.guizhou.gov.cn/publish/channels/c7/c7_1.html)> [Statistical Bulletin on the National Economic and Social Development of Guizhou Province in 2017](http://hgk.guizhou.gov.cn/publish/articles/c7/2023/09/a406/a406.html)

Statistical Bulletin

Statistical Bulletin on the National Economic and Social Development of Guizhou Province in 2017

Date: 2018-04-04 Article Source: Provincial Bureau of Statistics No.: [[Big](javascript:doZoom(20)) [Medium](javascript:doZoom(16)) [Small](javascript:doZoom(12)) ]

Guizhou Provincial Bureau of Statistics National Bureau of Statistics Guizhou Investigation Team
(4 April 2018)
　　 
In 2017, under the strong leadership of the provincial Party Committee and the provincial government, the whole province adhered to the guiding ideology of socialism with Chinese characteristics in the new era of , conscientiously implemented the spirit of the Nineteenth National Congress of the Party and the spirit of General Secretary 's important speech in the delegation of Guizhou Province, comprehensively implemented the decision-making and deployment of the central and provincial Party committees and provincial governments, and adhered to the general tone of steady and progressive work. Adhere to the overall situation of economic and social development, adhere to the supply-side structural reform as the main line, adhere to the two bottom lines of development and ecology, vigorously promote the three strategic actions of poverty alleviation, big data and big ecology, actively adapt to the new normal, actively respond to new challenges, effectively resolve new contradictions, and the province's economic operation. New achievements have been made in economic and social development.
First, comprehensive
Preliminary accounting shows that in 2017, the GDP of the whole province was 13540 83 million yuan, an increase of 10.2% over the previous year. By industry, the added value of the primary industry was 202.078 billion yuan, up 6.7%; the added value of the secondary industry was 543.963 billion yuan, up 10.1%; the added value of the tertiary industry was 608.042 billion yuan, up 11.5%. The added value of the primary industry accounted for 14.9% of the GDP, and the added value of the secondary industry accounted for 40.2%. The added value of the tertiary industry accounted for 44.9%. Per capita GDP was 37956 yuan, an increase of 4710 yuan over the previous year.
Figure 1 GDP and its growth rate from 2013 to 2017
　　
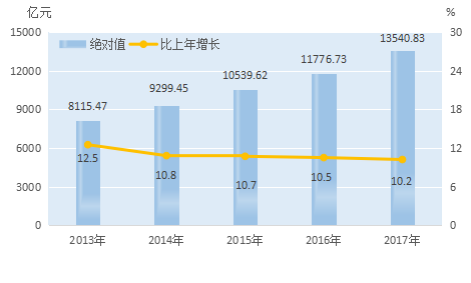

Figure 2 The proportion of the added value of the three industries in the GDP of the region from 2013 to 2017
　　
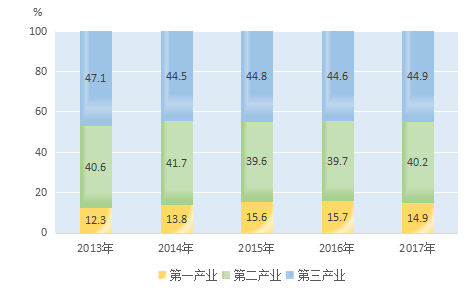

At the end of the year, the permanent population was 35.8 million, an increase of 250000 over the end of the previous year. Among them, the population aged 0-14 accounted for 22.4% of the permanent population at the end of the year, 67.2% of the population aged 15-64, and 10.4% of the population aged 65 and over. Regionally, the urban permanent population was 16.4752 million, accounting for 46.02% of the permanent population at the end of the year (the urbanization rate of the permanent population), an increase of 1.87 percentage points over the end of the previous year. The ratio of male to female (100 females) was 106.45. The annual number of births was 500,500, with a birth rate of 13.98 ‰; the number of deaths was 246,300, with a death rate of 6.88 ‰; and the natural growth rate was 7.10 ‰.
Table 1 Number and composition of resident population at the end of 2017

| Indicator name | Absolute number (10,000 persons) | As a percentage of the resident population at the end of the year Specific gravity (%) |
| --- | --- | --- |
| Resident population at the end of the year | 3580 | 100 |
| By urban and rural areas |  |  |
| Towns | 1647.52 | 46.02 |
| Rural | 1932.48 | 53.98 |
| By gender |  |  |
| Male | 1845.92 | 51.56 |
| Female | 1734.08 | 48.44 |
| By age |  |  |
| 0-14 years old (including less than 15 years old) | 802.28 | 22.41 |
| 15-64 years old (including less than 65 years old) | 2405.40 | 67.19 |
| 65 years and above | 372.32 | 10.40 |

769000 new jobs were created in cities and towns throughout the year, an increase of 1.5% over the previous year. Among them, 143 thousand and 900 unemployed people were re-employed, and 78 thousand and 200 people with employment difficulties were employed. At the end of the year, the registered urban unemployment rate was 3.23%.
At the end of the year, there were 2.4956 million market entities, an increase of 13.4% over the end of the previous year. Among them, 694900 new registered market entities were registered, an increase of 89.1% over the end of last year.
Table 2 Total number of newly registered market entities and their growth rate in 2017

| Indicator name | Absolute number (10,000 households) | Year-on-year growth (%) |
| --- | --- | --- |
| Total amount of newly registered market entities | 69.49 | 89.1 |
| # Domestic-funded enterprises | 13.07 | 33.2 |
| # Private enterprises | 12.02 | 34.0 |
| Foreign-funded enterprises | 0.03 | 29.8 |
| Individual industrial and commercial households and farmers' professional cooperatives | 56.39 | 111.3 |

II. Overcoming Poverty
The province took poverty alleviation as the overall situation of economic and social development, vigorously launched the "spring offensive", "summer contest" and "autumn offensive". At the end of the year, 2.8032 million rural poor people in the province were lifted out of poverty, 1.2369 million rural poor people were lifted out of poverty throughout the year, and the incidence of poverty dropped to 7.75%, down 3.17 percentage points from the end of last year.
Figure 3 Rural Poverty Population and Incidence of Poverty, 2013-2017
　　
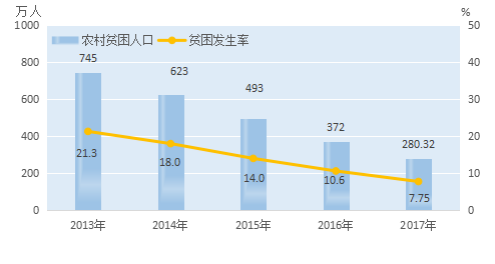

Start the three-year decisive battle of the rural "group-to-group" highway and build the "group-to-group" highway
25,000 kilometers. 763000 people were relocated and 15000 industrial poverty alleviation projects were implemented. 2.5745 million people enjoyed the "four-fold medical security", 200000 rural dilapidated houses were renovated, and 832500 students from poor families were subsidized.
3. Agriculture
In 2001, the added value of agriculture, forestry, animal husbandry and fishery was 212.848 billion yuan, up by 6.5% over the previous year.
Table 3 The added value and growth rate of agriculture, forestry, animal husbandry and fishery in 2017

| Indicator name | Absolute number (100 million yuan) | Year-on-year growth (%) |
| --- | --- | --- |
| Added value of agriculture, forestry, animal husbandry and fishery | 2128.48 | 6.5 |
| Planting | 1287.17 | 7.4 |
| Forestry | 155.45 | 7.9 |
| Animal husbandry | 531.08 | 4.4 |
| Fishery | 47.07 | 7.4 |
| Agriculture, forestry, animal husbandry and fishery services | 107.71 | 3.9 |

In 2001, the added value of planting industry was 128.717 billion yuan, up by 7.4% over the previous year. The grain planting area was 45.7685 million mu, a decrease of 930500 mu over the previous year. Among them, the rice planting area is 9.9192 million mu, a decrease of 194,700 mu; the corn planting area is 10.7291 million mu, a decrease of 375,800 mu. Among the cash crops, the planting area of vegetables and edible fungi is 17.2196 million mu, an increase of 1.463 million mu; the planting area of garden fruits is 5.9379 million mu, an increase of 1.0755 million mu; the planting area of tea is 7.153 million mu, an increase of 555,700 mu; The planting area of Chinese herbal medicines was 2.8593 million mu, an increase of 334,800 mu.
Table 4 Planting area and growth rate of grain crops in 2017

| Indicator name | Planting area (ten thousand mu) | Year-on-year growth (%) |
| --- | --- | --- |
| Food crops |  |  |
| # Rice | 991.92 | -1.9 |
| Corn | 1072.91 | -3.4 |
| Wheat | 334.23 | -7.8 |

Table 5 Planting area of cash crops and its growth rate in 2017

| Indicator name | Planting area (ten thousand mu) | Year-on-year growth (%) |
| --- | --- | --- |
| Cash crop |  |  |
| # Vegetables and Edible Fungi | 1721.96 | 9.3 |
| Fruit | 593.79 | 22.1 |
| Tea | 715.30 | 8.4 |
| Chinese herbal medicine | 285.93 | 13.3 |

The total grain output of the year was 11.7854 million tons. Among them, the output of rice was 4.237 million tons and the output of corn was 3.1356 million tons. Among the main cash crops, the output of tea was 176,500 tons, an increase of 24.9% over the previous year; The output of Chinese herbal medicines was 522,100 tons, an increase of 21.3%.
In 2001, the added value of forestry was 15.545 billion yuan, up by 7.9% over the previous year. The afforestation area is 10.002 million mu, and the output of commercial timber is 2.4855 million cubic meters.
In 2001, the added value of animal husbandry was 53.108 billion yuan, up by 4.4% over the previous year. The output of pork, beef, mutton and poultry was 2.0275 million tons, up by 4.0% over the previous year; the output of poultry eggs was 186,900 tons, up by 2.1%; the output of milk was 65,600 tons, up by 2.7%. At the end of the year, the number of live pigs was 15.9689 million, an increase of 6.6% over the end of the previous year; the number of cattle was 4.9235 million, a decrease of 5.0% over the end of the previous year. 18.2515 million pigs were slaughtered in the whole year, an increase of 3.7% over the previous year; 1,509,900 cattle were sold, an increase of 7.3%.
The added value of fishery in the whole year was 4.707 billion yuan, an increase of 7.4% over the previous year. The output of aquatic products was 299,600 tons, an increase of 3.4% over the previous year. Among them, the output of aquaculture products was 286 thousand and 400 tons, an increase of 3.8%.
The total power of agricultural machinery in the whole year was 28.07 million kilowatts, and the water-saving irrigation area increased by 100,100 mu.
IV. Industry and Construction
At the end of the year, there were 5637 industrial corporate enterprises above designated size, an increase of 590 over the end of the previous year. Among them, there are 7 and 2 enterprises whose annual main business income exceeds 10 billion yuan and 50 billion yuan respectively.
In the whole year, the added value of industries above designated size was 430.480 billion yuan, an increase of 9.5% over the previous year. Among them, the added value of light industry was 170.859 billion yuan, an increase of 12.2% over the previous year, accounting for 39.7% of the added value of industries above scale. The added value of heavy industry was 259.622 billion yuan, an increase of 7.7%, accounting for 60.3% of the added value of industries above scale.
Figure 4 Industrial added value above designated size and its growth rate from 2013 to 2017
　　
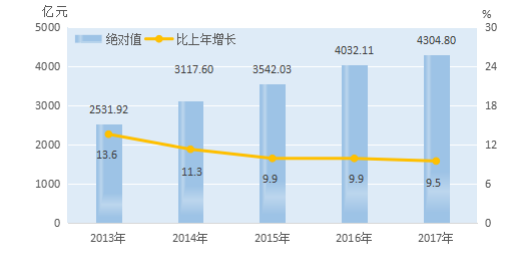

Among the industries above the scale, the added value of the wine, beverage and refined tea manufacturing industry increased by 13.5% over the previous year, that of the power, thermal production and supply industry increased by 13.0%, that of the tobacco products industry increased by 2.0%, that of the coal mining and washing industry decreased by 4.9%, and that of the four traditional industries of coal, electricity, tobacco and alcohol totaled 242.291 billion yuan. It accounts for 56.3% of the industrial added value above the scale. Among them, the added value of wine, beverage and refined tea manufacturing industry is 89.715 billion yuan, accounting for 20.8% of the industrial added value above the scale, which is the largest pillar industry.
Table 6 The added value and growth rate of major industries above designated size in 2017

| Indicator name | Absolute number (100 million yuan) | Year-on-year growth (%) |
| --- | --- | --- |
| Industrial added value above designated size | 4304.80 | 9.5 |
| # Coal mining and washing industry | 777.40 | -4.9 |
| Non-metallic mining and dressing industry | 108.88 | 9.0 |
| Agricultural and sideline food processing industry | 77.78 | 11.8 |
| Wine, beverage and refined tea manufacturing | 897.15 | 13.5 |
| Tobacco products industry | 288.04 | 2.0 |
| Chemical raw materials and chemical products manufacturing industry | 166.58 | 4.2 |
| Pharmaceutical manufacturing | 148.30 | 21.3 |
| Non-metallic mineral products industry | 358.69 | 6.5 |
| Ferrous metal smelting and calendering industry | 84.42 | -4.6 |
| Non-ferrous metal smelting and calendering industry | 131.48 | 10.5 |
| Computer, communication and other electronic equipment manufacturing | 118.64 | 86.3 |
| Production and supply of electricity and heat | 460.31 | 13.0 |
| Automobile manufacturing | 75.60 | 19.1 |

The added value of high-tech industries in the whole year increased by 39.9% over the previous year, accounting for 8.1% of the added value of industries above scale, an increase of 1.3 percentage points over the previous year. The added value of computer, communication and other electronic equipment manufacturing industry, pharmaceutical manufacturing industry and automobile manufacturing industry was 11.864 billion yuan, 14.830 billion yuan and 7.560 billion yuan respectively, up 86.3%, 21.3% and 19.1% respectively over the previous year, accounting for 2.8%, 3.4% and 1.8% of the added value of industries above scale.
Table 7 Output and Growth Rate of Major Industrial Products above Designated Size in 2017

| Indicator name (unit) | Absolute number | Year-on-year growth (%) |
| --- | --- | --- |
| Power generation (100 million kWh) | 1856.53 | 3.5 |
| Phosphate rock (containing 30% of phosphorus pentoxide) (10,000 tons) | 4817.00 | 10.9 |
| Beverage wine (thousands of liters) | 143.90 | 6.5 |
| # White wine | 45.21 | 10.0 |
| Cigarettes (100 million) | 1076.05 | -7.3 |
| Chinese patent medicine (10,000 tons) | 9.78 | 15.8 |
| Multicolor printed matter (ten thousand folio color order) | 466.93 | 4.8 |
| Coke (10,000 tons) | 510.21 | -19.1 |
| Agricultural nitrogen, phosphorus and potassium chemical fertilizers (converted into pure) (10,000 tons) | 538.47 | -1.1 |
| Rubber tire casing (10,000 pieces) | 497.59 | 0.7 |
| Cement (10,000 tons) | 11356.51 | 8.2 |
| Pig iron (10,000 tons) | 343.71 | -4.6 |
| Steel (10,000 tons) | 495.72 | 1.1 |
| Ferroalloy (10,000 tons) | 251.35 | -4.0 |
| Ten kinds of non-ferrous metals (10,000 tons) | 109.35 | 53.5 |
| Primary aluminum (electrolytic aluminum) (10,000 tons) | 101.85 | 63.5 |
| Household refrigerators (10,000 units) | 125.90 | 0.9 |
| Integrated circuit (10,000 pieces) | 15175.89 | 29.7 |
| Vehicle (unit) | 89878 | -0.7 |
| Color TV sets (10,000 sets) | 205.45 | 7.4 |
| Smartphones (10,000 units) | 1855.66 | 693.0 |

At the end of the year, the installed capacity of electric power was 58.4252 million kilowatts, an increase of 3.024 million kilowatts over the end of the previous year. Among them, the installed capacity of hydropower was 21 million 192 thousand and 300 kilowatts, an increase of 403 thousand kilowatts; The installed capacity of wind power was 3.6338 million kilowatts, an increase of 18,000 kilowatts.
The main business income of the industries above the designated scale in the whole year was 0.95 billion yuan 11300, an increase of 18.7% over the previous year, and the profits were 88.632 billion yuan, an increase of 46.4%. Profits and taxes reached 169.326 billion yuan, an increase of 31.6%. The rate of industrial production and sales was 97.2%.
At the end of the year, there were 1165 qualified general contracting and specialized contracting construction enterprises, an increase of 154 over the end of the previous year. Among them, there are 65 special-grade and first-grade construction enterprises, an increase of 2; 403 second-grade qualified enterprises, an increase of 69; There were 697 enterprises with three-level qualifications, an increase of 83. The total output value of construction industry was 293 billion 296 million yuan, an increase of 24.1%.
Figure 5 Total output value of construction industry and its growth rate from 2013 to 2017
　　
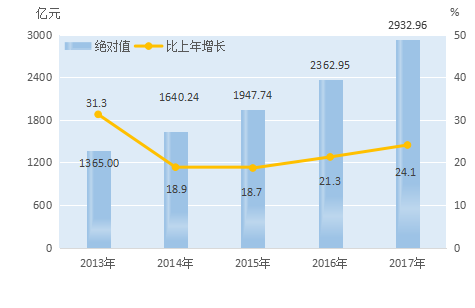

V. Investment in fixed assets
The annual investment in fixed assets was 1.55 trillion yuan, an increase of 20.1% over the previous year. The investment in the primary industry increased by 29.9% over the previous year, accounting for 2.5% of the province's total investment in fixed assets; the investment in the secondary industry increased by 5.8%, accounting for 16.7% of the province's total investment in fixed assets; Investment in the tertiary industry increased by 23.3%, accounting for 80.8% of the province's fixed assets investment. Infrastructure investment was 675 billion 729 million yuan, an increase of 25.5% over the previous year. The investment in high-tech industry was 26.387 billion yuan, an increase of 58.6% over the previous year. Investment in the six energy-intensive industries was 70.543 billion yuan, down 3.2% from the previous year.
Figure 6 The proportion of three industries investment in fixed assets investment in 2017
　　
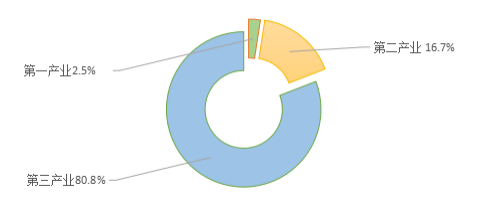

Table 8 Investment in Fixed Assets by Industry and Its Growth Rate in 2017

| Indicator name | Absolute number (100 million yuan) | Year-on-year growth (%) |
| --- | --- | --- |
| Investment in fixed assets |  |  |
| # Coal mining and washing industry | 294.68 | -8.7 |
| Wine, beverage and refined tea manufacturing | 169.97 | -9.9 |
| Chemical raw materials and chemical products manufacturing industry | 114.12 | -9.1 |
| Pharmaceutical manufacturing | 70.34 | 14.4 |
| Ferrous metal smelting and calendering industry | 43.37 | 7.4 |
| Non-ferrous metal smelting and calendering industry | 64.70 | 11.6 |
| Production and supply of electricity, heat, gas and water | 488.42 | 1.8 |
| Transportation, storage and postal services | 2362.30 | 7.7 |
| Information transmission, software and information technology services | 180.89 | 69.4 |
| Scientific research and technical services | 59.35 | 40.3 |
| Management of water conservancy, environment and public facilities | 3908.22 | 44.5 |
| Education | 428.13 | 49.4 |
| Health and Social Work | 189.42 | 79.1 |

The annual investment in real estate development was 220.100 billion yuan, an increase of 2.4% over the previous year. Among them, residential investment was 136 billion 533 million yuan, an increase of 9.8%. Housing construction area is 20385 43 thousand square meters, an increase of 0.2% over the previous year. The completed area was 11.717 million square meters, down 38.4% from the previous year. The sales area was 46.969 million square meters, an increase of 13.0% over the previous year. In the whole year, 432300 new housing units in shantytowns were renovated.
Table 9 Main indicators and growth rate of real estate development and sales in 2017

| Indicator name | Absolute number | Year-on-year growth (%) |
| --- | --- | --- |
| Real estate development investment (100 million yuan) (100 million yuan) | 2201.00 | 2.4 |
| # Residential | 1365.33 | 9.8 |
| Housing construction area (10,000 square meters) | 20385.43 | 0.2 |
| # Residential | 12789.65 | -0.4 |
| Completed housing area (10,000 square meters) | 1171.70 | -38.4 |
| # Residential | 785.00 | -38.8 |
| Housing sales area (10,000 square meters) | 4696.90 | 13.0 |
| # Residential | 3897.65 | 13.7 |

VI. Market and Price
In 2001, the total retail sales of consumer goods was 415.40 billion yuan, up by 12.0% over the previous year. According to the statistics of consumption types, the catering income was 36.497 billion yuan, an increase of 14.7% over the previous year; the retail sales of commodities was 378.902 billion yuan, an increase of 11.7%. According to the statistics of business places, the retail sales of consumer goods in cities and towns was 338 billion 854 million yuan, an increase of 11.8% over the previous year. The retail sales of rural consumer goods was 76.546 billion yuan, up by 12.9%.
Figure 7 Total retail sales of consumer goods and its growth rate from 2013 to 2017
　　
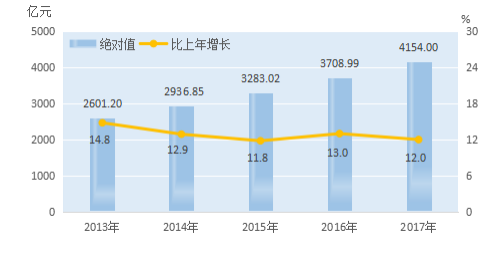

The retail sales of commodities by units above designated size was 214.730 billion yuan, up by 12.5% over the previous year. Among them, the retail sales of grain, oil and food were 15.028 billion yuan, an increase of 16.0%; the retail sales of clothing, shoes and hats, needles and textiles were 8.362 billion yuan, an increase of 6.9%; the retail sales of daily necessities were 5.357 billion yuan, an increase of 5.7%; the retail sales of household appliances and audio-visual equipment were 7.833 billion yuan, an increase of 7.0%; The retail sales of communication equipment was 1.689 billion yuan, an increase of 10.7%; the retail sales of construction and decoration materials was 1.263 billion yuan, an increase of 14.8%; The retail sales of automobiles was 73 billion 420 million yuan, an increase of 9.4%. The retail sales of enterprises (units) above designated size through public networks reached 7.456 billion yuan, an increase of 30.3% over the previous year.
Consumer prices for the whole year rose by 0.9% over the previous year. The ex-factory price of industrial producers rose by 7.2% over the previous year, and the purchasing price of industrial producers rose by 9.7%. The price of investment in fixed assets rose by 6.1% over the previous year, and the price of construction and installation projects rose by 7.3%.
VII. Foreign Economy
The total import and export volume of the year was 8.128 billion US dollars, an increase of 42.6% over the previous year. Of this, export was US $5.777 billion, up 21.8% over the previous year, and import was US $2.351 billion, up 145.8%. In the export market, exports to Hong Kong, Russia, the European Union and Latin America increased by 169.8%, 24.0%, 10.5% and 8.1% respectively, while exports to the United States and South Korea decreased by 20.0% and 48.2% respectively. Among the main export commodities, mechanical and electrical products were 3.220 billion US dollars, up 76.5% over the previous year; high-tech products were 2.521 billion US dollars, up 155.4%; flue-cured tobacco was 118 million US dollars, up 10.3%; Tea was 25 million US dollars, an increase of 24.6%.
Table 10 Total import and export volume and its growth rate in 2017

| Indicator name | Absolute ( $100 million) | Year-on-year growth (%) |
| --- | --- | --- |
| Total imports and exports | 81.28 | 42.6 |
| Total imports | 23.51 | 145.8 |
| # General trade | 8.91 | 60.9 |
| Processing trade | 12.18 | 344.9 |
| Total exports | 57.77 | 21.8 |
| # General trade | 40.75 | -0.7 |
| Processing trade | 16.11 | 195.4 |

The total amount of foreign capital actually utilized in the whole year was 3.891 billion US dollars, an increase of 21.0% over the previous year. The turnover of foreign economic and technological cooperation was 1.1 billion US dollars, an increase of 13.3%. Switzerland (Guizhou) Industrial Demonstration Park started construction and 10 projects were signed and landed. Six new commercial representative offices have been set up in Switzerland, Cambodia, India, Malaysia, Italy and Kyrgyzstan along the "The Belt and Road Initiative".
VIII. Tourism Development
The total number of tourists in the whole year was 74417 43 thousand, an increase of 40.0% over the previous year. Total tourism revenue was 711.681 billion yuan, an increase of 41.6%.
Figure 8 Total tourism revenue and its growth rate, 2013-2017
　　
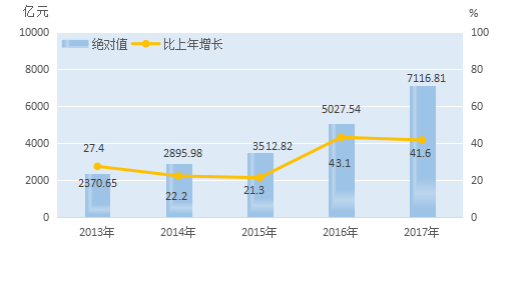

At the end of the year, there were 71 scenic spots. Among them, there are 18 national scenic spots and 53 provincial scenic spots. Five 5A tourist attractions, an increase of one over the end of last year; There are 95 4A-level tourist attractions, an increase of 27 over the end of last year. 131 provincial rural tourism demonstration zones; There are 1104 key villages for poverty alleviation through rural tourism.
IX. Transportation
At the end of the year, 194,400 kilometers of highways were open to traffic, an increase of 1.5% over the end of the previous year. The mileage of expressways open to traffic was 5834.50 kilometers, an increase of 7.4%.
The 94626 volume of railway, highway and waterway freight transport in the whole year was 81000 tons, an increase of 7.9% over the previous year. Of this, highway freight transport accounted for 89298 million tons, up by 8.6%, while railway freight transport accounted for 36.6423 million tons, down by 3.0%. Waterborne cargo transport totaled 16.6457 million tons, up 0.6%.
Table 11 Cargo transportation volume completed by various modes of transport and its growth rate in 2017

| Indicator name | Absolute number | Year-on-year growth (%) |
| --- | --- | --- |
| Total volume of goods transported (10,000 tons) | 94626.81 | 7.9 |
| Railway | 3664.23 | -3.0 |
| Highway | 89298.00 | 8.6 |
| Water transport | 1664.57 | 0.6 |
| Cargo turnover (100 million ton-kilometers) | 1544.52 | 12.2 |
| Railway | 490.87 | 6.5 |
| Highway | 1008.58 | 15.5 |
| Water transport | 45.07 | 6.4 |
| Cargo and mail throughput of civil aviation (10,000 tons) | 10.70 | 8.5 |

The total volume of railway, highway and waterway passenger transport in the whole year was 91465 8600, an increase of 2.7% over the previous year. Of this, the total passenger transport volume of highways was 83809 million, up by 2.0%, and that of railways was 54.5893 million, up by 15.5%. Waterborne passenger transport totaled 21.9793 million, an increase of 4.9%.
Table 12 Passenger traffic volume completed by various modes of transport and its growth rate in 2017

| Indicator name | Absolute number | Year-on-year growth (%) |
| --- | --- | --- |
| Total volume of passenger transport (10,000 persons) | 91465.86 | 2.7 |
| Railway | 5458.93 | 15.5 |
| Highway | 83809.00 | 2.0 |
| Water transport | 2197.93 | 4.9 |
| Passenger transport turnover (100 million person-km) | 704.32 | 7.7 |
| Railway | 233.66 | 13.9 |
| Highway | 463.93 | 4.7 |
| Water transport | 6.72 | 16.8 |
| Civil aviation passenger throughput (10,000 person-times) | 2457.65 | 31.2 |

At the end of the year, there were 6.961 million motor vehicles, an increase of 11.2% over the previous year. Among them, the number of civilian automobiles was 4.1571 million, an increase of 18.6%. Of the civilian automobiles, 1,892,700 were sedans, up 19.9%.
X. Big Data and Posts and Telecommunications
At the end of the year, the provincial Internet bandwidth was 6,730 Gbps, the length of optical cable lines was 900,000 kilometers, and the total number of Internet users was 35.0571 million.
In 2001, the total volume of postal services was 5.323 billion yuan, up by 24.7% over the previous year; The total volume of telecommunications business was 82 billion 529 million yuan, an increase of 146.2%. At the end of the year, the number of mobile phone users was 37.9227 million, an increase of 16.2% over the end of the previous year.
The total volume of express business in the whole year was 15781. 9 million pieces, an increase of 40.2% over the previous year. Express business revenue was 3.115 billion yuan, an increase of 43.0% over the previous year.
Figure 9 Total volume of express business from 2013 to 2017
　　
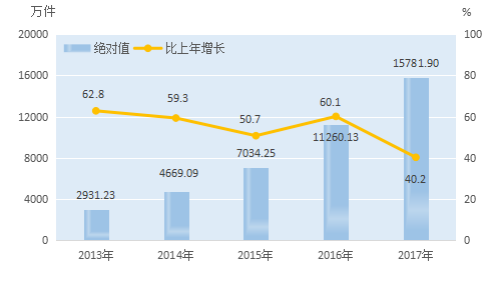

Xi. Finance and banking
The total fiscal revenue of the year was 265.002 billion yuan, an increase of 10.1% over the previous year. The general public budget revenue was 161 billion 364 million yuan, an increase of 7.2% over the previous year. Among them, tax revenue was 117 billion 955 million yuan, an increase of 10.9%.
Table 13 Main Indicators of Fiscal Revenue and Its Growth Rate in 2017

| Indicator name | Absolute number (100 million yuan) | Year-on-year growth (%) |
| --- | --- | --- |
| Total fiscal revenue | 2650.02 | 10.1 |
| # General public budget revenue | 1613.64 | 7.2 |
| # Tax revenues | 1179.55 | 10.9 |
| # Domestic VAT (including VAT) | 415.27 | 9.3 |
| Corporate income tax | 146.57 | 15.3 |
| Personal income tax | 48.53 | 38.4 |
| Urban maintenance and construction tax | 67.26 | 8.6 |
| Deed tax | 89.29 | -2.9 |
| Non-tax revenue | 434.09 | -1.5 |
| # Operating income of state-owned capital | 25.32 | -41.3 |

The annual general public budget expenditure was 460.457 billion yuan, an increase of 8.0% over the previous year. Of this, the expenditure on education was 90.351 billion yuan, an increase of 7.1% over the previous year, and the expenditure on agriculture, forestry and water resources was 60.126 billion yuan, a decrease of 4.5% over the previous year. Expenditure on social security and employment was 50.019 billion yuan, an increase of 36.2% over the previous year.
Table 14 Main Indicators of Fiscal Expenditure and Its Growth Rate in 2017

| Indicator name | Absolute number (100 million yuan) | Year-on-year growth (%) |
| --- | --- | --- |
| General public budget expenditure | 4604.57 | 8.0 |
| # Expenditure on general public services | 473.61 | 6.2 |
| Expenditure on education | 903.51 | 7.1 |
| Expenditure on science and technology | 88.29 | 27.4 |
| Expenditure on culture, sports and media | 64.51 | -4.2 |
| Expenditure on social security and employment | 500.19 | 36.2 |
| Expenditure on health care and family planning | 439.49 | 12.0 |
| Expenditure on energy conservation and environmental protection | 129.52 | 1.9 |
| Expenditure on agriculture, forestry and water | 601.26 | -4.5 |
| Transportation expenditure | 329.52 | 13.6 |
| Housing security expenditure | 244.58 | -16.2 |

At the end of the year, the balance of all kinds of RMB deposits in financial institutions was 26088 1.89 billion yuan, an increase of 231.809 billion yuan over the beginning of the year, an increase of 9.8% over the end of last year. Among them, the 26083 of domestic deposits was 1.16 billion yuan, an increase of 231 billion 788 million yuan over the beginning of the year. Household deposits amounted to 958.029 billion yuan, an increase of 104.769 billion yuan over the beginning of the year.
At the end of the year, the balance of various RMB loans of financial institutions was 20860 1.34 billion yuan, an increase of 300.254 billion yuan or 16.8% over the beginning of the year. Among them, the balance of agriculture-related loans was 874.48 billion yuan, an increase of 162.70 billion yuan over the beginning of the year; the balance of poverty alleviation loans was 393.08 billion yuan, an increase of 131.75 billion yuan over the beginning of the year; The loan balance of small and micro enterprises was 442 billion 310 million yuan, an increase of 98 billion 470 million yuan over the beginning of the year.
Table 15 Balance of RMB deposits and loans of financial institutions at the end of 2017 and its growth rate

| Indicator name | Absolute number (100 million yuan) | Increase over the end of last year (%) |
| --- | --- | --- |
| Balance of various deposits | 26088.89 | 9.8 |
| # Domestic deposits | 26083.16 | 9.8 |
| Household deposits | 9580.29 | 12.3 |
| Deposits of non-financial enterprises | 10059.57 | 8.2 |
| Balance of various loans | 20860.34 | 16.8 |
| # Domestic loans | 20859.62 | 16.8 |
| Household loans | 6200.63 | 19.0 |
| Short-term loan | 1527.82 | 23.9 |
| Medium and long-term loans | 4672.80 | 17.4 |
| Loans to non-financial enterprises and institutions | 14658.77 | 15.9 |
| Short-term loan | 2263.79 | 3.2 |
| Medium and long-term loans | 12125.57 | 19.6 |

The direct financing of stocks and bonds in the whole year was 14.940 billion yuan, down 82.5% from the previous year. At the end of the year, there were 27 domestic listed companies, an increase of 4 over the end of the previous year. The transaction value of securities bought and sold by securities companies as agents was 863.498 trillion yuan, an increase of 4.1% over the previous year.
In 2001, the premium income of insurance companies totaled 38.773 billion yuan, up by 20.7% over the previous year. Among them, property insurance premium income was 17 billion 926 million yuan, an increase of 17.1%; Life insurance premium income was 20.847 billion yuan, an increase of 24.0%. Indemnities and payments amounted to 15.381 billion yuan, up 16.9%. The premium income of agricultural insurance was 826 million yuan, an increase of 24.8%, providing 107.695 billion yuan of risk protection for 6.9791 million households.
Table 16 Main Indicators and Growth Rate of Insurance Industry in 2017

| Indicator name | Absolute number (100 million yuan) | Year-on-year growth (%) |
| --- | --- | --- |
| Original premium income | 387.73 | 20.7 |
| # Property insurance | 179.26 | 17.1 |
| # Motor Vehicle Insurance | 146.96 | 15.3 |
| Personal insurance | 208.47 | 24.0 |
| Accident Insurance | 14.22 | 23.6 |
| Health insurance | 37.65 | 35.2 |
| Life Insurance | 156.60 | 21.6 |
| Compensation expenses | 153.81 | 16.9 |
| # Property insurance | 91.38 | 15.0 |
| # Motor Vehicle Insurance | 77.00 | 14.5 |
| Personal insurance | 62.43 | 19.9 |
| Accident Insurance | 4.78 | 22.8 |
| Health insurance | 19.68 | 53.6 |
| Life Insurance | 37.97 | 7.4 |

XII. People's livelihood
The annual per capita disposable income of all residents was 16704 yuan, a nominal increase of 10.5% over the previous year. According to the permanent residence, the per capita disposable income of urban residents was 29080 yuan, an increase of 8.7% over the previous year. The per capita disposable income of rural residents was 8869 yuan, a nominal increase of 9.6% over the previous year.
Table 17 Per capita disposable income of urban and rural residents and its growth rate in 2017

| Indicator name | Absolute number (yuan) | Year-on-year growth (%) |
| --- | --- | --- |
| Per capita disposable income of urban residents | 29080 | 8.7 |
| # Wage income | 16552.58 | 7.8 |
| Net income from operations | 4721.76 | 10.3 |
| Net income from property | 2184.87 | 12.6 |
| Net income transferred | 5620.63 | 8.8 |
| Per capita disposable income of rural residents | 8869 | 9.6 |
| # Wage income | 3635.67 | 13.2 |
| Net income from operations | 3285.17 | 5.4 |
| Net income from property | 92.03 | 37.2 |
| Net income transferred | 1856.24 | 9.4 |

The per capita consumption expenditure of all residents was 12970 yuan, an increase of 8.7% over the previous year. By permanent residence, the per capita consumption expenditure of urban residents was 203.48 yuan, an increase of 6.0% over the previous year. The per capita consumption expenditure of rural residents was 8299 yuan, an increase of 10.2%.
Figure 10 Per capita consumption expenditure and its composition of urban residents in the province in 2017
　　
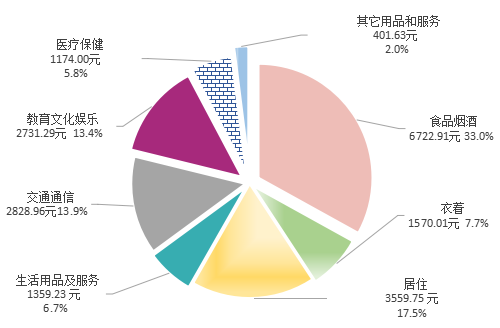

Figure 11 Per capita consumption expenditure and its composition of rural residents in the province in 2017
　　
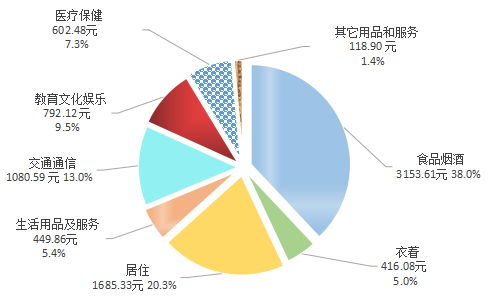

The per capita housing construction area of urban residents is 37.52 square meters, and the per capita housing construction area of rural residents is 34.54 square meters.
At the end of the year, every 100 urban households owned 35.0 household automobiles, 34.5 air conditioners and 256.7 mobile phones, up 4.3%, 11.8% and 3.7%, respectively, as compared with the end of the previous year. The number of refrigerators per 100 rural households was 82.3, up 6.1%, and the number of motorcycles per 100 rural households was 54.2, down 2.7%. There were 255.5 mobile phones, an increase of 6.2%.
At the end of the year, the number of urban employees participating in basic old-age insurance was 5.8817 million, an increase of 1.6459 million over the end of the previous year, of which 3.3782 million were enterprise employees, an increase of 279.5 million. The number of urban and rural residents participating in basic old-age insurance was 17.4855 million, an increase of 454700 over the end of last year. The number of people participating in unemployment insurance was 2 million 357 thousand and 100, an increase of 176 thousand and 100 over the end of last year. The number of employees participating in basic medical insurance was 4.1042 million, an increase of 206100 over the end of last year. The number of urban and rural residents participating in basic medical insurance was 5.9092 million, an increase of 71400 over the end of last year. The number of people participating in industrial injury insurance was 3.3248 million, an increase of 274600 over the end of last year, of which 928800 were migrant workers, an increase of 55100. The number of people participating in maternity insurance was 3.0403 million, an increase of 177600 over the end of last year.
At the end of the year, the number of urban minimum living security was 314400, and the annual per capita security standard was 6732 yuan, an increase of 612 yuan over the previous year. At the end of the year, the number of rural minimum living security people was 2.6093 million, and the annual per capita security standard was 3580 yuan, an increase of 396 yuan over the previous year.
At the end of the year, there were 24,095 old-age service institutions and facilities, and 173,600 old-age beds. There are 47 adoption and rescue agencies, and the number of adoption and rescue personnel is 37399. The annual sales of social welfare lottery tickets amounted to 2.823 billion yuan, an increase of 4.9% over the previous year. Social welfare funds raised 884 million yuan, an increase of 3.6%.
XIII. Scientific and Technological Innovation
At the end of the year, there were one national intellectual property demonstration park, two national international scientific and technological cooperation bases, 73 academician workstations and five national key laboratories. 144 scientific and technological achievements at or above the provincial and ministerial levels were made in the whole year, an increase of 20.0% over the previous year. Among them, there were 36 achievements in basic theory, an increase of 28.6% over the previous year, and 108 achievements in applied technology, an increase of 17.4% over the previous year. 2,957 technology contracts were signed, up by 201.7% over the previous year; The turnover was 8.384 billion yuan, an increase of 274.5%.
It has won two National Science and Technology Progress Awards throughout the year. The number of applications for invention patents was 13884, up 26.8% over the previous year. The number of invention patents authorized was 1875, down 7.9%. The number of valid invention patents per 10000 people was 2.37, an increase of 19.1% over the previous year.
The Provincial Governor's Quality Award was established, and 8 enterprises were awarded the Provincial Governor's Quality Award and nominated for the award. One city with strong quality, three national famous brand demonstration zones, 12 provincial famous brand demonstration zones and 692 provincial famous brand products have been established. There are 13 national organic product certification demonstration zones, 1191 organic product certification certificates, 6355 pollution-free agricultural products producing areas and 2850 products. Leading the formulation of 1 international standards for steel cable.
XIV. Education, Culture, Health and Sports
Guizhou University was selected as the national "double first-class" discipline construction university. In the whole year, 836 new and expanded primary and secondary schools, 30 urban compulsory education schools, 900 kindergartens and 112 ordinary high schools were completed.
Postgraduate education enrolled 7,100 students, with 18,600 students enrolled; regular undergraduate and junior college students enrolled 209,500 students, with 627,700 students enrolled; regular senior middle schools enrolled 348,500 students, with 1,011,000 students enrolled; secondary vocational education enrolled 183,100 students, with 503,100 students enrolled; There were 1,829,900 students enrolled in junior middle schools, 650,500 students enrolled in regular primary schools, and 25,800 students enrolled in special education. 738000 children were enrolled in preschool education, and 1534200 children were in kindergartens. The popularization rate of nine-year compulsory education is 90%, and the gross enrollment rate of senior high school is 87.0%.
Figure 12 Number of students enrolled in general colleges, secondary vocational education and general high schools from 2013 to 2017
　　
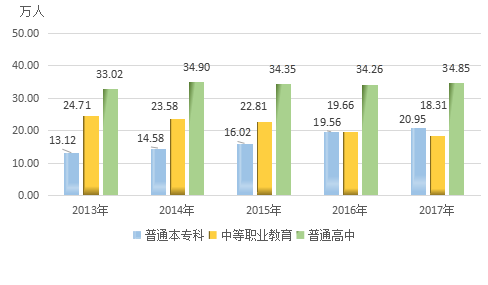

At the end of the year, the comprehensive population coverage rate of radio was 93.5%, and that of television was 96.5%. There are 1.3364 million new users of "Radio and Television Cloud" in Guizhou, and 302 new comprehensive service stations in villages and towns. It has 181 cinemas operating in the city, with a box office revenue of 624 million yuan. It publishes 1050 kinds of books, 39 kinds of newspapers and 93 kinds of magazines. There are 42 art performance groups, 6 art performance venues, 73 museums and memorials, 98 public libraries, 99 mass art galleries and cultural centers, and 1567 comprehensive cultural stations in towns and townships. There are 71 key cultural relics protection units in China.
At the end of the year, there were 28,100 medical and health institutions, including 2,700 hospitals and health centers. There are 362 professional public health institutions, including 100 centers for disease control and prevention. The number of beds in hospitals and health centers was 221,100, an increase of 10.6% over the end of last year.
Athletes won 60 medals in major international and domestic sports competitions throughout the year, an increase of 18 medals over the previous year, an increase of 42.9%. Among them, one world champion and 19 gold medals in the highest level competitions in China were won, an increase of 46.2% over the previous year. There were 117 stadiums and gymnasiums, an increase of 9 or 8.0% over the previous year. 551 sets of national fitness equipment were installed, an increase of 1.7% over the previous year.
Fifteenth, ecological environment and energy saving and consumption reduction
At the end of the year, the forest coverage rate was 55.3%, and the afforestation area was 10 million mu. It has 10 national nature reserves, 30 national forest parks, 44 provincial forest parks and 1 municipal forest park. There are 45 national wetland parks, 4 provincial wetland parks and 2 wetland nature reserves.
The average concentrations of fine particulate matter (PM2.5), inhalable particulate matter (PM10), sulfur dioxide (SO2) and nitrogen dioxide (NO2) in the air of nine central cities in the whole year decreased by 9.4%, 5.7%, 13.3% and 4.5% respectively over the previous year. The proportion of days with good air quality in 9 central cities is 96.5%. The average concentration of chemical oxygen demand (COD) in the provincial control sections of major rivers (151 sections) decreased by 10.4% over the previous year, and the average concentration of ammonia nitrogen decreased by 3.4%. The standard rate of centralized drinking water quality in 9 central cities is 100%.
Renewable energy generated 81.245 billion kWh in the whole year, an increase of 3.3% over the previous year, accounting for 40.4% of the total power generation. Among them, hydropower generation was 73.479 billion kWh, an increase of 1.0%; wind power generation was 6.389 billion kWh, an increase of 20.3%; solar power generation was 750 million kWh, an increase of 456.7%; Biomass and garbage generated 627 million kwh, an increase of 37.8%. The energy consumption of 10000 yuan GDP decreased by 7.0% compared with the previous year, and the energy consumption of 10000 yuan added value of industries above scale decreased by 8.53%. The proportion of the added value of the "four-type" industries in the GDP of the green economy has increased to 37.0%.
Note:
1. The data in this bulletin are preliminary statistics.
2. The growth rate of GDP, industrial added value above scale and its classified items is calculated at comparable prices, which is the actual growth rate; Unless otherwise specified, other indicators are nominal growth rates calculated at current prices.
3. Gross regional product refers to the total value of all final products and services produced by all resident units in a region in a certain period of time.
4. According to the requirements of the Poverty Alleviation Office of the State Council, the population base for calculating the incidence of poverty is the number of agricultural household registration population of the Ministry of Public Security in 2014. In 2017, according to the unified deployment of the dynamic adjustment of the national archives, the province cleared the farmers who did not meet the poverty alleviation standards, supplemented the registration of the poor households who met the poverty alleviation standards, and the population increased or decreased. Therefore, at the end of 2016, 3.722 million poor people and 10.6% of the incidence of poverty were not closed.
5. The statistical scope of investment in fixed assets covers investment in fixed assets projects with a total planned investment of more than 5 million yuan and investment in all real estate development projects. Investment in fixed assets refers to the general term of the workload of construction and purchase of fixed assets completed in a certain period of time in monetary form and the related expenses.
6. The six energy-intensive industries include petroleum processing, coking and nuclear fuel processing, chemical raw materials and chemical products manufacturing, non-metallic mineral products, ferrous metal smelting and calendering, non-ferrous metal smelting and calendering, power and heat production and supply.
7. The statistical scope of industries above designated size is industrial enterprises with annual main business income of 20 million yuan or more.
8. Units above the quota in the statistics of total retail sales of social consumer goods refer to wholesale enterprises (units) with annual main business income of 20 million yuan or more, retail enterprises (units) with annual main business income of 5 million yuan or more, accommodation and catering enterprises (units) with annual main business income of 2 million yuan or more.
9. Online retail sales refer to the sum of retail sales of goods and services through public online trading platforms (including self-built websites and third-party platforms). Goods and services include physical goods and non-physical goods (such as virtual goods, service goods, etc.).
Total retail sales of consumer goods include online retail sales of physical goods, excluding online retail sales of non-physical goods.
10. Consumer price index refers to the relative number reflecting the trend and degree of changes in the prices of consumer goods and services purchased by urban and rural residents in a certain period of time.
11. Due to the rounding of some data, there is a difference between the total and the sub-total.

[[Back to top]](javascript:scroll(0,0)) [[Print this page]](javascript:window.print();) [[Close this page]](javascript:window.close())

Previous:[Statistical Bulletin on the National Economic and Social Development of Guizhou Province in 2017](http://hgk.guizhou.gov.cn/publish/articles/c7/2023/09/a412/a412.html?locationhref=http://hgk.guizhou.gov.cn/publish/channels/c7/c7_1psSuffix&pagesize=15&curpage=1&curainum=14)
Next:[Statistical Communique on the National Economic and Social Development of Guizhou Province in 2016](http://hgk.guizhou.gov.cn/publish/articles/c7/2023/09/a405/a405.html?locationhref=http://hgk.guizhou.gov.cn/publish/channels/c7/c7_1psSuffix&pagesize=15&curpage=2&curainum=1)

Sponsor: Guizhou Provincial Bureau of Statistics Technical Support: Guizhou Jiawang Technology Development Co., Ltd.

Record No.: Qian ICP 19000889 No.: -3

## Statistical Communique on the National Economic and Social Development of Guizhou Province in 2015

## Guizhou Provincial Bureau of Statistics National Bureau of Statistics Guizhou Investigation Team

## (22 March 2016)

## In 2015, facing the complicated economic situation at home and abroad, the whole province thoroughly implemented the spirit of General Secretary 's important speech during his visit to Guizhou, earnestly implemented the decision-making arrangements of the Party Central Committee and the State Council, closely centered on the general requirements of keeping the bottom line, taking a new road and running towards a well-off society, and adhered to accelerating development, accelerating transformation and promoting a new leap forward. In-depth implementation of the main strategy of strengthening the province by industry and urbanization, striving to do a good job in stabilizing growth, promoting reform, adjusting structure, benefiting people's livelihood and preventing risks, successfully completed the main objectives and tasks of economic and social development throughout the year, achieved a successful conclusion of the 12th Five-Year Plan, and laid a solid foundation for the 13th Five-Year Plan to build a well-off society in an all-round way in synchronization with the whole country.

## First, comprehensive

According to the preliminary accounting, in 2015, the gross regional product (GDP) of the whole province exceeded 1 trillion yuan, which was 0.56 billion yuan 10502, an increase of 10.7% over the previous year, and 210.256 billion yuan more than the "Twelfth Five-Year Plan" target (840 billion yuan). During the "Twelfth Five-Year Plan" period, the province's GDP increased by 118 billion 8 million yuan annually, with an average annual growth of 12.5%. The proportion of Guizhou's GDP in the whole country increased from 1.13% in 2010 to 1.55% in 2015.

Of the provincial GDP, the added value of the primary industry was 164.062 billion yuan, up by 6.5% over the previous year; the added value of the secondary industry was 414.694 billion yuan, up by 11.4% over the previous year; The added value of the tertiary industry was 471.5 billion yuan, up by 11.1% over the previous year. The added value of the primary, secondary and tertiary industries accounted for 15.6%, 39.5% and 44.9% of the GDP, respectively. The per capita GDP of the province was 29847 yuan, an increase of 10.3% over the previous year.

| Table 1 GDP of the whole province from 2011 to 2015 | | | | | | |
| --- | --- | --- | --- | --- | --- | --- |
| Unit: 100 million yuan | | | | | | |
| **Indicator name** | **2011** | **2012** | **2013** | **2014** | **2015** | **2015 vs 2014**  **Growth (%)** |
| Gross regional product | 5701.84 | 6852.20 | 8086.86 | 9266.39 | 10502.56 | 10.7 |
| Added value of the primary industry | 726.22 | 891.91 | 998.47 | 1280.45 | 1640.62 | 6.5 |
| Added value of the secondary industry | 2194.33 | 2677.54 | 3276.24 | 3857.44 | 4146.94 | 11.4 |
| Industry | 1829.20 | 2217.06 | 2686.52 | 3140.88 | 3315.58 | 9.8 |
| Construction | 365.13 | 460.48 | 590.69 | 717.69 | 832.55 | 18.3 |
| Added value of the tertiary industry | 2781.29 | 3282.75 | 3812.15 | 4128.50 | 4715.00 | 11.1 |
| Wholesale and retail trade | 448.77 | 514.49 | 582.05 | 624.17 | 671.39 | 8.4 |
| Transportation, storage and postal services | 590.91 | 687.45 | 772.44 | 828.69 | 920.36 | 9.4 |
| Accommodation and catering | 224.40 | 266.58 | 294.86 | 322.71 | 360.38 | 9.7 |
| The financial industry | 297.27 | 365.87 | 444.53 | 491.65 | 607.11 | 19.2 |
| Real estate | 160.30 | 176.75 | 202.94 | 220.48 | 232.07 | 6.6 |
| Other services |  |  |  |  |  |  |
| For-profit service industry | 398.09 | 467.57 | 546.11 | 591.36 | 687.39 | 11.9 |
| Non-profit service industry | 661.55 | 804.04 | 935.02 | 1012.68 | 1163.07 | 11.5 |
| Per capita GDP (yuan) | 16413 | 19710 | 23151 | 26437 | 29847 | 10.3 |
| Note: 1. The data of 2013 is revised according to the data of the third economic census; 2. According to the unified requirements of the National Bureau of Statistics, the national economic accounting data for 2013-2014 adopt the Classification of National Economic Industries (GB/T4754-2011) and the Regulations on the Classification of Three Industries (Guo Tong Zi [2012] No.108). Since 2013, the added value of the primary industry is equal to the sum of the added value of agriculture, forestry, animal husbandry and fishery, excluding the service industry of agriculture, forestry, animal husbandry and fishery; the added value of the secondary industry is equal to the sum of the added value of industry and construction, minus the added value of mining auxiliary activities in industry and the added value of metal product machinery and equipment repair industry; Agriculture, forestry, animal husbandry and fishery services, mining auxiliary activities, metal products, machinery and equipment repair industry are classified into the tertiary industry. | | | | | | |

## II. Agriculture

In 2001, the added value of agriculture, forestry, animal husbandry and fishery was 171.266 billion yuan, up by 6.4% over the previous year. Among them, the added value of planting industry was 109.654 billion yuan, an increase of 7.8%; the added value of forestry was 9.287 billion yuan, an increase of 7.9%; the added value of animal husbandry was 41.594 billion yuan, an increase of 1.2%; the added value of fishery was 3.526 billion yuan, an increase of 17.1%; The added value of agriculture, forestry, animal husbandry and fishery services was 7.204 billion yuan, an increase of 4.6%.

| Table 2 The added value of agriculture, forestry, animal husbandry and fishery in the whole province from 2011 to 2015 | | | | | | |
| --- | --- | --- | --- | --- | --- | --- |
| Unit: 100 million yuan | | | | | | |
| **Indicator name** | **2011** | **2012** | **2013** | **2014** | **2015** | **2015 vs 2014**  **Growth (%)** |
| Added value of agriculture, forestry, animal husbandry and fishery | 726.21 | 891.91 | 1031.70 | 1316.08 | 1712.66 | 6.4 |
| Planting | 430.84 | 561.32 | 646.12 | 851.89 | 1096.54 | 7.8 |
| Forestry | 31.99 | 37.03 | 47.71 | 68.15 | 92.87 | 7.9 |
| Animal husbandry | 223.22 | 245.7 | 280.68 | 331.16 | 415.94 | 1.2 |
| Fishery | 12.78 | 17.83 | 23.96 | 29.25 | 35.26 | 17.1 |
| Agriculture, forestry, animal husbandry and fishery services | 27.38 | 30.03 | 33.23 | 35.63 | 72.04 | 4.6 |

The planting area of grain crops was 3,114.91 thousand hectares, a decrease of 0.8% over the previous year; the planting area of oil crops was 591.85 thousand hectares, an increase of 1.7% over the previous year; the planting area of flue-cured tobacco was 181.67 thousand hectares, a decrease of 16.1% over the previous year; the planting area of vegetables was 996.29 thousand hectares, an increase of 7.8% over the previous year; The planting area of traditional Chinese medicinal materials was 155.81 thousand hectares, an increase of 6.3% over the previous year; the actual area of tea plantations at the end of the year was 418.89 thousand hectares, an increase of 13.5% over the previous year; At the end of the year, the orchard area was 299.89 thousand hectares, an increase of 14.4% over the previous year. The total grain output was 11.8 million tons, an increase of 3.7% over the previous year, the highest level in history, including 2.6945 million tons of summer grain and 9.1055 million tons of autumn grain. The output of tea, fruits, vegetables and traditional Chinese medicines increased by 35.6%, 15.7%, 11.1% and 10.7% respectively over the previous year.

| Table 3 Output of Main Agricultural Products in the Province from 2011 to 2015 | | | | | | |
| --- | --- | --- | --- | --- | --- | --- |
|  |  |  |  |  |  | Unit: 10,000 tons |
| **Indicator name** | **2011** | **2012** | **2013** | **2014** | **2015** | **2015 vs 2014**  **Growth (%)** |
| Food crops | 876.90 | 1079.50 | 1029.99 | 1138.50 | 1180.00 | 3.7 |
| ^#^ Rice | 303.93 | 402.43 | 361.30 | 403.24 | 417.54 | 3.6 |
| Wheat | 50.38 | 52.39 | 51.51 | 61.50 | 61.67 | 0.3 |
| Corn | 243.71 | 342.25 | 298.03 | 313.81 | 324.08 | 3.3 |
| ^#^ Potato | 189.38 | 179.74 | 211.40 | 226.60 | 237.62 | 4.9 |
| Oil crops | 78.85 | 87.38 | 91.53 | 98.05 | 101.55 | 3.6 |
| ^#^ Rapeseed | 71.81 | 78.18 | 81.78 | 86.69 | 89.02 | 2.7 |
| Peanut | 6.07 | 7.86 | 8.25 | 9.71 | 10.71 | 11.3 |
| Flue-cured tobacco | 32.50 | 37.31 | 41.79 | 35.34 | 32.86 | -7.0 |
| Vegetables | 1250.05 | 1375.63 | 1500.45 | 1625.62 | 1805.89 | 11.1 |
| Sugarcane | 43.60 | 127.96 | 159.29 | 168.27 | 156.09 | -7.2 |
| Chinese herbal medicine | 8.36 | 14.06 | 24.80 | 36.06 | 39.91 | 10.7 |
| Tea | 5.84 | 7.44 | 8.94 | 10.71 | 14.53 | 35.6 |
| Fruit | 128.03 | 147.72 | 167.75 | 196.38 | 227.17 | 15.7 |

The annual output of pork was 1,607,500 tons, down by 2.9% over the previous year; the output of beef was 167,600 tons, up by 14.2% over the previous year; the output of mutton was 42,000 tons, up by 12.0% over the previous year; the output of poultry was 163,100 tons, up by 9.9% over the previous year; The output of other meat was 39,200 tons, up by 31.5% over the previous year. Milk output was 62 thousand tons, an increase of 8.6% over the previous year. The output of poultry eggs was 173 thousand and 300 tons, an increase of 7% over the previous year. At the end of the year, 17.9526 million pigs were slaughtered and 15.5896 million pigs were kept in stock, down 2.7% and 2.6% respectively from the previous year. 96.1819 million poultry were sold and 84.0278 million were kept in stock, up 5.0% and 5.9% respectively over the previous year.

| Table 4 Livestock and Poultry Production in the Province from 2011 to 2015 | | | | | | |
| --- | --- | --- | --- | --- | --- | --- |
| **Indicator name** | **2011** | **2012** | **2013** | **2014** | **2015** | **2015 vs 2014**  **Growth (%)** |
| Number of sales in the current year |  |  |  |  |  |  |
| Pigs (10,000) | 1689.66 | 1734.76 | 1832.28 | 1845.27 | 1795.26 | -2.7 |
| Cattle (10,000) | 97.21 | 105.99 | 115.22 | 117.35 | 133.26 | 13.6 |
| Sheep (10,000) | 197.31 | 206.78 | 205.39 | 220.38 | 246.14 | 11.7 |
| Birds (ten thousand feathers) | 8878.57 | 9632.03 | 9681.62 | 9162.14 | 9618.19 | 5.0 |
| Number on hand at the end of the year |  |  |  |  |  |  |
| Pigs (10,000) | 1521.60 | 1604.09 | 1604.10 | 1600.57 | 1558.96 | -2.6 |
| Cattle (10,000) | 467.11 | 461.04 | 460.62 | 495.86 | 535.95 | 8.1 |
| Sheep (10,000) | 256.49 | 290.09 | 299.59 | 337.40 | 354.67 | 5.1 |
| Birds (ten thousand feathers) | 7698.14 | 8355.25 | 8154.72 | 7932.05 | 8402.78 | 5.9 |
| Output of animal products |  |  |  |  |  |  |
| Pork (10,000 tons) | 148.29 | 156.13 | 163.73 | 165.55 | 160.75 | -2.9 |
| Beef (10,000 tons) | 12.00 | 13.04 | 14.13 | 14.68 | 16.76 | 14.2 |
| Mutton (10,000 tons) | 3.37 | 3.53 | 3.51 | 3.75 | 4.20 | 12.0 |
| Poultry meat (10,000 tons) | 14.35 | 15.41 | 15.48 | 14.84 | 16.31 | 9.9 |
| Milk (10,000 tons) | 4.85 | 5.10 | 5.45 | 5.71 | 6.20 | 8.6 |
| Eggs (10,000 tons) | 13.65 | 14.65 | 15.44 | 16.20 | 17.33 | 7.0 |
| Honey (tons) | 2029 | 2052 | 2468 | 2733 | 3017 | 10.4 |
|  | | | | | | |

## III. Industry and Construction

The added value of industries above designated size in the whole year was 355.013 billion yuan, an increase of 9.9% over the previous year. Among them, the added value of light and heavy industries was 137 billion 419 million yuan and 217 billion 594 million yuan respectively, increasing by 8.3% and 10.9% respectively. During the "Twelfth Five-Year Plan" period, the added value of industries above the provincial scale increased by 14.3% annually.

In the whole year, the four traditional industries of coal, electricity, tobacco and alcohol realized an added value of 206.907 billion yuan, accounting for 58.3% of the industrial added value above the scale. Among them, the added value of wine, beverage and refined tea manufacturing industry was 71.605 billion yuan, an increase of 10.2% over the previous year; the added value of coal mining and washing industry was 68.468 billion yuan, an increase of 5.6%; the added value of power, heat production and supply industry was 36.453 billion yuan, an increase of 4.2%. The added value of pharmaceutical manufacturing, computer, communication and other electronic equipment manufacturing exceeded 10 billion yuan and 5 billion yuan respectively, up 6.9% and 102.0% respectively over the previous year. The industrial added value of equipment manufacturing industry and high-tech industry increased by 24.0% and 22.5% respectively over the previous year.

| Table 5 The added value of industries above designated size of the province from 2011 to 2015 | | | | | | |
| --- | --- | --- | --- | --- | --- | --- |
| Unit: 100 million yuan | | | | | | |
| **Indicator name** | **2011** | **2012** | **2013** | **2014** | **2015** | **2015 vs 2014**  **Growth (%)** |
| Industrial added value above designated size | 1638.71 | 2055.46 | 2531.92 | 3117.60 | 3550.13 | 9.9 |
| # Coal mining and washing industry | 411.35 | 472.21 | 558.35 | 676.28 | 684.68 | 5.6 |
| Wine, beverage and refined tea manufacturing | 238.88 | 378.82 | 495.62 | 613.85 | 716.05 | 10.2 |
| Tobacco products industry | 175.81 | 246.89 | 274.74 | 302.19 | 303.81 | -2.3 |
| Chemical raw materials and chemical products manufacturing industry | 96.68 | 109.43 | 124.84 | 141.46 | 159.75 | 9.4 |
| Pharmaceutical manufacturing | 52.02 | 52.70 | 68.45 | 85.53 | 101.63 | 6.9 |
| Non-metallic mineral products industry | 61.41 | 66.34 | 106.79 | 190.56 | 248.63 | 13.0 |
| Ferrous metal smelting and calendering industry | 66.70 | 58.92 | 64.19 | 79.42 | 79.11 | 5.4 |
| Non-ferrous metal smelting and calendering industry | 57.68 | 75.20 | 94.07 | 140.13 | 151.11 | 18.4 |
| Computer, communication and other electronic equipment manufacturing | 10.50 | 14.52 | 20.93 | 20.73 | 52.51 | 102.0 |
| Production and supply of electricity and heat | 234.89 | 295.39 | 318.03 | 340.17 | 364.53 | 4.2 |

In the whole year, 432 new enterprises (excluding growth enterprises) were included in the scope of industrial statistics above the scale, of which 417 were non-public holding industrial enterprises, accounting for 96.5% of the new enterprises included in the scope of industrial statistics above the scale. From the perspective of product distribution, industrial enterprises above the provincial scale produce 282 kinds of industrial products within the statistical scope of 567 kinds, with a product coverage rate of 49.7%.

| Table 6 Output of Major Industrial Products above Designated Size of the Province from 2011 to 2015 | | | | | | |
| --- | --- | --- | --- | --- | --- | --- |
| **Indicator name** | **2011** | **2012** | **2013** | **2014** | **2015** | **2015 vs 2014**  **Growth (%)** |
| Power generation (100 million kWh) | 1359.01 | 1548.44 | 1620.08 | 1682.27 | 1740.92 | 3.3 |
| Phosphate rock (containing 30% of phosphorus pentoxide) (10,000 tons) | 2084.18 | 2281.95 | 2905.44 | 3397.42 | 4323.10 | 15.1 |
| Beverage wine (thousands of liters) | 61.80 | 67.14 | 91.21 | 116.99 | 139.81 | 15.7 |
| # White wine | 25.49 | 26.83 | 30.49 | 38.05 | 42.79 | 11.1 |
| Cigarettes (10,000 cartons) | 245.23 | 249.35 | 254.29 | 258.36 | 252.34 | -2.3 |
| Chinese patent medicine (10,000 tons) | 4.92 | 5.74 | 7.92 | 7.68 | 8.53 | 10.8 |
| Multicolor printed matter (ten thousand folio color order) | 129.57 | 401.14 | 415.62 | 1231.89 | 1469.03 | 19.1 |
| Coke (10,000 tons) | 624.25 | 754.54 | 827.41 | 733.21 | 729.46 | -4.2 |
| Agricultural nitrogen, phosphorus and potassium chemical fertilizers (converted into pure) (10,000 tons) | 360.37 | 503.82 | 524.26 | 533.52 | 582.47 | 8.2 |
| Rubber tire casing (10,000 pieces) | 536.77 | 622.78 | 602.15 | 546.46 | 484.78 | -11.3 |
| Cement (10,000 tons) | 5250.89 | 6100.45 | 8352.95 | 9386.89 | 9909.52 | 5.4 |
| Pig iron (10,000 tons) | 482.35 | 552.93 | 539.22 | 498.64 | 407.58 | -18.2 |
| Steel (10,000 tons) | 462.77 | 560.22 | 573.28 | 552.39 | 463.04 | -16.2 |
| Ferroalloy (10,000 tons) | 263.90 | 309.68 | 327.25 | 348.18 | 332.61 | -4.4 |
| Ten kinds of non-ferrous metals (10,000 tons) | 94.55 | 110.61 | 119.87 | 71.89 | 91.41 | 27.1 |
| Primary aluminum (electrolytic aluminum) (10,000 tons) | 90.36 | 104.35 | 112.28 | 65.21 | 85.52 | 31.1 |
| Household refrigerators (10,000 units) | 169.54 | 159.04 | 155.33 | 168.22 | 174.14 | 3.5 |
| Integrated circuit (10,000 pieces) | 1189.82 | 1558.83 | 1812.71 | 1991.79 | 3079.68 | 51.2 |
| Color TV sets (10,000 sets) | 77.87 | 90.61 | 121.97 | 115.68 | 138.64 | 14.5 |

In 2001, the main business income of industrial enterprises above designated size was 937.620 billion yuan, up by 11.4% over the previous year; The total profit was 61.610 billion yuan, an increase of 10.7% over the previous year.

At the end of the year, there were 892 qualified general contracting enterprises and specialized contracting enterprises in the construction industry, an increase of 6.1% over the end of the previous year. Among them, there are 59 enterprises with Grade I qualification, an increase of 2; and 266 enterprises with Grade II qualification, an increase of 33. In 2001, the total output value of the construction industry was 194.774 billion yuan, up by 18.7% over the previous year. The main business income of construction enterprises was 181.544 billion yuan, an increase of 19.0% over the previous year, and the total profit was 3.908 billion yuan, an increase of 11.4% over the previous year. The total tax was 7.521 billion yuan, an increase of 19.1% over the previous year.

## IV. Investment in fixed assets

In 2001, the 10676 of investment in fixed assets was 0.70 billion yuan, up by 21.6% over the previous year. During the Twelfth Five-Year Plan period, the province completed a total of 330 million yuan of 36089 in fixed assets investment, with an average annual growth of 29.5% from 2012 to 2015.

The investment in infrastructure in the whole year was 413.735 billion yuan, an increase of 22.3% over the previous year, accounting for 38.8% of the province's fixed assets investment. Industrial investment was 274.622 billion yuan, an increase of 17.5% over the previous year, accounting for 25.7% of the province's fixed assets investment. Among them, the manufacturing industry invested 179 billion 567 million yuan, an increase of 18.4%.

| Table 7 Proportion of investment in three major areas in fixed assets investment in the province from 2011 to 2015 | | | | | | |
| --- | --- | --- | --- | --- | --- | --- |
| Unit: 100 million yuan | | | | | | |
| **Year** | **Infrastructure investment** | | **Industrial investment** | | **Investment in real estate development** | |
|  | **Absolute number (100 million yuan)** | **Proportion to investment in fixed assets (%)** | **Absolute number (100 million yuan)** | **Proportion to investment in fixed assets (%)** | **Absolute number (100 million yuan)** | **Proportion to investment in fixed assets (%)** |
| 2011 | 1530.60 | 38.0 | 1332.81 | 33.1 | 873.48 | 21.7 |
| 2012 | 1969.42 | 35.8 | 1614.00 | 29.3 | 1467.60 | 26.7 |
| 2013 | 2587.55 | 36.4 | 1950.07 | 27.5 | 1942.54 | 27.3 |
| 2014 | 3382.19 | 38.5 | 2337.81 | 26.6 | 2187.67 | 24.9 |
| 2015 | 4137.35 | 38.8 | 2746.22 | 25.7 | 2205.09 | 20.7 |

The annual investment in real estate development was 220.509 billion yuan, an increase of 0.8% over the previous year, accounting for 20.7% of the province's fixed assets investment. Housing construction area 20877 67 thousand square meters, an increase of 2.5% over the previous year. Among them, the residential construction area was 13592 65 thousand square meters, down 1.5%. The area of land acquisition was 6.012 million square meters, down 35.8% from the previous year. Commercial housing sales area of 3559.81 square meters, an increase of 12.0% over the previous year; The sales volume of commercial houses was 157.168 billion yuan, an increase of 14.7% over the previous year.

## V. Market and Price

Retail sales of consumer goods totaled 328.302 billion yuan, up by 11.8% over the previous year. According to the location of business units, the retail sales of consumer goods in cities and towns was 269 billion 166 million yuan, an increase of 11.8%. Retail sales of rural consumer goods was 59.136 billion yuan, an increase of 11.7%. During the "Twelfth Five-Year Plan" period, the total retail sales of social consumer goods in the province increased by 17.2% annually.

| Table 8 Total Retail Sales of Social Consumer Goods in the Province from 2011 to 2015 | | | | | | |
| --- | --- | --- | --- | --- | --- | --- |
|  |  |  |  |  | Unit: 100 million yuan | |
| **Indicator name** | **2011** | **2012** | **2013** | **2014** | **2015** | **2015 vs 2014**  **Growth (%)** |
| Total retail sales of consumer goods | 1899.92 | 2266.27 | 2601.20 | 2936.85 | 3283.02 | 11.8 |
| By location of business unit |  |  |  |  |  |  |
| Towns | 1561.07 | 1863.59 | 2148.26 | 2425.25 | 2691.66 | 11.8 |
| Rural | 338.85 | 402.68 | 452.94 | 511.60 | 591.36 | 11.7 |
| By type of consumption |  |  |  |  |  |  |
| Food and beverage income | 205.59 | 233.14 | 245.96 | 266.13 | 300.33 | 12.8 |
| Merchandise retail | 1694.33 | 2033.13 | 2355.24 | 2670.72 | 2982.69 | 11.7 |
| # Retail sales of commodities by units above designated size | 716.24 | 925.83 | 1160.29 | 1461.75 | 1736.76 | 10.0 |
| # Cereals, oils and foodstuffs | 26.98 | 34.47 | 50.38 | 78.16 | 103.77 | 19.4 |
| Alcohol and tobacco | 40.40 | 72.65 | 75.50 | 90.32 | 115.12 | 13.0 |
| Clothing, shoes and hats, knitted textiles | 46.38 | 50.19 | 58.66 | 68.07 | 75.87 | 5.0 |
| Cosmetics | 5.84 | 6.44 | 10.12 | 13.42 | 14.32 | 2.9 |
| Gold, silver and jewelry | 6.25 | 7.34 | 9.51 | 9.60 | 10.40 | -2.2 |
| Daily necessities | 13.28 | 13.75 | 17.40 | 25.83 | 31.48 | 4.9 |
| Sports and entertainment | 1.73 | 1.73 | 2.67 | 2.99 | 3.12 | -2.3 |
| Books, newspapers and magazines | 7.98 | 8.93 | 9.36 | 11.77 | 13.68 | 6.7 |
| Household appliances and audio equipment | 31.13 | 36.13 | 50.34 | 57.53 | 61.03 | 4.0 |
| Chinese and Western medicines | 10.96 | 24.59 | 41.15 | 55.23 | 73.63 | 16.1 |
| Cultural office supplies | 6.92 | 7.23 | 7.98 | 10.12 | 11.63 | 13.7 |
| Furniture | 0.24 | 0.46 | 0.49 | 2.13 | 3.32 | 2.8 |
| Communication equipment | 4.83 | 3.98 | 4.47 | 4.45 | 7.32 | 22.1 |
| Petroleum and its products | 280.13 | 367.10 | 430.30 | 503.31 | 538.90 | 4.1 |
| Construction and decoration materials | 1.64 | 0.80 | 2.06 | 3.20 | 6.25 | 19.5 |
| Cars | 201.35 | 267.75 | 346.02 | 452.85 | 562.87 | 12.6 |

Among the retail sales of commodities above designated size, communication equipment, construction and decoration materials, grain and oil, food, Chinese and Western medicines, tobacco and alcohol, and automobiles increased by 22.1%, 19.5%, 19.4%, 16.1%, 13.0% and 12.6% respectively over the previous year. Enterprises above designated size sold 47.674 billion yuan of commodities through public networks, an increase of 94.4% over the previous year.

Consumer prices for the whole year rose by 1.8% over the previous year. By category, food rose by 2.6%, tobacco and alcohol by 3.2%, clothing by 0.8%, household equipment and services by 1.3%, medical care and personal goods by 0.7%, transportation and communications by 0.6%, entertainment, education, cultural goods and services by 3.4%, and housing by 0.6%. The ex-factory price of industrial producers dropped by 3.9% and the purchasing price of industrial producers dropped by 2.5% over the previous year.

| Table 9 Price Index of the Province from 2011 to 2015 | | | | | |
| --- | --- | --- | --- | --- | --- |
|  |  |  |  | Previous year = 100 | |
| **Indicator name** | **2011** | **2012** | **2013** | **2014** | **2015** |
| Consumer Price Index | 105.1 | 102.7 | 102.5 | 102.4 | 101.8 |
| Food | 113.5 | 104.7 | 104.1 | 104.2 | 102.6 |
| # Food | 116.8 | 104.5 | 103.3 | 102.6 | 102.6 |
| Meat and poultry and their products | 124.5 | 101.3 | 103.8 | 101.3 | 106.6 |
| Aquatic products | 111.1 | 107.6 | 103.1 | 103.0 | 101.7 |
| Eggs | 114.7 | 95.7 | 104.7 | 104.6 | 99.6 |
| Fresh vegetables | 108.3 | 110.8 | 101.3 | 104.5 | 101.5 |
| Dried and fresh melon and fruit | 112.0 | 100.0 | 104.4 | 116.2 | 96.6 |
| Tobacco and alcohol | 102.4 | 102.8 | 101.5 | 99.8 | 103.2 |
| Clothes | 100.3 | 103.8 | 102.3 | 102.1 | 100.8 |
| Household equipment, supplies and services | 100.2 | 101.1 | 101.1 | 100.7 | 101.3 |
| Medical care and personal products | 102.6 | 102.5 | 101.5 | 101.6 | 100.7 |
| Transportation and communications | 100.1 | 99.6 | 99.6 | 100.2 | 100.6 |
| Entertainment, education, cultural goods and services | 99.9 | 101.2 | 101.8 | 102.4 | 103.4 |
| Live | 102.8 | 101.4 | 103.0 | 101.8 | 100.6 |
| Producer Price Index | 105.4 | 101.0 | 97.4 | 98.3 | 96.1 |
| Purchasing price index of industrial producers | 115.0 | 102.3 | 96.4 | 98.6 | 97.5 |
| Price index of investment in fixed assets | 105.4 | 101.5 | 100.9 | 101.1 | 98.4 |
| Price index of construction and installation works | 107.5 | 102.0 | 101.5 | 101.3 | 98.1 |

## VI. Foreign Economy

The total import and export volume of the year was 76.122 billion yuan, an increase of 14.9% over the previous year. The total import volume was 14.266 billion yuan, an increase of 69.0% over the previous year, of which the import of general trade was 4.885 billion yuan, a decrease of 27.5%, and the import of processing trade was 3.033 billion yuan, an increase of 187.1%. The total export volume was 61.856 billion yuan, an increase of 7.0% over the previous year, of which the export of general trade was 54.258 billion yuan, a decrease of 1.4%. The export of processing trade was 4.140 billion yuan, an increase of 143.4%.

In the whole year, the actual capital in place outside the province was 721.351 billion yuan, an increase of 20.1% over the previous year. 4322 projects were introduced from outside the province, an increase of 84.6% over the previous year. The 11912 of investment in the imported projects agreed upon in the contracts was 1.44 billion yuan, an increase of 58.4% over the previous year. 187 foreign investment projects were newly approved, up 8.7% over the previous year.

## VII. Transportation, Posts and Telecommunications, and Tourism

At the end of the year, the mileage of highways open to traffic in the whole province was 183812 kilometers, an increase of 2.6% over the end of the previous year, of which the mileage of expressways open to traffic was 5128 kilometers, and the number of expressways out of the province increased to 15. The railway mileage is 3037 kilometers, of which the high-speed railway mileage is 701 kilometers, and the number of railway corridors out of the province has increased to 12. Fifty-six new encrypted routes have been started, and the number of navigable cities of Guiyang Airport has increased to 81. The length of inland waterways is 3661 kilometers, and the length of high-grade waterways is 690 kilometers.

The freight turnover volume of railway, highway and waterway in the whole year was 45.825 billion ton kilometers, 89.710 billion ton kilometers and 3.715 billion ton kilometers respectively, accounting for 32.9%, 64.4% and 2.7% of the freight turnover volume of the whole province respectively. The passenger turnover of railway, highway and waterway was 20.754 billion person-kilometers, 44.042 billion person-kilometers and 552 million person-kilometers respectively, accounting for 31.8%, 67.4% and 0.8% of the total passenger turnover of the province. Civil aviation handled 89,600 tons of cargo and mail, up by 7.9% over the previous year; Civil aviation handled 15.6328 million passengers, an increase of 10.0% over the previous year.

| Table 10 Freight and Passenger Transport Volume of the Province from 2011 to 2015 | | | | | | |
| --- | --- | --- | --- | --- | --- | --- |
| **Indicator name** | **2011** | **2012** | **2013** | **2014** | **2015** | **Increase from 2014 to 2015 (%)** |
|
| Cargo turnover (100 million ton-kilometers) | 1060.69 | 1177.78 | 1292.11 | 1442.24 | 1392.51 | 5.0 |
| Railway | 696.36 | 693.68 | 655.85 | 634.35 | 458.25 | -11.6 |
| Highway | 350.10 | 467.60 | 610.64 | 776.95 | 897.10 | 15.5 |
| Water transport | 14.23 | 16.50 | 25.62 | 30.94 | 37.15 | 20.1 |
| Cargo and mail throughput of civil aviation (10,000 tons) | 6.93 | 7.97 | 7.76 | 8.31 | 8.96 | 7.9 |
| Passenger turnover (100 million person-km) | 631.74 | 718.22 | 593.62 | 635.50 | 653.48 | 8.1 |
| Railway | 204.58 | 199.20 | 211.21 | 217.39 | 207.54 | 11.2 |
| Highway | 422.01 | 513.07 | 377.87 | 412.92 | 440.42 | 6.7 |
| Water transport | 5.15 | 5.95 | 4.54 | 5.19 | 5.52 | 6.4 |
| Civil aviation passenger throughput (10,000 person-times) | 747.02 | 890.99 | 1125.46 | 1420.68 | 1563.28 | 10.0 |

In 2001, the business volume of posts and telecommunications was 51.487 billion yuan, up by 34.9% over the previous year. Of this, the volume of telecommunications business was 48.110 billion yuan, up 35.9%, and the volume of postal business was 3.377 billion yuan, up 21.7%. The total volume of express business was 70.3425 million pieces, an increase of 50.7% over the previous year; the revenue of express business was 1.324 billion yuan, an increase of 34.9% over the previous year. At the end of the year, there were 34.8485 million telephone users in the whole province, including 31.7231 million mobile phone users, 101 telephone penetration rate/100 people; there were 3.913 million fixed Internet users, the length of optical cable lines exceeded 600,000 km, and 9.167 million Internet broadband access ports. There are 163,000 mobile communication base stations.

The total number of tourists in the whole year was 376 million, an increase of 17.1% over the previous year. Among them, 375 million domestic tourists were received, an increase of 17.1%; The number of inbound tourists was 940900, an increase of 10.0%. The total tourism revenue reached 351.282 billion yuan, an increase of 21.3% over the previous year.

| Table 11 Total number and total income of tourism industry in the province from 2011 to 2015 | | | | | |
| --- | --- | --- | --- | --- | --- |
| **Indicator name** | **2011** | **2012** | **2013** | **2014** | **2015** |
| Total number of tourists (10,000 person-times) | 17019.36 | 21401.18 | 26761.28 | 32134.94 | 37630.01 |
| Total number of tourists increased over the previous year (%) | 31.8 | 25.7 | 25.0 | 20.1 | 17.1 |
| Total tourism revenue (100 million yuan) | 1429.48 | 1860.16 | 2370.65 | 2895.98 | 3512.82 |
| Total tourism revenue increased over the previous year (%) | 34.7 | 30.1 | 27.4 | 22.2 | 21.3 |

## VIII. Finance and Banking

The total fiscal revenue of the year was 229.425 billion yuan, an increase of 7.7% over the previous year. The general public budget revenue was 150.335 billion yuan, an increase of 10.0% over the previous year, of which tax revenue was 112.596 billion yuan, an increase of 9.7%.

| Table 12 Main Fiscal Revenue of the Province from 2011 to 2015 | | | | | | |
| --- | --- | --- | --- | --- | --- | --- |
|  |  |  |  |  |  | Unit: 100 million yuan |
| **Indicator name** | **2011** | **2012** | **2013** | **2014** | **2015** | **Increase from 2014 to 2015 (%)** |
| Total fiscal revenue | 1329.99 | 1644.48 | 1918.23 | 2130.90 | 2294.25 | 7.7 |
| # General public budget revenue | 773.08 | 1014.05 | 1206.41 | 1366.67 | 1503.35 | 10.0 |
| # Domestic VAT (including VAT) | 76.41 | 85.74 | 96.06 | 117.03 | 120.44 | 2.9 |
| Business tax | 181.73 | 241.53 | 300.78 | 344.49 | 353.08 | 2.5 |
| Corporate income tax | 70.69 | 86.53 | 103.15 | 123.84 | 127.31 | 2.8 |
| Personal income tax | 33.24 | 32.29 | 34.65 | 32.52 | 33.27 | 2.3 |
| Urban maintenance and construction tax | 37.82 | 45.79 | 53.71 | 57.64 | 59.80 | 3.7 |
| Deed tax | 23.91 | 33.00 | 41.42 | 66.93 | 73.40 | 9.7 |
| Income from state-owned capital operation | 6.38 | 7.21 | 10.97 | 12.91 | 15.80 | 22.4 |

The annual general public budget expenditure was 393 billion 21 million yuan, an increase of 10.9% over the previous year. Of this total, 77.062 billion yuan was spent on education, up 21.0%; 33.957 billion yuan on social security and employment, up 13.3%; and 35.750 billion yuan on medical and health care and family planning, up 17.9%. Expenditure on agriculture, forestry and water was 53.461 billion yuan, an increase of 19.5%.

| Table 13 Main Financial Expenditures of the Province from 2011 to 2015 | | | | | | |
| --- | --- | --- | --- | --- | --- | --- |
|  |  |  |  |  |  | Unit: 100 million yuan |
| **Indicator name** | **2011** | **2012** | **2013** | **2014** | **2015** | **Increase from 2014 to 2015 (%)** |
| General public budget expenditure | 2249.40 | 2755.68 | 3082.66 | 3542.80 | 3930.21 | 10.9 |
| # Expenditure on general public services | 307.21 | 430.16 | 488.78 | 422.49 | 432.94 | 2.5 |
| Expenditure on education | 376.86 | 500.51 | 560.67 | 637.03 | 770.62 | 21.0 |
| Expenditure on science and technology | 21.68 | 28.98 | 34.27 | 44.34 | 58.19 | 31.2 |
| Expenditure on culture, sports and media | 35.31 | 49.85 | 48.68 | 54.69 | 61.37 | 12.2 |
| Expenditure on social security and employment | 194.78 | 235.40 | 264.52 | 299.72 | 339.57 | 13.3 |
| Expenditure on health care and family planning | 173.26 | 201.05 | 228.71 | 303.25 | 357.50 | 17.9 |
| Expenditure on energy conservation and environmental protection | 55.45 | 65.73 | 66.44 | 85.34 | 95.87 | 12.3 |
| Expenditure on agriculture, forestry and water | 278.47 | 361.87 | 400.31 | 447.19 | 534.61 | 19.5 |
| Transportation expenditure | 305.16 | 288.56 | 299.79 | 432.01 | 392.56 | -9.1 |

At the end of the year, the balance of various kinds of RMB deposits in the financial institutions of the whole province was 19438 1.64 billion yuan, an increase of 26.9% over the end of the previous year. Among them, household deposits were 739.486 billion yuan, accounting for 38.0% of the RMB deposit balance of financial institutions; non-financial enterprise deposits were 679.574 billion yuan, accounting for 35.0%; Broad government deposits amounted to 461 billion 466 million yuan, accounting for 23.7%.

At the end of the year, the balance of RMB loans of financial institutions in the whole province was 1505.194 billion yuan, an increase of 21.7% over the end of last year. Among them, household loans amounted to 442.917 billion yuan, accounting for 29.4% of the balance of RMB loans of financial institutions. Loans from non-financial enterprises and institutions amounted to 106.2093 billion yuan, accounting for 70.6%.

| Table 14 Balance of RMB deposits of financial institutions in the province at the end of 2015 | | |
| --- | --- | --- |
|  |  | Unit: 100 million yuan |
| **Indicator name** | **Absolute number** | **Increase over the beginning of the year** |
| Balance of RMB deposits of financial institutions | 19438.64 | 4128.20 |
| Domestic deposits | 19433.26 | 4128.16 |
| Household deposits | 7394.86 | 631.74 |
| Demand deposit | 3877.60 | 281.12 |
| Time and other deposits | 3517.27 | 350.62 |
| Deposits of non-financial enterprises | 6795.74 | 1670.15 |
| Demand deposit | 4489.54 | 1470.95 |
| Time and other deposits | 2306.20 | 199.20 |
| Government deposits in broad sense | 4614.66 | 1336.28 |
| Fiscal deposits | 761.66 | 227.12 |
| Deposits of organs and organizations | 3853.00 | 1109.16 |
| Deposits of non-banking financial institutions | 627.99 | 489.99 |
| Offshore deposits | 5.38 | 0.04 |

| Table 15 Balance of RMB loans of financial institutions in the province at the end of 2015 | | |
| --- | --- | --- |
|  |  | Unit: 100 million yuan |
| **Indicator name** | **Absolute number** | **Increase over the beginning of the year** |
| Balance of RMB loans of financial institutions | 15051.94 | 2683.42 |
| Domestic loans | 15051.32 | 2683.46 |
| Household loans | 4429.17 | 656.10 |
| Short-term loan | 1073.89 | 291.66 |
| Consumer loans | 285.32 | 94.39 |
| Operating loans | 788.58 | 197.27 |
| Medium and long-term loans | 3355.28 | 364.43 |
| Consumer loans | 2228.82 | 286.00 |
| Operating loans | 1126.46 | 78.44 |
| Loans to non-financial enterprises and institutions | 10620.93 | 2026.37 |
| Short-term loan | 2069.44 | 47.78 |
| Medium and long-term loans | 8324.97 | 1912.02 |
| Bill financing | 196.28 | 61.83 |
| Various advances | 30.23 | 4.94 |
| Loans from non-banking financial institutions | 1.22 | 1.00 |
| Offshore loans | 0.62 | -0.05 |

The original insurance premium income of the whole year was 25.780 billion yuan, an increase of 21.0% over the previous year. Among them, property insurance income was 13.395 billion yuan, an increase of 19.1%; life insurance income was 12.385 billion yuan, an increase of 23.1%. Insurance compensation expenditure was 10.697 billion yuan, an increase of 19.3% over the previous year. Among them, property insurance compensation expenditure was 6 billion 664 million yuan, an increase of 19.1%; Life insurance compensation expenditure was 4.033 billion yuan, an increase of 19.5%.

| Table 16 Development of Insurance Industry in the Province in 2015 | | |
| --- | --- | --- |
| Unit: 100 million yuan | | |
| **Indicator name** | **Absolute number** | **Year-on-year growth (%)** |
| Original premium income | 257.80 | 21.0 |
| # Property insurance | 133.95 | 19.1 |
| # Motor Vehicle Insurance | 110.35 | 18.7 |
| Personal insurance | 123.85 | 23.1 |
| Accident Insurance | 9.90 | 15.6 |
| Health insurance | 16.87 | 28.1 |
| Life Insurance | 97.08 | 23.1 |
| Compensation expenses | 106.97 | 19.3 |
| # Property insurance | 66.64 | 19.1 |
| # Motor Vehicle Insurance | 56.47 | 15.0 |
| Personal insurance | 40.33 | 19.5 |
| Accident Insurance | 3.26 | 0.6 |
| Health insurance | 8.18 | 21.5 |
| Life Insurance | 28.89 | 21.6 |

In 2001, funds raised from the securities market totaled 43.733 billion yuan, up by 12.1% over the previous year. At the end of the year, there were 20 listed companies in the province, down 4.8% from the end of the previous year, with a total capital stock of 17.637 billion yuan, up 39.4% from the end of the previous year. The total market value was 527.930 billion yuan, an increase of 31.9% over the end of last year. Securities and futures investors opened 887 thousand and 300 accounts, an increase of 33.67% over the previous year. The 23592 from securities and futures transactions was 0.39 billion yuan, an increase of 97.1% over the previous year.

## Ix. People's livelihood

The annual per capita disposable income of residents in the province was 13696. 61 yuan, a nominal increase of 10.7% over the previous year. By permanent residence, the per capita disposable income of urban residents and rural residents was 24579 64 yuan and 7386.87 yuan, respectively, an increase of 9.0% and 10.7% over the previous year.

| Table 17 Per capita disposable income of urban and rural residents in 2015 | | | |
| --- | --- | --- | --- |
| Unit: yuan | | | |
| **Indicator name** | **Absolute number** | **Share of disposable income (%)** | **Year-on-year growth (%)** |
| Per capita disposable income of urban residents | 24579.64 |  | 9.0 |
| Wage income | 14166.15 | 57.6 | 7.8 |
| Net income from operations | 3729.81 | 15.2 | 17.6 |
| Net income from property | 1868.13 | 7.6 | 7.0 |
| Net income transferred | 4815.55 | 19.6 | 7.5 |
| Per capita disposable income of rural residents | 7386.87 |  | 10.7 |
| Wage income | 2897.14 | 39.2 | 14.9 |
| Net income from operations | 2878.71 | 39.0 | 8.9 |
| Net income from property | 83.7 | 1.1 | 17.9 |
| Net income transferred | 1527.32 | 20.7 | 6.4 |

The annual per capita consumption expenditure of permanent urban residents was 16914. 20 yuan, an increase of 10.9% over the previous year. The per capita consumption expenditure of rural residents was 6644.93 yuan, an increase of 11.3% over the previous year.

| Table 18 Per capita consumption expenditure of urban and rural residents in 2015 | | | |
| --- | --- | --- | --- |
| Unit: yuan | | | |
| **Indicator name** | **Absolute number** | **As a percentage of consumer spending**  **Proportion of (%)** | **Year-on-year growth (%)** |
| Per capita consumption expenditure of permanent urban residents | 16914.20 |  | 10.9 |
| Food, tobacco and alcohol | 5757.29 | 34.0 | 8.2 |
| Clothes | 1346.73 | 8.0 | 8.1 |
| Live | 2993.81 | 17.7 | 23.1 |
| Daily necessities and services | 1078.55 | 6.4 | -1.1 |
| Health care | 872.24 | 5.2 | -5.4 |
| Traffic and communication | 2248.35 | 13.3 | 20.0 |
| Education, culture and entertainment | 2312.69 | 13.7 | 11.7 |
| Other supplies and services | 304.53 | 1.8 | 3.5 |
| Per capita consumption expenditure of rural residents | 6644.93 |  | 11.3 |
| Food, tobacco and alcohol | 2644.56 | 39.8 | 6.3 |
| Clothes | 355.36 | 5.3 | 4.0 |
| Live | 1355.57 | 20.4 | 12.8 |
| Daily necessities and services | 379.8 | 5.7 | 7.0 |
| Health care | 449.46 | 6.8 | 20.5 |
| Traffic and communication | 784.23 | 11.8 | 23.2 |
| Education, culture and entertainment | 584.75 | 8.8 | 21.5 |
| Other supplies and services | 91.2 | 1.4 | -1.0 |

| Table 19 Number of durable consumer goods owned per 100 urban households in the province in 2015 | | |
| --- | --- | --- |
| **Indicator name** | **Absolute number** | **Year-on-year growth (%)** |
| Water heater (set) | 82.3 | 7.4 |
| Air conditioner (set) | 27.81 | 35.8 |
| Camera (part) | 4.62 | 8.8 |
| Computer (set) | 64.44 | 1.4 |
| Mobile phone (unit) | 236.52 | 3.7 |
| Color TV set | 106.23 | 1.1 |
| Refrigerator (cabinet) | 93.94 | 3.6 |
| Motorcycle (unit) | 17.91 | -14.0 |
| Family car (unit) | 23.74 | 20.7 |

| Table 20 Number of durable consumer goods owned by every 100 permanent rural households in the province in 2015 | | |
| --- | --- | --- |
| **Indicator name** | **Absolute number** | **Year-on-year growth (%)** |
| Washing machine (set) | 77.8 | 3.5 |
| Motorcycle (unit) | 54.9 | 13.4 |
| Color TV set | 101.8 | 2.9 |
| Water heater (set) | 21.6 | 10.2 |
| Fixed telephone (unit) | 8.4 | -36.8 |
| Mobile phone (unit) | 227.3 | 4.2 |
| Refrigerator (cabinet) | 62.4 | 6.4 |

## X. Education, Science and Technology

At the end of the year, there were 26 thousand and 600 schools at all levels in the province, down 3.7% from the end of last year. There were 10.8107 million students in schools at all levels, an increase of 1.5% over the end of the previous year, and 487,200 full-time teachers in schools at all levels, an increase of 5.3% over the end of the previous year. The enrollment rate of primary school-age children was 99.5%, an increase of 0.4 percentage points over the previous year; the gross enrollment rate of junior middle school students was 104.0%, an increase of 1.5 percentage points over the previous year; and the gross enrollment rate of senior middle school students was 86.1%, an increase of 8.1 percentage points over the previous year. The gross enrollment rate of higher education was 31.2%, an increase of 1.8 percentage points over the previous year.

| Table 21 Number of Enrollment, Students and Graduates in the Province in 2015 | | | | | | |
| --- | --- | --- | --- | --- | --- | --- |
| Unit: 10,000 persons | | | | | | |
| **Indicator name** | **Number of students enrolled** | | **Number of students in school** | | **Number of graduates** | |
|  | **Absolute number** | **Compared with the previous year**  **Growth (%)** | **Absolute number** | **Compared with the previous year**  **Growth (%)** | **Absolute number** | **Compared with the previous year**  **Growth (%)** |
| Graduate Education | 0.54 | 6.1 | 1.55 | 5.6 | 0.45 | 3.2 |
| General Higher Education | 16.02 | 9.8 | 50.09 | 8.8 | 11.68 | 17.3 |
| Higher Vocational Colleges | 8.44 | 15.9 | 19.87 | 15.6 | 4.71 | 17.9 |
| Secondary Vocational Education  (School) | 22.81 | -3.3 | 60.25 | 10.7 | 11.88 | 9.5 |
| Ordinary high school | 34.35 | -1.6 | 97.89 | 3.8 | 28.07 | 17.7 |
| Junior high school | 63.27 | -5.2 | 197.97 | -4.3 | 70.24 | 4.2 |
| Ordinary primary school | 57.24 | 3.2 | 346.31 | 0.0 | 63.69 | -4.2 |

In the whole year, 115 scientific and technological achievements at or above the provincial and ministerial levels were registered, down 21.8% from the previous year. Among them, there were 25 basic theoretical achievements, an increase of 13.6%; 89 technical achievements were applied, down by 28.2%. 654 technology contracts were signed, down 0.6% from the previous year, with a turnover of 260 million yuan, an increase of 29.8%. There were 18295 patent applications, down 18.6% from the previous year. The number of authorized patent 14115 increased by 39.7% over the previous year. 545 enterprises completed product certification, an increase of 21.1% over the previous year.

| Table 22 Main Situation of Science and Technology Development in the Province in 2015 | | |
| --- | --- | --- |
| **Indicator name** | **Absolute number** | **Year-on-year growth (%)** |
| Registration of scientific and technological achievements at or above the provincial and ministerial levels (items) | 115 | -21.8 |
| # Achievements in basic theory | 25 | 13.6 |
| Application of technical achievements | 89 | -28.2 |
| Soft science achievements | 1 | Flat |
| Sign technical contract (item) | 654 | -0.6 |
| Transaction amount (ten thousand yuan) | 260190 | 29.8 |
| Professional and technical personnel of public economic enterprises and institutions (10,000 persons) | 66.6 | 3.7 |
| # Personnel with intermediate and above professional and technical titles | 29.47 | 3.7 |
| Ethnic minorities | 26.04 | 3.7 |
| Female | 30.73 | 3.7 |
| Number of intermediate and above skilled personnel in the province (10,000 persons) | 33.92 | 5.0 |
| Patent application (piece) | 18295 | -18.6 |
| Authorized patent (piece) | 14115 | 39.7 |
| Product quality supervision organization (unit) | 48 | -14.3 |
| # National Product Quality Supervision and Inspection Center | 4 | 100 |
| Product quality and system certification organization (unit) | 1 | Flat |
| Enterprises that have completed product certification (units) | 545 | 21.1 |
| Legal metrological technical institutions (unit) | 113 | Flat |
| Compulsory verification of measuring instruments (10,000 sets) | 41.81 | 39.5 |

## Xi. Culture, Health and Sports

At the end of the year, there were 41 art performance groups, 98 mass art galleries and cultural centers, 96 public libraries, 107 archives, 74 museums and memorials, 6 art performance venues and 1565 comprehensive cultural stations in towns and townships. The number of books published in the whole year is 10361, and the number of magazines published is 17.5098 million copies, and the number of newspapers published is 322 million copies. At the end of the year, the comprehensive population coverage rate of radio was 92.3%, and that of television was 96.0%.

At the end of the year, there were 28,700 health institutions in the province. Among them, there were 0.26 million hospitals and health centers, an increase of 4.0% over the end of last year; There were 102 maternal and child health care centers (institutes and stations), an increase of 2.0%. The number of beds in health institutions was 197 thousand and 100, an increase of 8.5% over the end of last year. There were 186900 health technicians, an increase of 10.1% over the end of last year. Among them, there were 63,400 practicing (assistant) doctors, an increase of 9.7%, and 76,000 registered nurses, an increase of 13.3%. There were 32.9233 million farmers participating in the new rural cooperative medical system, with a participation rate of 99.12%, an increase of 0.22 percentage points over the previous year.

Athletes won 41 awards in major international and domestic sports competitions throughout the year, an increase of 17.1% over the previous year. At the end of the year, there were 59 stadiums in the province.

## XII. Ecology, Environment and Safety in Production

The afforestation area in the whole year was 280000 hectares, and the forest coverage rate at the end of the year was 50.0%, an increase of 1.0 percentage points over the previous year. The water quality of centralized drinking water sources in the central cities of nine cities (prefectures) reached the standard rate of 100%, the excellent rate of air quality index was higher than 90%, and the number of days with good air quality in cities above county level exceeded 95%. At the end of the year, 628 provincial ecological civilization construction demonstration zones were approved, an increase of 66.1% over the end of last year. There are 123 nature reserves, including 9 national nature reserves; the area of nature reserves accounts for 5.6% of the total land area of the province.

The annual investment in environmental protection was 9.574 billion yuan, an increase of 5.0% over the previous year. The treatment capacity of sewage treatment plants in county towns and above was 2.4658 million cubic meters per day, an increase of 28.8% over the previous year, and the sewage treatment rate reached 89.3%, an increase of 2.0 percentage points over the previous year. The 33060 of green area in urban built-up areas was 0.77 hectares, an increase of 12.7% over the previous year, and the green rate of built-up areas was 23.1%, an increase of 1.7 percentage points over the previous year. The harmless treatment rate of municipal solid waste was 82.8%, the comprehensive utilization rate of industrial solid waste was 58.0%, and the industrial reuse rate was 95.0%, which increased by 3.9, 1.1 and 0.8 percentage points respectively over the previous year. The energy consumption of 10000 yuan GDP decreased by 7.46% compared with the previous year.

A total of 1,142 production safety accidents occurred in the whole year, a decrease of 10.2% over the previous year, and 877 people died, a decrease of 10.6%. Among them, 1035 road traffic accidents occurred, down 9.6%; There were 741 deaths, down 6.8%.

## XIII. Population, employment and social security

At the end of the year, the permanent population of the province was 35.295 million. By urban and rural areas, the urban population is 14.8274 million, and the rural population is 20.4676 million. The urban population accounted for 42.01% of the permanent population at the end of the year, an increase of 2 percentage points over the previous year. By gender, the male population is 18.2063 million and the female population is 17.0887 million. The birth rate of the whole province was 13.00 per thousand, an increase of 0.02 per thousand over the previous year, and the death rate was 7.20 per thousand, an increase of 0.02 per thousand over the previous year. The natural population growth rate was 5.80 per thousand, the same as the previous year.

726800 new jobs were created in cities and towns throughout the year, an increase of 6.3% over the previous year. Among them, 145 thousand and 500 unemployed people were re-employed, down 4.8% from the previous year. At the end of the year, the number of registered unemployed people in cities and towns was 144900, and the registered unemployment rate in cities and towns was 3.29%.

At the end of the year, 3.9209 million people in the province participated in the basic old-age insurance for urban employees, an increase of 8.5% over the end of the previous year, of which 2.8801 million were enterprise employees, an increase of 7.3%; 16.4903 million people participated in the basic old-age insurance for urban and rural residents, an increase of 3.9% over the end of the previous year; The number of people participating in unemployment insurance was 2.0531 million, an increase of 7.0% over the end of the previous year; the number of people participating in industrial injury insurance was 2.9028 million, an increase of 5.4% over the end of the previous year; The number of people participating in maternity insurance was 2.6367 million, an increase of 6.0% over the end of last year. We implemented 502,200 government-subsidized housing units in cities and towns, completed 225600 government-subsidized housing units in cities and towns, and renovated 350000 dilapidated houses in rural areas.

At the end of the year, a total of 402800 people in the province enjoyed the minimum living security for urban residents, down 15.4% from the end of the previous year. 3.3148 million people enjoyed the minimum living security for rural residents, down 20.5% from the end of last year. All kinds of social service institutions providing accommodation adopted and assisted 27800 people, of which 23600 were adopted and assisted by old-age service institutions. The province sold 2.498 billion yuan of social welfare lottery tickets, an increase of 16.1% over the previous year. It raised 78100 yuan of social welfare funds, an increase of 12.1%.

Note:

[1] Data for 2015 are preliminary statistics, and data for 2014 and previous years are annual reports. Due to rounding, some data are not equal to the total of sub-items. "#" indicates the item.

[2] The absolute figures of GDP, industrial added value and per capita GDP are calculated at current prices, and the growth rate is calculated at comparable prices.

[3] The statistical caliber of industries above designated size is industrial enterprises with annual main business income of 20 million yuan or more. The statistical caliber of fixed assets investment is the investment in fixed assets projects and real estate development projects with a total planned investment of 5 million yuan or more. The statistical caliber of the wholesale industry above the designated size is the wholesale enterprises and individual households with annual main business income of 20 million yuan or more, the statistical caliber of the retail industry above the designated size is the retail enterprises and individual households with annual main business income of 5 million yuan or more, and the statistical caliber of the accommodation and catering industry above the designated size is the accommodation and catering enterprises and individual households with annual main business income of 2 million yuan or more.

[4] According to the provisions of the national statistical system, the statistical caliber of fixed assets investment projects has been increased from 500,000 yuan to 5 million yuan since 2011. Therefore, the average annual growth rate of fixed assets investment in the same caliber during the 12th Five-Year Plan period can only be calculated from 2012 to 2015.

Current location:[Home page](http://hgk.guizhou.gov.cn/index.vhtml) > [Statistical publications](http://hgk.guizhou.gov.cn/publish/channels/c6/c6_1.html)> [Statistical Bulletin](http://hgk.guizhou.gov.cn/publish/channels/c7/c7_1.html)> [Statistical Communique on the National Economic and Social Development of Guizhou Province in 2022](http://hgk.guizhou.gov.cn/publish/articles/c7/2023/05/a1016/a1016.html)

Statistical Bulletin

Statistical Communique on the National Economic and Social Development of Guizhou Province in 2022

Date: 2023-05-17 Article Source: Provincial Bureau of Statistics No.: [[Big](javascript:doZoom(20)) [Medium](javascript:doZoom(16)) [Small](javascript:doZoom(12)) ]

Guizhou Provincial Bureau of Statistics National Bureau of Statistics Guizhou Investigation Team

(17 May 2023)

2022 is a very unusual year in the history of Guizhou's development. The whole province has thoroughly implemented the spirit of the 20th National Congress of the CPC and the spirit of General Secretary 's important speech on inspecting Guizhou, comprehensively implemented the important requirements of "epidemic prevention, economic stability and development security", adhered to the overall situation of high-quality development, coordinated epidemic prevention and control and economic and social development, coordinated development and security, and made every effort to focus on the "four new". Efforts should be made to build "four districts and one highland", and every effort should be made to solve multiple constraints and difficult problems, so as to make new achievements in promoting high-quality development.

First, comprehensive

According to the unified accounting results of GDP, the province's GDP in 2016 was 4.58 billion yuan, an increase of 1.2% over the previous year. Among them, the added value of the primary industry was 286.118 billion yuan, an increase of 3.6%; the added value of the secondary industry was 711.303 billion yuan, an increase of 0.5%; the added value of the tertiary industry was 10190 1.37 billion yuan, an increase of 1.0%. The added value of the primary industry accounted for 14.2% of the GDP of the region, an increase of 0.2 percentage points over the previous year; the added value of the secondary industry accounted for 35.3% of the GDP of the region, an increase of 0.1 percentage points over the previous year; The added value of the tertiary industry accounted for 50.5% of the GDP, down 0.3 percentage points from the previous year. Per capita GDP was 52321 yuan, an increase of 1.2% over the previous year. The total labor productivity was yuan 103797 per person, an increase of 1.6% over the previous year.


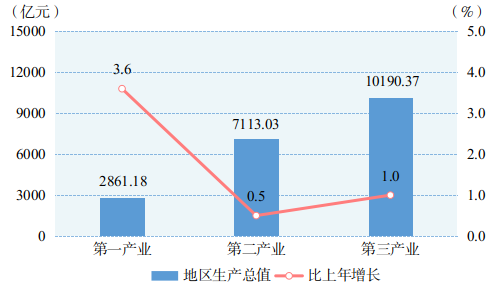


**Figure 1 The added value of the three industries and their growth rate**

At the end of the year, the permanent population of the province was 38.56 million, an increase of 40000 over the end of the previous year. Among them, the urban permanent population was 21.14 million, accounting for 54.81% of the permanent population at the end of the year, an increase of 0.48 percentage points over the end of last year. There were 425,000 births in the whole year, with a birth rate of 11.03 per thousand, and 282,000 deaths, with a death rate of 7.32 per thousand. The natural growth rate was 3.71 per thousand.

**Table 1 Number and Composition of Resident Population at the End of 2022**

| **Indicator name** | **Absolute number (10,000 persons)** | **As a percentage of the resident population at the end of the year**  **Specific gravity (%)** |
| --- | --- | --- |
| **Resident population at the end of the year** | 3856 | 100 |
| **By urban and rural areas** |  |  |
| **Towns** | 2114 | 54.81 |
| **Rural** | 1742 | 45.19 |
| **By gender** |  |  |
| **Male** | 1971 | 51.12 |
| **Female** | 1885 | 48.88 |
| **By age** |  |  |
| **0-15 years old (including less than 16 years old)** | 947 | 24.56 |
| **16-59 years old (including less than 60 years old)** | 2307 | 59.83 |
| **60 years old and above** | 602 | 15.61 |
| **# 65 years and above** | 468 | 12.14 |

At the end of the year, 18.78 million people were employed in the province, of which 9.81 million were employed in cities and towns, accounting for 52.2% of the province's total employment. In the whole year, 609200 new jobs were created in cities and towns, down 5.9% from the previous year. Among them, 151 thousand and 600 unemployed people were re-employed, an increase of 4.8%; 74 thousand and 900 people with employment difficulties were employed, down by 3.0%.

The consumer price of the whole province rose by 1.6% over the previous year. Producer prices rose by 5.7%. The purchasing price of industrial producers rose by 11.2%.

**Table 2 Increase and Decrease of Consumer Price in 2022 over the Previous Year**

| **Indicator name** | **Increase or decrease over the previous year (%)** |
| --- | --- |
| **Consumer prices** | 1.6 |
| **# Food, tobacco and alcohol** | 1.0 |
| **Clothes** | 0.6 |
| **Live** | 0.5 |
| **Daily necessities and services** | 0.8 |
| **Traffic and communication** | 5.4 |
| **Education, culture and entertainment** | 1.5 |
| **Health care** | 0.3 |
| **Other supplies and services** | 1.4 |
|  |  |

At the end of the year, the total number of market entities in the province was 4 million 368 thousand and 700, an increase of 12.4% over the end of last year. The registered capital of market entities was 12.75 trillion yuan, an increase of 36.4%. In the whole year, 745400 new market entities were established, an increase of 6.3% over the previous year.

II. Agriculture

The total output value of agriculture, forestry, animal husbandry and fishery in the whole year was 490.867 billion yuan, an increase of 4.2% over the previous year. Among them, the total output value of planting industry was 331.370 billion yuan, an increase of 3.5%; the total output value of forestry was 34.00 billion yuan, an increase of 3.9%; the total output value of animal husbandry was 94.140 billion yuan, an increase of 5.7%; The total output value of fishery was 7.960 billion yuan, an increase of 6.5%.

**Table 3 Gross Output Value and Growth Rate of Agriculture, Forestry, Animal Husbandry and Fishery in 2022**

| **Indicator name** | **Absolute number (100 million yuan)** | **Year-on-year growth (%)** |
| --- | --- | --- |
| **Gross output value of agriculture, forestry, animal husbandry and fishery** | 4908.67 | 4.2 |
| **Planting** | 3313.70 | 3.5 |
| **Forestry** | 340.00 | 3.9 |
| **Animal husbandry** | 941.40 | 5.7 |
| **Fishery** | 79.60 | 6.5 |
| **Agriculture, Forestry, Animal Husbandry and Fishery Professional and Auxiliary Activities** | 233.97 | 6.3 |

The sown area of grain was 41,830,500 mu, an increase of 0.04% over the previous year; the grain output was 11,146,400 tons, an increase of 1.8% over the previous year. The planting area of vegetables in the whole year was 21.88 million mu, down 3.7% from the previous year, and the output of vegetables was 32.7597 million tons, up 2.3% from the previous year. The area of edible fungi harvested in the whole year was 363,300 mu, an increase of 4.2% over the previous year; the output of edible fungi was 797,600 tons, an increase of 1.6% over the previous year. The annual tea picking area was 5.4148 million mu, an increase of 1.7% over the previous year; the tea output was 266,200 tons, an increase of 8.3% over the previous year. The annual garden fruit picking area was 7 million 427 thousand and 200 mu, an increase of 1.5% over the previous year. The annual output of garden fruits was 6.2937 million tons, an increase of 7.9% over the previous year.

At the end of the year, there were 15.4267 million pigs, an increase of 0.8% over the end of the previous year; there were 4.9224 million cattle, an increase of 2.7%; there were 3.5915 million sheep, a decrease of 7.1%; the 12246 of poultry was 0.12 million, an increase of 1.9%. In the whole year, 19.8473 million pigs were slaughtered, an increase of 7.3% over the previous year; 1.7391 million cattle were slaughtered, a decrease of 3.4%; 2.6744 million sheep were slaughtered, a decrease of 4.5%; the 18422 of poultry was 0.68 million, an increase of 4.2%. The output of pork, beef, mutton and poultry was 2,390,500 tons, up by 5.9% over the previous year, and the output of eggs was 336,000 tons, up by 21.2%. Milk output was 37 thousand and 300 tons, down 24.1%.

The annual output of aquatic products in the whole province was 268 thousand and 400 tons, an increase of 2.4% over the previous year. Among them, the output of aquaculture products was 264 thousand and 100 tons, an increase of 2.7%.

**Table 4 Output and Growth Rate of Main Agricultural Products in 2022**

| **Indicator name** | **Absolute number (10,000 tons)** | **Year-on-year growth (%)** |
| --- | --- | --- |
| **Grain production** | 1114.64 | 1.8 |
| **# Summer grain** | 257.61 | 0.7 |
| **Vegetables and edible fungi** | 3355.73 | 2.3 |
| **Pork, beef, mutton and poultry meat** | 239.05 | 5.9 |

III. Industry and Construction

In the whole year, the total industrial added value of the province was 549.313 billion yuan, an increase of 0.7% over the previous year. The added value of industries above scale decreased by 0.5% over the previous year. Among the industries above the scale, the added value of state-owned holding enterprises increased by 17.8%, joint-stock enterprises decreased by 0.3%, foreign and Hong Kong, Macao and Taiwan-invested enterprises increased by 3.8%, and private enterprises decreased by 28.8%. In terms of categories, the mining industry decreased by 18.4%, the manufacturing industry increased by 4.1%, and the production and supply of electricity, heat, gas and water increased by 2.6%.

Of the 19 key industrial sectors monitored in the province, the added value of 6 industries has maintained growth. Among them, the added value of computer, communication and other electronic equipment manufacturing industry increased by 45.9% over the previous year, that of wine, beverage and refined tea manufacturing industry by 32.6%, that of electrical machinery and equipment manufacturing industry by 31.2%, that of railway, ship, aerospace and other transport equipment manufacturing industry by 12.8%, and that of tobacco products by 6.7%.

**Table 5 Growth rate of added value of major industries above designated size in 2022**

| **Indicator name** | **Year-on-year growth (%)** |
| --- | --- |
| **Industrial added value above designated size** | -0.5 |
| **# Coal mining and washing industry** | -17.6 |
| **Non-metallic mining and dressing industry** | -27.8 |
| **Agricultural and sideline food processing industry** | -25.3 |
| **Wine, beverage and refined tea manufacturing** | 32.6 |
| **Tobacco products industry** | 6.7 |
| **Chemical raw materials and chemical products manufacturing industry** | -14.4 |
| **Pharmaceutical manufacturing** | -12.6 |
| **Non-metallic mineral products industry** | -40.2 |
| **Ferrous metal smelting and calendering industry** | -26.0 |
| **Non-ferrous metal smelting and calendering industry** | -9.6 |
| **Automobile manufacturing** | -8.3 |
| **Electrical machinery and equipment manufacturing** | 31.2 |
| **Computer, communication and other electronic equipment manufacturing** | 45.9 |
| **Production and supply of electricity and heat** | 2.8 |
|  |  |

In the whole year, the province produced 2.7104 million smart TV sets, an increase of 16.1% over the previous year; ten kinds of non-ferrous metals were 1.5912 million tons, an increase of 10.7%; 212,600 tons of dairy products, up by 20.4%.

**Table 6 Output and Growth Rate of Major Industrial Products above Designated Size in 2022**

| **Indicator name (unit)** | **Absolute number** | **Year-on-year growth (%)** |
| --- | --- | --- |
| **Power generation (100 million kWh)** | 2184.06 | -2.6 |
| **# Wind power** | 105.33 | 3.0 |
| **Solar power generation** | 93.73 | 27.4 |
| **Biomass and garbage power generation** | 21.14 | 16.7 |
| **Raw coal (10,000 tons)** | 12813.63 | -4.0 |
| **Phosphate rock (containing 30% of phosphorus pentoxide) (10,000 tons)** | 1902.15 | -23.0 |
| **Beverage wine (thousands of liters)** | 97.28 | -8.9 |
| **Cigarettes (100 million)** | 1177.59 | 0.5 |
| **Chinese patent medicine (10,000 tons)** | 6.34 | -8.0 |
| **Dairy products (10,000 tons)** | 21.26 | 20.4 |
| **Chili products (10,000 tons)** | 32.54 | -37.7 |
| **Guitar (10,000)** | 107.23 | -31.4 |
| **Agricultural nitrogen, phosphorus and potassium chemical fertilizers (converted into pure) (10,000 tons)** | 247.37 | -18.7 |
| **Rubber tire casing (10,000 pieces)** | 659.42 | -7.9 |
| **Cement (10,000 tons)** | 6428.02 | -31.3 |
| **Pig iron (10,000 tons)** | 380.63 | -1.0 |
| **Steel (10,000 tons)** | 607.33 | -25.3 |
| **Ferroalloy (10,000 tons)** | 198.00 | -23.4 |
| **Ten kinds of non-ferrous metals (10,000 tons)** | 159.12 | 10.7 |
| **Primary aluminum (electrolytic aluminum) (10,000 tons)** | 140.61 | 5.7 |
| **Household refrigerators (10,000 units)** | 156.64 | -3.3 |
| **Integrated circuit (10,000 pieces)** | 38842.19 | -21.5 |
| **Electronic components (100 million)** | 68.14 | -10.4 |
| **Vehicles (10,000)** | 6.57 | -7.5 |
| **Smart TV (10,000 sets)** | 271.04 | 16.1 |

At the end of the year, the installed capacity of power generation in the whole province was 80.8727 million kilowatts, an increase of 6.8% over the end of the previous year. Among them, the installed capacity of thermal power was 37.9272 million kilowatts, an increase of 6.2%; the installed capacity of hydropower was 22.8232 million kilowatts, basically the same as the previous year; the installed capacity of grid-connected wind power was 5.9196 million kilowatts, an increase of 2.0%; The installed capacity of grid-connected solar power was 14.2027 million kilowatts, an increase of 24.9%.

In the whole year, the business income of industrial enterprises above the provincial scale reached 1064.950 billion yuan, an increase of 3.6% over the previous year. The total profits of industrial enterprises above designated size in the year totaled 132.032 billion yuan, up by 21.5% over the previous year. The annual profit margin of operating income was 12.4%, 1.8 percentage points higher than that of the previous year. The cost per 100 yuan of business income was 75.77 yuan, down 2.14 yuan. At the end of the year, the asset-liability ratio of industrial enterprises above designated size was 61.0%, down 1.3 percentage points from the end of last year.

At the end of the year, there were 2420 qualified general contracting and specialized contracting construction enterprises in the province, an increase of 249 over the end of the previous year. Among them, there were 144 construction enterprises with special-grade and first-grade qualifications, an increase of 14, and 1057 enterprises with second-grade qualifications, an increase of 110. There are 1219 three-level qualified enterprises and other qualified enterprises, an increase of 125.

IV. Investment in fixed assets

The investment in fixed assets of the whole province (excluding farmers) decreased by 5.1% over the previous year. Among them, investment in the primary industry decreased by 0.4%, investment in the secondary industry increased by 9.1%, and investment in the tertiary industry decreased by 10.0%. Industrial investment increased by 9.1%.

**Table 7 Growth rate of investment in fixed assets by industry (excluding farmers) in 2022**

| **Indicator name** | **Year-on-year growth (%)** |
| --- | --- |
| **Investment in fixed assets (excluding farmers)** | -5.1 |
| **# Agriculture, forestry, animal husbandry and fishery** | 4.7 |
| **Mining industry** | 5.9 |
| **# Coal mining and washing industry** | 6.0 |
| **Manufacturing** | 28.0 |
| **# Wine, Beverage and Refined Tea Manufacturing** | 35.3 |
| **Chemical raw materials and chemical products manufacturing industry** | 0.9 |
| **Pharmaceutical manufacturing** | 42.9 |
| **Ferrous metal smelting and calendering industry** | 26.9 |
| **Non-ferrous metal smelting and calendering industry** | -12.1 |
| **Production and supply of electricity, heat, gas and water** | -19.7 |
| **Transportation, storage and postal services** | 19.6 |
| **Management of water conservancy, environment and public facilities** | -3.8 |
| **Education** | 1.0 |
| **Health and Social Work** | 3.3 |
| **Information transmission, software and information technology services** | 26.7 |
| **Leasing and business services** | -35.5 |
| **Scientific research and technical services** | 95.8 |

V. Market Consumption

The total retail sales of social consumer goods in the whole province decreased by 4.5% over the previous year. According to the statistics of business places, the retail sales of consumer goods in cities and towns decreased by 4.9%, and the retail sales of consumer goods in rural areas decreased by 1.4%. According to the statistics of consumption types, the retail sales of commodities decreased by 4.7%, and the catering income decreased by 2.4%.

In the whole year, the retail sales of corporate enterprises (units) above designated size in the whole province decreased by 4.2% over the previous year. Among them, the retail sales of Chinese and Western medicines increased by 9.2%, petroleum and products by 4.7%, household appliances and audio-visual equipment by 0.8%, grain, oil and food by 1.2%, and cultural and office supplies by 6.4%. In the whole year, the retail sales of commodities realized by corporate enterprises (units) above designated size through public networks decreased by 6.7% over the previous year.

**Table 8 Growth rate of retail sales of corporate enterprises (units) above designated size in 2022**

| **Indicator name** | **Year-on-year growth (%)** |
| --- | --- |
| **Retail sales of corporate enterprises (units) above designated size** | -4.2 |
| **# Cereals, oils and foodstuffs** | -1.2 |
| **Alcohol and tobacco** | -10.1 |
| **Clothing, shoes and hats, knitted textiles** | -26.8 |
| **Cosmetics** | -1.0 |
| **Gold, silver and jewelry** | -12.7 |
| **Daily necessities** | -18.5 |
| **Sports and entertainment** | -6.0 |
| **Books, newspapers and magazines** | -1.7 |
| **Household appliances and audio and video equipment** | 0.8 |
| **Chinese and Western medicines** | 9.2 |
| **Cultural office supplies** | -6.4 |
| **Furniture** | -22.4 |
| **Communication equipment** | -15.2 |
| **Petroleum and its products** | 4.7 |
| **Construction and decoration materials** | -15.7 |
| **Cars** | -6.0 |
|  |  |

VI. Foreign Economy

The total import and export volume of the province in the whole year was 79.286 billion yuan, an increase of 21.2% over the previous year. The total export value was 51.981 billion yuan, up 6.7%, and the total import value was 27.306 billion yuan, up 63.6%. Of the total export volume, the general trade was 38.091 billion yuan, up 2.2%, and the processing trade was 9.359 billion yuan, up 37.4%. Of the total imports, general trade was 14 billion 876 million yuan, an increase of 74.4%; Processing trade was 7.372 billion yuan, an increase of 55.2%.

**Table 9 Total Import and Export Volume and Its Growth Rate in 2022**

| **Indicator name** | **Absolute number (100 million yuan)** | **Year-on-year growth (%)** |
| --- | --- | --- |
| **Total imports and exports** | **792.86** | **21.2** |
| **Total imports** | 273.06 | 63.6 |
| **# General trade** | 148.64 | 74.4 |
| **Processing trade** | 73.72 | 55.2 |
| **Total exports** | 519.81 | 6.7 |
| **# General trade** | 380.91 | 2.2 |
| **Processing trade** | 93.59 | 37.4 |
|  |  |  |

In the whole year, 105 foreign-invested enterprises were newly established in the province, and the actual amount of foreign capital used was 531 million US dollars. The total investment of foreign-invested enterprises at the end of the year was 215.568 billion US dollars. The registered capital at the end of the period is 166.964 billion US dollars, of which 153.885 billion US dollars are subscribed by foreign parties.

VII. Transportation

At the end of the year, the provincial highway traffic mileage was 209600 kilometers, an increase of 1.2% over the end of the previous year. Among them, 8331 kilometers of expressways were open to traffic, an increase of 4.0%. At the end of the year, the length of inland waterways was 3,954 km.

The total volume of railway, highway and waterway cargo transportation in the whole year was 94998 5900 tons, down 2.1% from the previous year. The cargo turnover was 141.727 billion ton-kilometers, down by 1.3% over the previous year. Civil aviation cargo and postal throughput was 84 thousand and 800 tons, down 29.3% from the previous year.

**Table 10 Cargo Transport Volume and Growth Rate of Various Transport Modes in 2022**

| **Indicator name (unit)** | **Absolute number** | **Year-on-year growth (%)** |
| --- | --- | --- |
| **Total volume of goods transported (10,000 tons)** | 94998.59 | -2.1 |
| **Railway** | 6671.98 | -8.3 |
| **Highway** | 87870.39 | -1.4 |
| **Water transport** | 456.21 | -18.5 |
| **Cargo turnover (100 million ton-kilometers)** | 1417.27 | -1.3 |
| **Railway** | 680.18 | -0.8 |
| **Highway** | 722.91 | -0.5 |
| **Water transport** | 14.18 | -40.3 |
| **Cargo and mail throughput of civil aviation (10,000 tons)** | 8.48 | -29.3 |
|  |  |  |

The total volume of railway, highway and waterway passenger transport in the whole year was 20322 5900, down 21.4% from the previous year. The passenger turnover was 31.987 billion person-kilometers, down by 22.0%. Civil aviation handled 12.3157 million passengers, down 44.6% from the previous year.

**Table 11 Passenger traffic volume completed by various modes of transport in 2022 and its growth rate**

| **Indicator name (unit)** | **Absolute number** | **Year-on-year growth (%)** |
| --- | --- | --- |
| **Total volume of passenger transport (10,000 persons)** | 20322.59 | -21.4 |
| **Railway** | 4580.23 | -29.3 |
| **# High speed rail** | 3763.78 | -26.4 |
| **Highway** | 15537.09 | -18.2 |
| **Water transport** | 205.27 | -44.5 |
| **Passenger turnover (100 million person-km)** | 319.87 | -22.0 |
| **Railway** | 207.20 | -18.9 |
| **# High speed rail** | 166.14 | -15.3 |
| **Highway** | 112.24 | -27.1 |
| **Water transport** | 0.43 | -52.0 |
| **Civil aviation passenger throughput (10,000 person-times)** | 1231.57 | -44.6 |
|  |  |  |

VIII. Postal Communication

In the whole year, the total business volume of the postal industry in the province was 9.627 billion yuan, an increase of 9.6% over the previous year. In the whole year, 24.0881 million pieces of mail business were completed, down 2.9% from the previous year; 492 million pieces of express delivery were received and sent, up 23.7%; Express business revenue was 7.267 billion yuan, an increase of 9.0%.

The total volume of telecommunications business in the whole province was 44.76 billion yuan, an increase of 25.4% over the previous year. At the end of the year, there were 44.479 million mobile phone users and 38,000 Gbps of Internet bandwidth, up by 35.7%. The length of optical cable lines was 1.67 million km, an increase of 24.0%; The number of 5G base stations has reached 84693.

Ix. Finance and banking

The total fiscal revenue of the province in the whole year was 319.269 billion yuan, a decrease of 6.5% over the previous year, and an increase of 6.2% in the same caliber after deducting the VAT rebate factor (the same below). The general public budget revenue was 188.641 billion yuan, a decrease of 4.2% over the previous year and an increase of 6.8% over the same caliber. Among them, tax revenue was 102 billion 176 million yuan, down 13.2%, an increase of 5.3% in the same caliber.

In the whole year, the general public budget expenditure of the province was 585 billion 136 million yuan, an increase of 4.7% over the previous year. Of this, transportation expenditure was 37.248 billion yuan, an increase of 10.7%; health expenditure was 58.359 billion yuan, an increase of 7.7%; social security and employment expenditure was 73.846 billion yuan, an increase of 7.2%; culture, tourism, sports and media expenditure was 12.358 billion yuan, an increase of 5.5%; Expenditure on education was 115 billion 533 million yuan, an increase of 2.3%.

At the end of the year, the balance of RMB deposits in financial institutions in the whole province was 3276.105 billion yuan, an increase of 9.0% over the same period last year. Among them, household deposits 16320 50 million yuan, an increase of 14.8%. At the end of the year, the balance of RMB loans of financial institutions was 40223 109 million yuan, an increase of 12.3% over the same period last year. Among them, household loans amounted to 1212.711 billion yuan, an increase of 8.5%.

**Table 12 Balance and Growth Rate of RMB Deposits and Loans of Financial Institutions at the End of 2022**

| **Indicator name** | **Absolute number (100 million yuan)** | **Year-on-year growth (%)** |
| --- | --- | --- |
| **Balance of various deposits** | **32761.05** | **9.0** |
| **# Domestic deposits** | 32751.08 | 9.0 |
| **Household deposits** | 16320.50 | 14.8 |
| **Deposits of non-financial enterprises** | 8604.83 | 3.9 |
| **Balance of various loans** | **40223.09** | **12.3** |
| **# Domestic loans** | 40222.46 | 12.3 |
| **Household loans** | 12127.11 | 8.5 |
| **Short-term loan** | 3303.65 | 10.7 |
| **Medium and long-term loans** | 8823.45 | 7.7 |
| **Loans to enterprises (institutions)** | 28095.34 | 14.0 |
| **Short-term loan** | 3374.57 | 18.3 |
| **Medium and long-term loans** | 23625.71 | 12.2 |

In the whole year, the original premium income of insurance companies in the whole province was 50.424 billion yuan, an increase of 1.6% over the previous year. Of this total, the premium of property insurance was 22.993 billion yuan, and that of life insurance was 27.431 billion yuan. The annual compensation expenditure was 21.808 billion yuan, an increase of 5.4% over the previous year. Among them, 14.374 billion yuan was paid for property insurance and 7.434 billion yuan for life insurance.

X. People's livelihood and social security

In the whole year, the per capita disposable income of residents in the whole province was 25508 yuan, an increase of 6.3% over the previous year. By permanent residence, the per capita disposable income of urban residents was 41086 yuan, an increase of 4.8%; The per capita disposable income of rural residents was 13707 yuan, an increase of 6.6%.

**Table 13 Composition and Growth Rate of Per Capita Disposable Income of Urban and Rural Residents in 2022**

| **Indicator name** | **Absolute number (yuan)** | **Year-on-year growth (%)** |
| --- | --- | --- |
| **Per capita disposable income of urban residents** | 41086 | 4.8 |
| **Wage income** | 23448 | 4.3 |
| **Net income from operations** | 7232 | 7.1 |
| **Net income from property** | 3309 | -2.8 |
| **Net income transferred** | 7097 | 8.2 |
| **Per capita disposable income of rural residents** | 13707 | 6.6 |
| **Wage income** | 5585 | 4.8 |
| **Net income from operations** | 4219 | 7.8 |
| **Net income from property** | 117 | -6.4 |
| **Net income transferred** | 3787 | 8.5 |

The annual per capita consumption expenditure of residents in the whole province was 17939 yuan, down 0.1% from the previous year. By permanent residence, the per capita consumption expenditure of urban residents was 24230 yuan, down 4.4%; The per capita consumption expenditure of rural residents was 13172 yuan, an increase of 4.9%.

At the end of the year, every 100 urban households owned 54.85 household automobiles, up 3.7% from the end of the previous year, and 50.86 air conditioners, up 5.9%. At the end of the year, every 100 rural households owned 26.24 household automobiles, up 11.4%, and 57.9 motorcycles, down 1.4%. There were 81.3 water heaters, up 4.8%.

At the end of the year, the per capita housing area of urban residents in the province was 42.64 square meters, an increase of 0.62 square meters over the end of the previous year. The per capita housing area of rural residents is 45.44 square meters, a decrease of 4.39 square meters.

**Table 14 Number of Durable Consumer Goods Owned per 100 Households and Its Growth Rate at the End of 2022**

| **Indicator name (unit)** | **Absolute number** | **Increase over the end of last year (%)** |
| --- | --- | --- |
| **Number of durable consumer goods owned per 100 urban households** |  |  |
| **Water heater (set)** | 101.73 | 1.2 |
| **Air conditioner (set)** | 50.86 | 5.9 |
| **Computer (set)** | 53.38 | 2.8 |
| **Mobile phone (unit)** | 299.28 | 0.2 |
| **Color TV set** | 106.27 | 1.0 |
| **Refrigerator (cabinet) (set)** | 105.48 | 1.3 |
| **Motorcycle (unit)** | 22.61 | 1.2 |
| **Family car (unit)** | 54.85 | 3.7 |
| **Number of durable consumer goods owned per 100 rural households** |  |  |
| **Washing machine (set)** | 100 | 1.5 |
| **Motorcycle (unit)** | 57.9 | -1.4 |
| **Family car (unit)** | 26.24 | 11.4 |
| **Color TV set** | 100 | 1.6 |
| **Water heater (set)** | 81.3 | 4.8 |
| **Fixed telephone (unit)** | 0.21 | -50.3 |
| **Mobile phone (unit)** | 319.8 | 2.2 |
| **Refrigerator (cabinet) (set)** | 101.8 | 2.9 |

At the end of the year, 19.3553 million urban and rural residents participated in basic old-age insurance, an increase of 0.4% over the end of the previous year. The number of urban workers participating in basic old-age insurance was 7.707 million, an increase of 1.9%. 3.387 million people participated in unemployment insurance, an increase of 5.5%. The number of people participating in basic medical insurance was 42.2123 million. The number of people participating in industrial injury insurance was 5.9384 million, an increase of 12.1%.

At the end of the year, there were 578,900 urban residents receiving the minimum living allowance in the province; the monthly per capita allowance was 679 yuan, an increase of 3.5% over the previous year. At the end of the year, 1.7019 million rural residents received subsistence allowances; The annual per capita security standard was 5292 yuan, an increase of 15.8% over the previous year.

At the end of the year, there were 1030 social service institutions providing accommodation in the province, including 937 old-age institutions and 34 child welfare and relief and protection institutions. At the end of the year, there were 92,400 beds in civil affairs institutions providing accommodation, including 83,200 beds in old-age care institutions and 4,254 beds in child welfare and relief and protection institutions.

Xi. Tourism and culture

In the whole year, the province received 492 million tourists, with a total tourism income of 524.564 billion yuan.

At the end of the year, there were 9 5A-level tourist attractions and 143 4A-level tourist attractions in the province. At the end of the year, there were 81 key cultural relics protection units, 437 key villages (towns) of rural tourism above grade, and 8957 standardized units of rural tourism above grade. At the end of the year, the number of guest rooms was 884,100, and the number of beds in guest rooms was 1,436,200.

At the end of the year, there were 101 art performance groups, 23 art performance venues, 133 museums, 99 public libraries, 1701 mass art galleries and cultural centers (stations) in the province. At the end of the year, the comprehensive population coverage rate of television was 99.24%, and that of radio was 99.02%. 136 million copies of books and 14.25 million copies of periodicals were published in the whole year.

XII. Education and Science and Technology

At the end of the year, there were 6,470 primary schools with 3,915,400 students, 1,902 junior middle schools with 1,898,500 students, and 494 senior middle schools with 950,600 students. There are 183 secondary vocational schools with 524,400 students, 75 institutions of higher learning with 894,700 students, and 10 postgraduate training units with 36,800 postgraduates.

**Table 15 Enrollment, Enrolment and Growth Rate of Enrolled Students of All Levels and Types of Schools in 2022**

| **Indicator name** | **Absolute number (10,000 persons)** | **Year-on-year growth (%)** |
| --- | --- | --- |
| **Enrollment of all kinds of schools at all levels** |  |  |
| **# Graduate students** | 1.34 | 9.8 |
| **Regular institutions of higher learning** | 27.93 | -4.0 |
| **Adult Higher Education** | 2.51 | -13.4 |
| **Secondary Vocational Education** | 28.38 | 79.8 |
| **Ordinary high school** | 30.85 | -6.8 |
| **Junior middle school** | 66.58 | 3.6 |
| **Ordinary primary school** | 60.92 | -2.4 |
| **Number of students in schools of all levels and types** |  |  |
| **# Graduate students** | 3.68 | 16.1 |
| **Regular institutions of higher learning** | 89.47 | 1.5 |
| **Adult Higher Education** | 7.31 | -16.0 |
| **Secondary Vocational Education** | 52.44 | 31.9 |
| **Ordinary high school** | 95.06 | -1.6 |
| **Junior middle school** | 189.85 | 5.5 |
| **Ordinary primary school** | 391.54 | -1.2 |

At the end of the year, the province had five national science and technology cooperation bases, 93 academician workstations, six national key laboratories and one national key laboratory. In the whole year, 855.4 technology contracts were registered, up by 53.0% over the previous year, and the transaction value of contracts was 39.073 billion yuan, up by 35.1%. 193 scientific and technological achievements at or above the provincial and ministerial levels were registered, down by 3.0%; 293 patents were granted, down 25.2%.

XIII. Health and Physical Education

At the end of the year, there were 29150 medical and health institutions in the province, including 2826 hospitals and health centers; There are 344 professional public health institutions, including 101 centers for disease control and prevention. At the end of the year, there were 309703 beds in medical and health institutions, including 290,489 beds in hospitals and health centers. At the end of the year, there were 321,400 health technicians, including 109,400 practicing (assistant) doctors and 147,100 registered nurses.

**Table 16 Medical and health institutions, beds, health technicians and their growth rate at the end of 2022**

| **Indicator name (unit)** | **Absolute number** | **Increase over the end of last year (%)** |
| --- | --- | --- |
| **Health institutions (unit)** | 29150 | -0.5 |
| **# Hospitals and health centers** | 2826 | Flat |
| **Health technical personnel (10,000 persons)** | 32.14 | 3.9 |
| **# Medical Practitioner (Assistant)** | 10.94 | 3.8 |
| **Registered nurse** | 14.71 | 3.9 |
| **Beds in health institutions (PCs.)** | 309703 | 4.3 |
| **# Hospitals and health centers** | 290489 | 4.1 |
|  |  |  |

Guizhou athletes won 105 awards in major international and domestic sports competitions throughout the year. Among them, two world champions and 33 gold medals in the highest level competitions in China were won. There are 108,300 sports venues in the province.

XIV. Ecological Construction and Environment

At the end of the year, there were 88 nature reserves in the province. Among them, there are 11 national nature reserves. At the end of the year, the area of nature reserves was 847,000 hectares. The afforestation area of the whole year was 183,300 hectares, and the forest coverage rate of the whole province was 62.81% at the end of the year.

The proportion of days with good air quality in the central cities of the province was 99.1%. The excellent rate of water quality in the exit sections of major rivers is 100%. The sewage treatment rate of cities (counties) and the harmless treatment rate of domestic waste increased to 98.2% and 99.3% respectively. The energy consumption of 10000 yuan GDP decreased by 3.7% compared with the previous year. A total of 321400 hectares of soil erosion control area have been added.

Note:

1. The data in this bulletin are preliminary statistics.

2. The growth rate of GDP, industrial added value above scale and its classified items is calculated at comparable prices, which is the actual growth rate; Unless otherwise specified, other indicators are nominal growth rates calculated at current prices.

3. Gross regional product refers to the total value of all final products and services produced by all resident units in a region in a certain period of time.

4. The statistical scope of industries above designated size is industrial enterprises with annual main business income of 20 million yuan or more.

5. Investment in fixed assets (excluding farmers) refers to the total amount of work completed in a certain period of time in the form of money to build and purchase fixed assets and the related costs. The statistical scope of investment in fixed assets (excluding farmers) is investment in fixed assets projects with a total planned investment of more than 5 million yuan and investment in all real estate development projects.

6. Units above the quota in the retail statistics of social consumer goods refer to wholesale enterprises (units) with annual main business income of 20 million yuan or more, retail enterprises (units) with annual main business income of 5 million yuan or more, accommodation and catering enterprises (units) with annual main business income of 2 million yuan or more.

7. Consumer price index refers to the relative number reflecting the trend and degree of changes in the prices of consumer goods and services purchased by urban and rural residents in a certain period of time.

8. Due to the rounding of some data, there is a difference between the total and the sub-total.

Source:

The employment, social security and other data in this bulletin are from the Provincial Human Resources and Social Security Department; the market subject and patent data are from the Provincial Market Supervision and Administration Bureau; the afforestation area, forest coverage and nature reserve data are from the Provincial Forestry Bureau; the aquatic product output data are from the Provincial Department of Agriculture and Rural Areas; the import and export data are from Guiyang Customs; The data of actually used foreign capital and foreign economic and technological cooperation are from the Provincial Department of Commerce; the data of highway transportation and water transportation are from the Provincial Department of Transportation; the data of railway transportation are from the National Bureau of Statistics, which are full-caliber data; the data of civil aviation are from the Provincial Airport Group Co., Ltd.; the data of postal services are from the Provincial Postal Administration; the data of telecommunications are from the Provincial Communications Administration; the data of finance are from the Provincial Department of Finance; Financial data are from Guiyang Central Branch of the People's Bank of China; securities and futures data are from Guizhou Regulatory Bureau of China Securities Regulatory Commission; insurance data are from Guizhou Regulatory Bureau of China Banking and Insurance Regulatory Commission; medical insurance data are from Provincial Medical Security Bureau; urban and rural minimum living allowance and social service data are from Provincial Department of Civil Affairs; The data of tourism, art performance groups and venues are from the Provincial Department of Culture and Tourism; the data of television and radio are from the Provincial Radio and Television Bureau; the data of books and periodicals are from the Publicity Department of the Provincial Committee; the data of education are from the Provincial Department of Education; the data of science and technology are from the Provincial Department of Science and Technology; the data of medical and health are from the Provincial Health Commission; the data of sports are from the Provincial Sports Bureau; the data of environment are from the Provincial Department of Ecological Environment; The data of comprehensive control of soil erosion are from the Provincial Department of Water Resources. The data of urban (county) sewage treatment rate and harmless treatment rate of domestic waste are from the Provincial Housing and Construction Department. The data of installed capacity of power generation comes from the Provincial Electric Power Industry Association.

[[Back to top]](javascript:scroll(0,0)) [[Print this page]](javascript:window.print();) [[Close this page]](javascript:window.close())

Previous:[Statistical Communique on R & D Investment in Guizhou Province in 2022](http://hgk.guizhou.gov.cn/publish/articles/c7/2023/10/a1022/a1022.html?locationhref=http://hgk.guizhou.gov.cn/publish/channels/c7/c7_1psSuffix&pagesize=15&curpage=1&curainum=1)
Next:[Statistical Communique on R & D Investment in Guizhou Province in 2021](http://hgk.guizhou.gov.cn/publish/articles/c7/2022/10/a858/a858.html?locationhref=http://hgk.guizhou.gov.cn/publish/channels/c7/c7_1psSuffix&pagesize=15&curpage=1&curainum=3)

Sponsor: Guizhou Provincial Bureau of Statistics Technical Support: Guizhou Jiawang Technology Development Co., Ltd.

Record No.: Qian ICP 19000889 No.: -3

Current location:[Home page](http://hgk.guizhou.gov.cn/index.vhtml) > [Statistical publications](http://hgk.guizhou.gov.cn/publish/channels/c6/c6_1.html)> [Statistical Bulletin](http://hgk.guizhou.gov.cn/publish/channels/c7/c7_1.html)> [Statistical Bulletin on the National Economic and Social Development of Guizhou Province in 2017](http://hgk.guizhou.gov.cn/publish/articles/c7/2023/09/a406/a406.html)

Statistical Bulletin

Statistical Bulletin on the National Economic and Social Development of Guizhou Province in 2017

Date: 2018-04-04 Article Source: Provincial Bureau of Statistics No.: [[Big](javascript:doZoom(20)) [Medium](javascript:doZoom(16)) [Small](javascript:doZoom(12)) ]

Guizhou Provincial Bureau of Statistics National Bureau of Statistics Guizhou Investigation Team
(4 April 2018)
　　 
In 2017, under the strong leadership of the provincial Party Committee and the provincial government, the whole province adhered to the guiding ideology of socialism with Chinese characteristics in the new era of , conscientiously implemented the spirit of the Nineteenth National Congress of the Party and the spirit of General Secretary 's important speech in the delegation of Guizhou Province, comprehensively implemented the decision-making and deployment of the central and provincial Party committees and provincial governments, and adhered to the general tone of steady and progressive work. Adhere to the overall situation of economic and social development, adhere to the supply-side structural reform as the main line, adhere to the two bottom lines of development and ecology, vigorously promote the three strategic actions of poverty alleviation, big data and big ecology, actively adapt to the new normal, actively respond to new challenges, effectively resolve new contradictions, and the province's economic operation. New achievements have been made in economic and social development.
First, comprehensive
Preliminary accounting shows that in 2017, the GDP of the whole province was 13540 83 million yuan, an increase of 10.2% over the previous year. By industry, the added value of the primary industry was 202.078 billion yuan, up 6.7%; the added value of the secondary industry was 543.963 billion yuan, up 10.1%; the added value of the tertiary industry was 608.042 billion yuan, up 11.5%. The added value of the primary industry accounted for 14.9% of the GDP, and the added value of the secondary industry accounted for 40.2%. The added value of the tertiary industry accounted for 44.9%. Per capita GDP was 37956 yuan, an increase of 4710 yuan over the previous year.
Figure 1 GDP and its growth rate from 2013 to 2017
　　
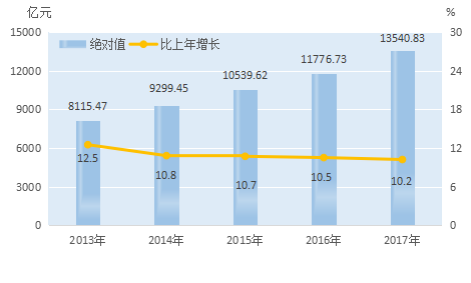

Figure 2 The proportion of the added value of the three industries in the GDP of the region from 2013 to 2017
　　
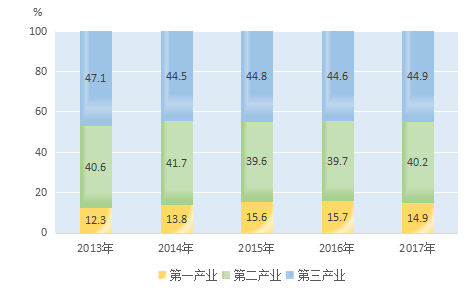

At the end of the year, the permanent population was 35.8 million, an increase of 250000 over the end of the previous year. Among them, the population aged 0-14 accounted for 22.4% of the permanent population at the end of the year, 67.2% of the population aged 15-64, and 10.4% of the population aged 65 and over. Regionally, the urban permanent population was 16.4752 million, accounting for 46.02% of the permanent population at the end of the year (the urbanization rate of the permanent population), an increase of 1.87 percentage points over the end of the previous year. The ratio of male to female (100 females) was 106.45. The annual number of births was 500,500, with a birth rate of 13.98 ‰; the number of deaths was 246,300, with a death rate of 6.88 ‰; and the natural growth rate was 7.10 ‰.
Table 1 Number and composition of resident population at the end of 2017

| Indicator name | Absolute number (10,000 persons) | As a percentage of the resident population at the end of the year Specific gravity (%) |
| --- | --- | --- |
| Resident population at the end of the year | 3580 | 100 |
| By urban and rural areas |  |  |
| Towns | 1647.52 | 46.02 |
| Rural | 1932.48 | 53.98 |
| By gender |  |  |
| Male | 1845.92 | 51.56 |
| Female | 1734.08 | 48.44 |
| By age |  |  |
| 0-14 years old (including less than 15 years old) | 802.28 | 22.41 |
| 15-64 years old (including less than 65 years old) | 2405.40 | 67.19 |
| 65 years and above | 372.32 | 10.40 |

769000 new jobs were created in cities and towns throughout the year, an increase of 1.5% over the previous year. Among them, 143 thousand and 900 unemployed people were re-employed, and 78 thousand and 200 people with employment difficulties were employed. At the end of the year, the registered urban unemployment rate was 3.23%.
At the end of the year, there were 2.4956 million market entities, an increase of 13.4% over the end of the previous year. Among them, 694900 new registered market entities were registered, an increase of 89.1% over the end of last year.
Table 2 Total number of newly registered market entities and their growth rate in 2017

| Indicator name | Absolute number (10,000 households) | Year-on-year growth (%) |
| --- | --- | --- |
| Total amount of newly registered market entities | 69.49 | 89.1 |
| # Domestic-funded enterprises | 13.07 | 33.2 |
| # Private enterprises | 12.02 | 34.0 |
| Foreign-funded enterprises | 0.03 | 29.8 |
| Individual industrial and commercial households and farmers' professional cooperatives | 56.39 | 111.3 |

II. Overcoming Poverty
The province took poverty alleviation as the overall situation of economic and social development, vigorously launched the "spring offensive", "summer contest" and "autumn offensive". At the end of the year, 2.8032 million rural poor people in the province were lifted out of poverty, 1.2369 million rural poor people were lifted out of poverty throughout the year, and the incidence of poverty dropped to 7.75%, down 3.17 percentage points from the end of last year.
Figure 3 Rural Poverty Population and Incidence of Poverty, 2013-2017
　　
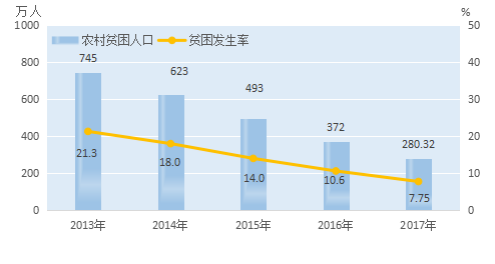

Start the three-year decisive battle of the rural "group-to-group" highway and build the "group-to-group" highway
25,000 kilometers. 763000 people were relocated and 15000 industrial poverty alleviation projects were implemented. 2.5745 million people enjoyed the "four-fold medical security", 200000 rural dilapidated houses were renovated, and 832500 students from poor families were subsidized.
3. Agriculture
In 2001, the added value of agriculture, forestry, animal husbandry and fishery was 212.848 billion yuan, up by 6.5% over the previous year.
Table 3 The added value and growth rate of agriculture, forestry, animal husbandry and fishery in 2017

| Indicator name | Absolute number (100 million yuan) | Year-on-year growth (%) |
| --- | --- | --- |
| Added value of agriculture, forestry, animal husbandry and fishery | 2128.48 | 6.5 |
| Planting | 1287.17 | 7.4 |
| Forestry | 155.45 | 7.9 |
| Animal husbandry | 531.08 | 4.4 |
| Fishery | 47.07 | 7.4 |
| Agriculture, forestry, animal husbandry and fishery services | 107.71 | 3.9 |

In 2001, the added value of planting industry was 128.717 billion yuan, up by 7.4% over the previous year. The grain planting area was 45.7685 million mu, a decrease of 930500 mu over the previous year. Among them, the rice planting area is 9.9192 million mu, a decrease of 194,700 mu; the corn planting area is 10.7291 million mu, a decrease of 375,800 mu. Among the cash crops, the planting area of vegetables and edible fungi is 17.2196 million mu, an increase of 1.463 million mu; the planting area of garden fruits is 5.9379 million mu, an increase of 1.0755 million mu; the planting area of tea is 7.153 million mu, an increase of 555,700 mu; The planting area of Chinese herbal medicines was 2.8593 million mu, an increase of 334,800 mu.
Table 4 Planting area and growth rate of grain crops in 2017

| Indicator name | Planting area (ten thousand mu) | Year-on-year growth (%) |
| --- | --- | --- |
| Food crops |  |  |
| # Rice | 991.92 | -1.9 |
| Corn | 1072.91 | -3.4 |
| Wheat | 334.23 | -7.8 |

Table 5 Planting area of cash crops and its growth rate in 2017

| Indicator name | Planting area (ten thousand mu) | Year-on-year growth (%) |
| --- | --- | --- |
| Cash crop |  |  |
| # Vegetables and Edible Fungi | 1721.96 | 9.3 |
| Fruit | 593.79 | 22.1 |
| Tea | 715.30 | 8.4 |
| Chinese herbal medicine | 285.93 | 13.3 |

The total grain output of the year was 11.7854 million tons. Among them, the output of rice was 4.237 million tons and the output of corn was 3.1356 million tons. Among the main cash crops, the output of tea was 176,500 tons, an increase of 24.9% over the previous year; The output of Chinese herbal medicines was 522,100 tons, an increase of 21.3%.
In 2001, the added value of forestry was 15.545 billion yuan, up by 7.9% over the previous year. The afforestation area is 10.002 million mu, and the output of commercial timber is 2.4855 million cubic meters.
In 2001, the added value of animal husbandry was 53.108 billion yuan, up by 4.4% over the previous year. The output of pork, beef, mutton and poultry was 2.0275 million tons, up by 4.0% over the previous year; the output of poultry eggs was 186,900 tons, up by 2.1%; the output of milk was 65,600 tons, up by 2.7%. At the end of the year, the number of live pigs was 15.9689 million, an increase of 6.6% over the end of the previous year; the number of cattle was 4.9235 million, a decrease of 5.0% over the end of the previous year. 18.2515 million pigs were slaughtered in the whole year, an increase of 3.7% over the previous year; 1,509,900 cattle were sold, an increase of 7.3%.
The added value of fishery in the whole year was 4.707 billion yuan, an increase of 7.4% over the previous year. The output of aquatic products was 299,600 tons, an increase of 3.4% over the previous year. Among them, the output of aquaculture products was 286 thousand and 400 tons, an increase of 3.8%.
The total power of agricultural machinery in the whole year was 28.07 million kilowatts, and the water-saving irrigation area increased by 100,100 mu.
IV. Industry and Construction
At the end of the year, there were 5637 industrial corporate enterprises above designated size, an increase of 590 over the end of the previous year. Among them, there are 7 and 2 enterprises whose annual main business income exceeds 10 billion yuan and 50 billion yuan respectively.
In the whole year, the added value of industries above designated size was 430.480 billion yuan, an increase of 9.5% over the previous year. Among them, the added value of light industry was 170.859 billion yuan, an increase of 12.2% over the previous year, accounting for 39.7% of the added value of industries above scale. The added value of heavy industry was 259.622 billion yuan, an increase of 7.7%, accounting for 60.3% of the added value of industries above scale.
Figure 4 Industrial added value above designated size and its growth rate from 2013 to 2017
　　
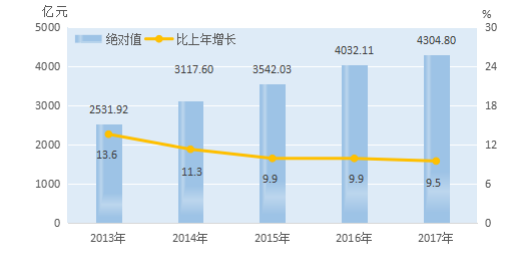

Among the industries above the scale, the added value of the wine, beverage and refined tea manufacturing industry increased by 13.5% over the previous year, that of the power, thermal production and supply industry increased by 13.0%, that of the tobacco products industry increased by 2.0%, that of the coal mining and washing industry decreased by 4.9%, and that of the four traditional industries of coal, electricity, tobacco and alcohol totaled 242.291 billion yuan. It accounts for 56.3% of the industrial added value above the scale. Among them, the added value of wine, beverage and refined tea manufacturing industry is 89.715 billion yuan, accounting for 20.8% of the industrial added value above the scale, which is the largest pillar industry.
Table 6 The added value and growth rate of major industries above designated size in 2017

| Indicator name | Absolute number (100 million yuan) | Year-on-year growth (%) |
| --- | --- | --- |
| Industrial added value above designated size | 4304.80 | 9.5 |
| # Coal mining and washing industry | 777.40 | -4.9 |
| Non-metallic mining and dressing industry | 108.88 | 9.0 |
| Agricultural and sideline food processing industry | 77.78 | 11.8 |
| Wine, beverage and refined tea manufacturing | 897.15 | 13.5 |
| Tobacco products industry | 288.04 | 2.0 |
| Chemical raw materials and chemical products manufacturing industry | 166.58 | 4.2 |
| Pharmaceutical manufacturing | 148.30 | 21.3 |
| Non-metallic mineral products industry | 358.69 | 6.5 |
| Ferrous metal smelting and calendering industry | 84.42 | -4.6 |
| Non-ferrous metal smelting and calendering industry | 131.48 | 10.5 |
| Computer, communication and other electronic equipment manufacturing | 118.64 | 86.3 |
| Production and supply of electricity and heat | 460.31 | 13.0 |
| Automobile manufacturing | 75.60 | 19.1 |

The added value of high-tech industries in the whole year increased by 39.9% over the previous year, accounting for 8.1% of the added value of industries above scale, an increase of 1.3 percentage points over the previous year. The added value of computer, communication and other electronic equipment manufacturing industry, pharmaceutical manufacturing industry and automobile manufacturing industry was 11.864 billion yuan, 14.830 billion yuan and 7.560 billion yuan respectively, up 86.3%, 21.3% and 19.1% respectively over the previous year, accounting for 2.8%, 3.4% and 1.8% of the added value of industries above scale.
Table 7 Output and Growth Rate of Major Industrial Products above Designated Size in 2017

| Indicator name (unit) | Absolute number | Year-on-year growth (%) |
| --- | --- | --- |
| Power generation (100 million kWh) | 1856.53 | 3.5 |
| Phosphate rock (containing 30% of phosphorus pentoxide) (10,000 tons) | 4817.00 | 10.9 |
| Beverage wine (thousands of liters) | 143.90 | 6.5 |
| # White wine | 45.21 | 10.0 |
| Cigarettes (100 million) | 1076.05 | -7.3 |
| Chinese patent medicine (10,000 tons) | 9.78 | 15.8 |
| Multicolor printed matter (ten thousand folio color order) | 466.93 | 4.8 |
| Coke (10,000 tons) | 510.21 | -19.1 |
| Agricultural nitrogen, phosphorus and potassium chemical fertilizers (converted into pure) (10,000 tons) | 538.47 | -1.1 |
| Rubber tire casing (10,000 pieces) | 497.59 | 0.7 |
| Cement (10,000 tons) | 11356.51 | 8.2 |
| Pig iron (10,000 tons) | 343.71 | -4.6 |
| Steel (10,000 tons) | 495.72 | 1.1 |
| Ferroalloy (10,000 tons) | 251.35 | -4.0 |
| Ten kinds of non-ferrous metals (10,000 tons) | 109.35 | 53.5 |
| Primary aluminum (electrolytic aluminum) (10,000 tons) | 101.85 | 63.5 |
| Household refrigerators (10,000 units) | 125.90 | 0.9 |
| Integrated circuit (10,000 pieces) | 15175.89 | 29.7 |
| Vehicle (unit) | 89878 | -0.7 |
| Color TV sets (10,000 sets) | 205.45 | 7.4 |
| Smartphones (10,000 units) | 1855.66 | 693.0 |

At the end of the year, the installed capacity of electric power was 58.4252 million kilowatts, an increase of 3.024 million kilowatts over the end of the previous year. Among them, the installed capacity of hydropower was 21 million 192 thousand and 300 kilowatts, an increase of 403 thousand kilowatts; The installed capacity of wind power was 3.6338 million kilowatts, an increase of 18,000 kilowatts.
The main business income of the industries above the designated scale in the whole year was 0.95 billion yuan 11300, an increase of 18.7% over the previous year, and the profits were 88.632 billion yuan, an increase of 46.4%. Profits and taxes reached 169.326 billion yuan, an increase of 31.6%. The rate of industrial production and sales was 97.2%.
At the end of the year, there were 1165 qualified general contracting and specialized contracting construction enterprises, an increase of 154 over the end of the previous year. Among them, there are 65 special-grade and first-grade construction enterprises, an increase of 2; 403 second-grade qualified enterprises, an increase of 69; There were 697 enterprises with three-level qualifications, an increase of 83. The total output value of construction industry was 293 billion 296 million yuan, an increase of 24.1%.
Figure 5 Total output value of construction industry and its growth rate from 2013 to 2017
　　
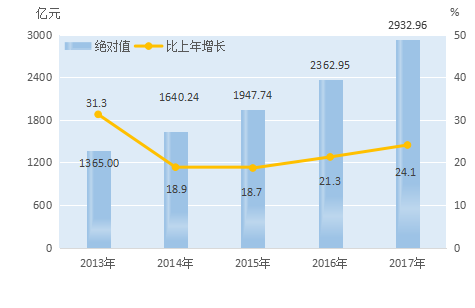

V. Investment in fixed assets
The annual investment in fixed assets was 1.55 trillion yuan, an increase of 20.1% over the previous year. The investment in the primary industry increased by 29.9% over the previous year, accounting for 2.5% of the province's total investment in fixed assets; the investment in the secondary industry increased by 5.8%, accounting for 16.7% of the province's total investment in fixed assets; Investment in the tertiary industry increased by 23.3%, accounting for 80.8% of the province's fixed assets investment. Infrastructure investment was 675 billion 729 million yuan, an increase of 25.5% over the previous year. The investment in high-tech industry was 26.387 billion yuan, an increase of 58.6% over the previous year. Investment in the six energy-intensive industries was 70.543 billion yuan, down 3.2% from the previous year.
Figure 6 The proportion of three industries investment in fixed assets investment in 2017
　　
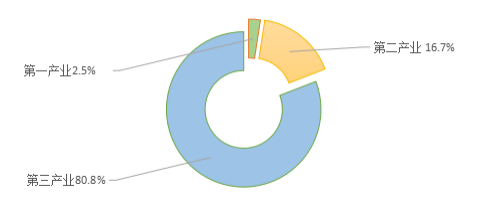

Table 8 Investment in Fixed Assets by Industry and Its Growth Rate in 2017

| Indicator name | Absolute number (100 million yuan) | Year-on-year growth (%) |
| --- | --- | --- |
| Investment in fixed assets |  |  |
| # Coal mining and washing industry | 294.68 | -8.7 |
| Wine, beverage and refined tea manufacturing | 169.97 | -9.9 |
| Chemical raw materials and chemical products manufacturing industry | 114.12 | -9.1 |
| Pharmaceutical manufacturing | 70.34 | 14.4 |
| Ferrous metal smelting and calendering industry | 43.37 | 7.4 |
| Non-ferrous metal smelting and calendering industry | 64.70 | 11.6 |
| Production and supply of electricity, heat, gas and water | 488.42 | 1.8 |
| Transportation, storage and postal services | 2362.30 | 7.7 |
| Information transmission, software and information technology services | 180.89 | 69.4 |
| Scientific research and technical services | 59.35 | 40.3 |
| Management of water conservancy, environment and public facilities | 3908.22 | 44.5 |
| Education | 428.13 | 49.4 |
| Health and Social Work | 189.42 | 79.1 |

The annual investment in real estate development was 220.100 billion yuan, an increase of 2.4% over the previous year. Among them, residential investment was 136 billion 533 million yuan, an increase of 9.8%. Housing construction area is 20385 43 thousand square meters, an increase of 0.2% over the previous year. The completed area was 11.717 million square meters, down 38.4% from the previous year. The sales area was 46.969 million square meters, an increase of 13.0% over the previous year. In the whole year, 432300 new housing units in shantytowns were renovated.
Table 9 Main indicators and growth rate of real estate development and sales in 2017

| Indicator name | Absolute number | Year-on-year growth (%) |
| --- | --- | --- |
| Real estate development investment (100 million yuan) (100 million yuan) | 2201.00 | 2.4 |
| # Residential | 1365.33 | 9.8 |
| Housing construction area (10,000 square meters) | 20385.43 | 0.2 |
| # Residential | 12789.65 | -0.4 |
| Completed housing area (10,000 square meters) | 1171.70 | -38.4 |
| # Residential | 785.00 | -38.8 |
| Housing sales area (10,000 square meters) | 4696.90 | 13.0 |
| # Residential | 3897.65 | 13.7 |

VI. Market and Price
In 2001, the total retail sales of consumer goods was 415.40 billion yuan, up by 12.0% over the previous year. According to the statistics of consumption types, the catering income was 36.497 billion yuan, an increase of 14.7% over the previous year; the retail sales of commodities was 378.902 billion yuan, an increase of 11.7%. According to the statistics of business places, the retail sales of consumer goods in cities and towns was 338 billion 854 million yuan, an increase of 11.8% over the previous year. The retail sales of rural consumer goods was 76.546 billion yuan, up by 12.9%.
Figure 7 Total retail sales of consumer goods and its growth rate from 2013 to 2017
　　
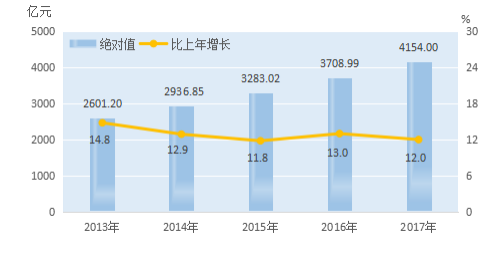

The retail sales of commodities by units above designated size was 214.730 billion yuan, up by 12.5% over the previous year. Among them, the retail sales of grain, oil and food were 15.028 billion yuan, an increase of 16.0%; the retail sales of clothing, shoes and hats, needles and textiles were 8.362 billion yuan, an increase of 6.9%; the retail sales of daily necessities were 5.357 billion yuan, an increase of 5.7%; the retail sales of household appliances and audio-visual equipment were 7.833 billion yuan, an increase of 7.0%; The retail sales of communication equipment was 1.689 billion yuan, an increase of 10.7%; the retail sales of construction and decoration materials was 1.263 billion yuan, an increase of 14.8%; The retail sales of automobiles was 73 billion 420 million yuan, an increase of 9.4%. The retail sales of enterprises (units) above designated size through public networks reached 7.456 billion yuan, an increase of 30.3% over the previous year.
Consumer prices for the whole year rose by 0.9% over the previous year. The ex-factory price of industrial producers rose by 7.2% over the previous year, and the purchasing price of industrial producers rose by 9.7%. The price of investment in fixed assets rose by 6.1% over the previous year, and the price of construction and installation projects rose by 7.3%.
VII. Foreign Economy
The total import and export volume of the year was 8.128 billion US dollars, an increase of 42.6% over the previous year. Of this, export was US $5.777 billion, up 21.8% over the previous year, and import was US $2.351 billion, up 145.8%. In the export market, exports to Hong Kong, Russia, the European Union and Latin America increased by 169.8%, 24.0%, 10.5% and 8.1% respectively, while exports to the United States and South Korea decreased by 20.0% and 48.2% respectively. Among the main export commodities, mechanical and electrical products were 3.220 billion US dollars, up 76.5% over the previous year; high-tech products were 2.521 billion US dollars, up 155.4%; flue-cured tobacco was 118 million US dollars, up 10.3%; Tea was 25 million US dollars, an increase of 24.6%.
Table 10 Total import and export volume and its growth rate in 2017

| Indicator name | Absolute ( $100 million) | Year-on-year growth (%) |
| --- | --- | --- |
| Total imports and exports | 81.28 | 42.6 |
| Total imports | 23.51 | 145.8 |
| # General trade | 8.91 | 60.9 |
| Processing trade | 12.18 | 344.9 |
| Total exports | 57.77 | 21.8 |
| # General trade | 40.75 | -0.7 |
| Processing trade | 16.11 | 195.4 |

The total amount of foreign capital actually utilized in the whole year was 3.891 billion US dollars, an increase of 21.0% over the previous year. The turnover of foreign economic and technological cooperation was 1.1 billion US dollars, an increase of 13.3%. Switzerland (Guizhou) Industrial Demonstration Park started construction and 10 projects were signed and landed. Six new commercial representative offices have been set up in Switzerland, Cambodia, India, Malaysia, Italy and Kyrgyzstan along the "The Belt and Road Initiative".
VIII. Tourism Development
The total number of tourists in the whole year was 74417 43 thousand, an increase of 40.0% over the previous year. Total tourism revenue was 711.681 billion yuan, an increase of 41.6%.
Figure 8 Total tourism revenue and its growth rate, 2013-2017
　　
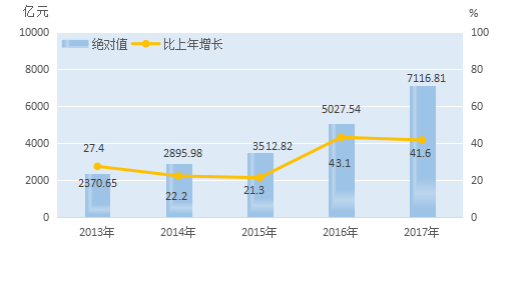

At the end of the year, there were 71 scenic spots. Among them, there are 18 national scenic spots and 53 provincial scenic spots. Five 5A tourist attractions, an increase of one over the end of last year; There are 95 4A-level tourist attractions, an increase of 27 over the end of last year. 131 provincial rural tourism demonstration zones; There are 1104 key villages for poverty alleviation through rural tourism.
IX. Transportation
At the end of the year, 194,400 kilometers of highways were open to traffic, an increase of 1.5% over the end of the previous year. The mileage of expressways open to traffic was 5834.50 kilometers, an increase of 7.4%.
The 94626 volume of railway, highway and waterway freight transport in the whole year was 81000 tons, an increase of 7.9% over the previous year. Of this, highway freight transport accounted for 89298 million tons, up by 8.6%, while railway freight transport accounted for 36.6423 million tons, down by 3.0%. Waterborne cargo transport totaled 16.6457 million tons, up 0.6%.
Table 11 Cargo transportation volume completed by various modes of transport and its growth rate in 2017

| Indicator name | Absolute number | Year-on-year growth (%) |
| --- | --- | --- |
| Total volume of goods transported (10,000 tons) | 94626.81 | 7.9 |
| Railway | 3664.23 | -3.0 |
| Highway | 89298.00 | 8.6 |
| Water transport | 1664.57 | 0.6 |
| Cargo turnover (100 million ton-kilometers) | 1544.52 | 12.2 |
| Railway | 490.87 | 6.5 |
| Highway | 1008.58 | 15.5 |
| Water transport | 45.07 | 6.4 |
| Cargo and mail throughput of civil aviation (10,000 tons) | 10.70 | 8.5 |

The total volume of railway, highway and waterway passenger transport in the whole year was 91465 8600, an increase of 2.7% over the previous year. Of this, the total passenger transport volume of highways was 83809 million, up by 2.0%, and that of railways was 54.5893 million, up by 15.5%. Waterborne passenger transport totaled 21.9793 million, an increase of 4.9%.
Table 12 Passenger traffic volume completed by various modes of transport and its growth rate in 2017

| Indicator name | Absolute number | Year-on-year growth (%) |
| --- | --- | --- |
| Total volume of passenger transport (10,000 persons) | 91465.86 | 2.7 |
| Railway | 5458.93 | 15.5 |
| Highway | 83809.00 | 2.0 |
| Water transport | 2197.93 | 4.9 |
| Passenger transport turnover (100 million person-km) | 704.32 | 7.7 |
| Railway | 233.66 | 13.9 |
| Highway | 463.93 | 4.7 |
| Water transport | 6.72 | 16.8 |
| Civil aviation passenger throughput (10,000 person-times) | 2457.65 | 31.2 |

At the end of the year, there were 6.961 million motor vehicles, an increase of 11.2% over the previous year. Among them, the number of civilian automobiles was 4.1571 million, an increase of 18.6%. Of the civilian automobiles, 1,892,700 were sedans, up 19.9%.
X. Big Data and Posts and Telecommunications
At the end of the year, the provincial Internet bandwidth was 6,730 Gbps, the length of optical cable lines was 900,000 kilometers, and the total number of Internet users was 35.0571 million.
In 2001, the total volume of postal services was 5.323 billion yuan, up by 24.7% over the previous year; The total volume of telecommunications business was 82 billion 529 million yuan, an increase of 146.2%. At the end of the year, the number of mobile phone users was 37.9227 million, an increase of 16.2% over the end of the previous year.
The total volume of express business in the whole year was 15781. 9 million pieces, an increase of 40.2% over the previous year. Express business revenue was 3.115 billion yuan, an increase of 43.0% over the previous year.
Figure 9 Total volume of express business from 2013 to 2017
　　
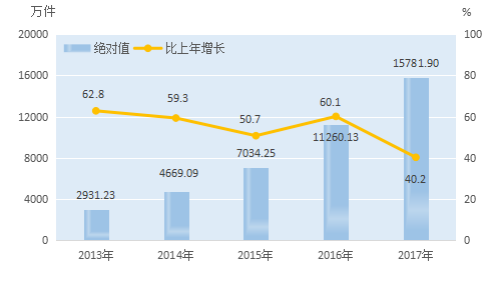

Xi. Finance and banking
The total fiscal revenue of the year was 265.002 billion yuan, an increase of 10.1% over the previous year. The general public budget revenue was 161 billion 364 million yuan, an increase of 7.2% over the previous year. Among them, tax revenue was 117 billion 955 million yuan, an increase of 10.9%.
Table 13 Main Indicators of Fiscal Revenue and Its Growth Rate in 2017

| Indicator name | Absolute number (100 million yuan) | Year-on-year growth (%) |
| --- | --- | --- |
| Total fiscal revenue | 2650.02 | 10.1 |
| # General public budget revenue | 1613.64 | 7.2 |
| # Tax revenues | 1179.55 | 10.9 |
| # Domestic VAT (including VAT) | 415.27 | 9.3 |
| Corporate income tax | 146.57 | 15.3 |
| Personal income tax | 48.53 | 38.4 |
| Urban maintenance and construction tax | 67.26 | 8.6 |
| Deed tax | 89.29 | -2.9 |
| Non-tax revenue | 434.09 | -1.5 |
| # Operating income of state-owned capital | 25.32 | -41.3 |

The annual general public budget expenditure was 460.457 billion yuan, an increase of 8.0% over the previous year. Of this, the expenditure on education was 90.351 billion yuan, an increase of 7.1% over the previous year, and the expenditure on agriculture, forestry and water resources was 60.126 billion yuan, a decrease of 4.5% over the previous year. Expenditure on social security and employment was 50.019 billion yuan, an increase of 36.2% over the previous year.
Table 14 Main Indicators of Fiscal Expenditure and Its Growth Rate in 2017

| Indicator name | Absolute number (100 million yuan) | Year-on-year growth (%) |
| --- | --- | --- |
| General public budget expenditure | 4604.57 | 8.0 |
| # Expenditure on general public services | 473.61 | 6.2 |
| Expenditure on education | 903.51 | 7.1 |
| Expenditure on science and technology | 88.29 | 27.4 |
| Expenditure on culture, sports and media | 64.51 | -4.2 |
| Expenditure on social security and employment | 500.19 | 36.2 |
| Expenditure on health care and family planning | 439.49 | 12.0 |
| Expenditure on energy conservation and environmental protection | 129.52 | 1.9 |
| Expenditure on agriculture, forestry and water | 601.26 | -4.5 |
| Transportation expenditure | 329.52 | 13.6 |
| Housing security expenditure | 244.58 | -16.2 |

At the end of the year, the balance of all kinds of RMB deposits in financial institutions was 26088 1.89 billion yuan, an increase of 231.809 billion yuan over the beginning of the year, an increase of 9.8% over the end of last year. Among them, the 26083 of domestic deposits was 1.16 billion yuan, an increase of 231 billion 788 million yuan over the beginning of the year. Household deposits amounted to 958.029 billion yuan, an increase of 104.769 billion yuan over the beginning of the year.
At the end of the year, the balance of various RMB loans of financial institutions was 20860 1.34 billion yuan, an increase of 300.254 billion yuan or 16.8% over the beginning of the year. Among them, the balance of agriculture-related loans was 874.48 billion yuan, an increase of 162.70 billion yuan over the beginning of the year; the balance of poverty alleviation loans was 393.08 billion yuan, an increase of 131.75 billion yuan over the beginning of the year; The loan balance of small and micro enterprises was 442 billion 310 million yuan, an increase of 98 billion 470 million yuan over the beginning of the year.
Table 15 Balance of RMB deposits and loans of financial institutions at the end of 2017 and its growth rate

| Indicator name | Absolute number (100 million yuan) | Increase over the end of last year (%) |
| --- | --- | --- |
| Balance of various deposits | 26088.89 | 9.8 |
| # Domestic deposits | 26083.16 | 9.8 |
| Household deposits | 9580.29 | 12.3 |
| Deposits of non-financial enterprises | 10059.57 | 8.2 |
| Balance of various loans | 20860.34 | 16.8 |
| # Domestic loans | 20859.62 | 16.8 |
| Household loans | 6200.63 | 19.0 |
| Short-term loan | 1527.82 | 23.9 |
| Medium and long-term loans | 4672.80 | 17.4 |
| Loans to non-financial enterprises and institutions | 14658.77 | 15.9 |
| Short-term loan | 2263.79 | 3.2 |
| Medium and long-term loans | 12125.57 | 19.6 |

The direct financing of stocks and bonds in the whole year was 14.940 billion yuan, down 82.5% from the previous year. At the end of the year, there were 27 domestic listed companies, an increase of 4 over the end of the previous year. The transaction value of securities bought and sold by securities companies as agents was 863.498 trillion yuan, an increase of 4.1% over the previous year.
In 2001, the premium income of insurance companies totaled 38.773 billion yuan, up by 20.7% over the previous year. Among them, property insurance premium income was 17 billion 926 million yuan, an increase of 17.1%; Life insurance premium income was 20.847 billion yuan, an increase of 24.0%. Indemnities and payments amounted to 15.381 billion yuan, up 16.9%. The premium income of agricultural insurance was 826 million yuan, an increase of 24.8%, providing 107.695 billion yuan of risk protection for 6.9791 million households.
Table 16 Main Indicators and Growth Rate of Insurance Industry in 2017

| Indicator name | Absolute number (100 million yuan) | Year-on-year growth (%) |
| --- | --- | --- |
| Original premium income | 387.73 | 20.7 |
| # Property insurance | 179.26 | 17.1 |
| # Motor Vehicle Insurance | 146.96 | 15.3 |
| Personal insurance | 208.47 | 24.0 |
| Accident Insurance | 14.22 | 23.6 |
| Health insurance | 37.65 | 35.2 |
| Life Insurance | 156.60 | 21.6 |
| Compensation expenses | 153.81 | 16.9 |
| # Property insurance | 91.38 | 15.0 |
| # Motor Vehicle Insurance | 77.00 | 14.5 |
| Personal insurance | 62.43 | 19.9 |
| Accident Insurance | 4.78 | 22.8 |
| Health insurance | 19.68 | 53.6 |
| Life Insurance | 37.97 | 7.4 |

XII. People's livelihood
The annual per capita disposable income of all residents was 16704 yuan, a nominal increase of 10.5% over the previous year. According to the permanent residence, the per capita disposable income of urban residents was 29080 yuan, an increase of 8.7% over the previous year. The per capita disposable income of rural residents was 8869 yuan, a nominal increase of 9.6% over the previous year.
Table 17 Per capita disposable income of urban and rural residents and its growth rate in 2017

| Indicator name | Absolute number (yuan) | Year-on-year growth (%) |
| --- | --- | --- |
| Per capita disposable income of urban residents | 29080 | 8.7 |
| # Wage income | 16552.58 | 7.8 |
| Net income from operations | 4721.76 | 10.3 |
| Net income from property | 2184.87 | 12.6 |
| Net income transferred | 5620.63 | 8.8 |
| Per capita disposable income of rural residents | 8869 | 9.6 |
| # Wage income | 3635.67 | 13.2 |
| Net income from operations | 3285.17 | 5.4 |
| Net income from property | 92.03 | 37.2 |
| Net income transferred | 1856.24 | 9.4 |

The per capita consumption expenditure of all residents was 12970 yuan, an increase of 8.7% over the previous year. By permanent residence, the per capita consumption expenditure of urban residents was 203.48 yuan, an increase of 6.0% over the previous year. The per capita consumption expenditure of rural residents was 8299 yuan, an increase of 10.2%.
Figure 10 Per capita consumption expenditure and its composition of urban residents in the province in 2017
　　
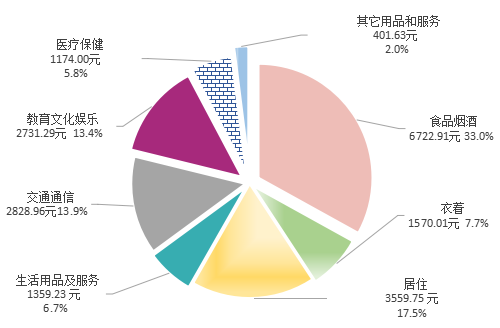

Figure 11 Per capita consumption expenditure and its composition of rural residents in the province in 2017
　　
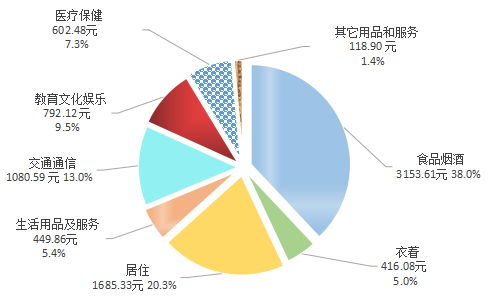

The per capita housing construction area of urban residents is 37.52 square meters, and the per capita housing construction area of rural residents is 34.54 square meters.
At the end of the year, every 100 urban households owned 35.0 household automobiles, 34.5 air conditioners and 256.7 mobile phones, up 4.3%, 11.8% and 3.7%, respectively, as compared with the end of the previous year. The number of refrigerators per 100 rural households was 82.3, up 6.1%, and the number of motorcycles per 100 rural households was 54.2, down 2.7%. There were 255.5 mobile phones, an increase of 6.2%.
At the end of the year, the number of urban employees participating in basic old-age insurance was 5.8817 million, an increase of 1.6459 million over the end of the previous year, of which 3.3782 million were enterprise employees, an increase of 279.5 million. The number of urban and rural residents participating in basic old-age insurance was 17.4855 million, an increase of 454700 over the end of last year. The number of people participating in unemployment insurance was 2 million 357 thousand and 100, an increase of 176 thousand and 100 over the end of last year. The number of employees participating in basic medical insurance was 4.1042 million, an increase of 206100 over the end of last year. The number of urban and rural residents participating in basic medical insurance was 5.9092 million, an increase of 71400 over the end of last year. The number of people participating in industrial injury insurance was 3.3248 million, an increase of 274600 over the end of last year, of which 928800 were migrant workers, an increase of 55100. The number of people participating in maternity insurance was 3.0403 million, an increase of 177600 over the end of last year.
At the end of the year, the number of urban minimum living security was 314400, and the annual per capita security standard was 6732 yuan, an increase of 612 yuan over the previous year. At the end of the year, the number of rural minimum living security people was 2.6093 million, and the annual per capita security standard was 3580 yuan, an increase of 396 yuan over the previous year.
At the end of the year, there were 24,095 old-age service institutions and facilities, and 173,600 old-age beds. There are 47 adoption and rescue agencies, and the number of adoption and rescue personnel is 37399. The annual sales of social welfare lottery tickets amounted to 2.823 billion yuan, an increase of 4.9% over the previous year. Social welfare funds raised 884 million yuan, an increase of 3.6%.
XIII. Scientific and Technological Innovation
At the end of the year, there were one national intellectual property demonstration park, two national international scientific and technological cooperation bases, 73 academician workstations and five national key laboratories. 144 scientific and technological achievements at or above the provincial and ministerial levels were made in the whole year, an increase of 20.0% over the previous year. Among them, there were 36 achievements in basic theory, an increase of 28.6% over the previous year, and 108 achievements in applied technology, an increase of 17.4% over the previous year. 2,957 technology contracts were signed, up by 201.7% over the previous year; The turnover was 8.384 billion yuan, an increase of 274.5%.
It has won two National Science and Technology Progress Awards throughout the year. The number of applications for invention patents was 13884, up 26.8% over the previous year. The number of invention patents authorized was 1875, down 7.9%. The number of valid invention patents per 10000 people was 2.37, an increase of 19.1% over the previous year.
The Provincial Governor's Quality Award was established, and 8 enterprises were awarded the Provincial Governor's Quality Award and nominated for the award. One city with strong quality, three national famous brand demonstration zones, 12 provincial famous brand demonstration zones and 692 provincial famous brand products have been established. There are 13 national organic product certification demonstration zones, 1191 organic product certification certificates, 6355 pollution-free agricultural products producing areas and 2850 products. Leading the formulation of 1 international standards for steel cable.
XIV. Education, Culture, Health and Sports
Guizhou University was selected as the national "double first-class" discipline construction university. In the whole year, 836 new and expanded primary and secondary schools, 30 urban compulsory education schools, 900 kindergartens and 112 ordinary high schools were completed.
Postgraduate education enrolled 7,100 students, with 18,600 students enrolled; regular undergraduate and junior college students enrolled 209,500 students, with 627,700 students enrolled; regular senior middle schools enrolled 348,500 students, with 1,011,000 students enrolled; secondary vocational education enrolled 183,100 students, with 503,100 students enrolled; There were 1,829,900 students enrolled in junior middle schools, 650,500 students enrolled in regular primary schools, and 25,800 students enrolled in special education. 738000 children were enrolled in preschool education, and 1534200 children were in kindergartens. The popularization rate of nine-year compulsory education is 90%, and the gross enrollment rate of senior high school is 87.0%.
Figure 12 Number of students enrolled in general colleges, secondary vocational education and general high schools from 2013 to 2017
　　
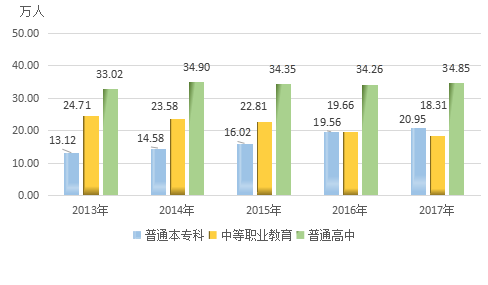

At the end of the year, the comprehensive population coverage rate of radio was 93.5%, and that of television was 96.5%. There are 1.3364 million new users of "Radio and Television Cloud" in Guizhou, and 302 new comprehensive service stations in villages and towns. It has 181 cinemas operating in the city, with a box office revenue of 624 million yuan. It publishes 1050 kinds of books, 39 kinds of newspapers and 93 kinds of magazines. There are 42 art performance groups, 6 art performance venues, 73 museums and memorials, 98 public libraries, 99 mass art galleries and cultural centers, and 1567 comprehensive cultural stations in towns and townships. There are 71 key cultural relics protection units in China.
At the end of the year, there were 28,100 medical and health institutions, including 2,700 hospitals and health centers. There are 362 professional public health institutions, including 100 centers for disease control and prevention. The number of beds in hospitals and health centers was 221,100, an increase of 10.6% over the end of last year.
Athletes won 60 medals in major international and domestic sports competitions throughout the year, an increase of 18 medals over the previous year, an increase of 42.9%. Among them, one world champion and 19 gold medals in the highest level competitions in China were won, an increase of 46.2% over the previous year. There were 117 stadiums and gymnasiums, an increase of 9 or 8.0% over the previous year. 551 sets of national fitness equipment were installed, an increase of 1.7% over the previous year.
Fifteenth, ecological environment and energy saving and consumption reduction
At the end of the year, the forest coverage rate was 55.3%, and the afforestation area was 10 million mu. It has 10 national nature reserves, 30 national forest parks, 44 provincial forest parks and 1 municipal forest park. There are 45 national wetland parks, 4 provincial wetland parks and 2 wetland nature reserves.
The average concentrations of fine particulate matter (PM2.5), inhalable particulate matter (PM10), sulfur dioxide (SO2) and nitrogen dioxide (NO2) in the air of nine central cities in the whole year decreased by 9.4%, 5.7%, 13.3% and 4.5% respectively over the previous year. The proportion of days with good air quality in 9 central cities is 96.5%. The average concentration of chemical oxygen demand (COD) in the provincial control sections of major rivers (151 sections) decreased by 10.4% over the previous year, and the average concentration of ammonia nitrogen decreased by 3.4%. The standard rate of centralized drinking water quality in 9 central cities is 100%.
Renewable energy generated 81.245 billion kWh in the whole year, an increase of 3.3% over the previous year, accounting for 40.4% of the total power generation. Among them, hydropower generation was 73.479 billion kWh, an increase of 1.0%; wind power generation was 6.389 billion kWh, an increase of 20.3%; solar power generation was 750 million kWh, an increase of 456.7%; Biomass and garbage generated 627 million kwh, an increase of 37.8%. The energy consumption of 10000 yuan GDP decreased by 7.0% compared with the previous year, and the energy consumption of 10000 yuan added value of industries above scale decreased by 8.53%. The proportion of the added value of the "four-type" industries in the GDP of the green economy has increased to 37.0%.
Note:
1. The data in this bulletin are preliminary statistics.
2. The growth rate of GDP, industrial added value above scale and its classified items is calculated at comparable prices, which is the actual growth rate; Unless otherwise specified, other indicators are nominal growth rates calculated at current prices.
3. Gross regional product refers to the total value of all final products and services produced by all resident units in a region in a certain period of time.
4. According to the requirements of the Poverty Alleviation Office of the State Council, the population base for calculating the incidence of poverty is the number of agricultural household registration population of the Ministry of Public Security in 2014. In 2017, according to the unified deployment of the dynamic adjustment of the national archives, the province cleared the farmers who did not meet the poverty alleviation standards, supplemented the registration of the poor households who met the poverty alleviation standards, and the population increased or decreased. Therefore, at the end of 2016, 3.722 million poor people and 10.6% of the incidence of poverty were not closed.
5. The statistical scope of investment in fixed assets covers investment in fixed assets projects with a total planned investment of more than 5 million yuan and investment in all real estate development projects. Investment in fixed assets refers to the general term of the workload of construction and purchase of fixed assets completed in a certain period of time in monetary form and the related expenses.
6. The six energy-intensive industries include petroleum processing, coking and nuclear fuel processing, chemical raw materials and chemical products manufacturing, non-metallic mineral products, ferrous metal smelting and calendering, non-ferrous metal smelting and calendering, power and heat production and supply.
7. The statistical scope of industries above designated size is industrial enterprises with annual main business income of 20 million yuan or more.
8. Units above the quota in the statistics of total retail sales of social consumer goods refer to wholesale enterprises (units) with annual main business income of 20 million yuan or more, retail enterprises (units) with annual main business income of 5 million yuan or more, accommodation and catering enterprises (units) with annual main business income of 2 million yuan or more.
9. Online retail sales refer to the sum of retail sales of goods and services through public online trading platforms (including self-built websites and third-party platforms). Goods and services include physical goods and non-physical goods (such as virtual goods, service goods, etc.).
Total retail sales of consumer goods include online retail sales of physical goods, excluding online retail sales of non-physical goods.
10. Consumer price index refers to the relative number reflecting the trend and degree of changes in the prices of consumer goods and services purchased by urban and rural residents in a certain period of time.
11. Due to the rounding of some data, there is a difference between the total and the sub-total.

[[Back to top]](javascript:scroll(0,0)) [[Print this page]](javascript:window.print();) [[Close this page]](javascript:window.close())

Previous:[Statistical Bulletin on the National Economic and Social Development of Guizhou Province in 2017](http://hgk.guizhou.gov.cn/publish/articles/c7/2023/09/a412/a412.html?locationhref=http://hgk.guizhou.gov.cn/publish/channels/c7/c7_1psSuffix&pagesize=15&curpage=1&curainum=14)
Next:[Statistical Communique on the National Economic and Social Development of Guizhou Province in 2016](http://hgk.guizhou.gov.cn/publish/articles/c7/2023/09/a405/a405.html?locationhref=http://hgk.guizhou.gov.cn/publish/channels/c7/c7_1psSuffix&pagesize=15&curpage=2&curainum=1)

Sponsor: Guizhou Provincial Bureau of Statistics Technical Support: Guizhou Jiawang Technology Development Co., Ltd.

Record No.: Qian ICP 19000889 No.: -3

## Statistical Communique on the National Economic and Social Development of Guizhou Province in 2015

## Guizhou Provincial Bureau of Statistics National Bureau of Statistics Guizhou Investigation Team

## (22 March 2016)

## In 2015, facing the complicated economic situation at home and abroad, the whole province thoroughly implemented the spirit of General Secretary 's important speech during his visit to Guizhou, earnestly implemented the decision-making arrangements of the Party Central Committee and the State Council, closely centered on the general requirements of keeping the bottom line, taking a new road and running towards a well-off society, and adhered to accelerating development, accelerating transformation and promoting a new leap forward. In-depth implementation of the main strategy of strengthening the province by industry and urbanization, striving to do a good job in stabilizing growth, promoting reform, adjusting structure, benefiting people's livelihood and preventing risks, successfully completed the main objectives and tasks of economic and social development throughout the year, achieved a successful conclusion of the 12th Five-Year Plan, and laid a solid foundation for the 13th Five-Year Plan to build a well-off society in an all-round way in synchronization with the whole country.

## First, comprehensive

According to the preliminary accounting, in 2015, the gross regional product (GDP) of the whole province exceeded 1 trillion yuan, which was 0.56 billion yuan 10502, an increase of 10.7% over the previous year, and 210.256 billion yuan more than the "Twelfth Five-Year Plan" target (840 billion yuan). During the "Twelfth Five-Year Plan" period, the province's GDP increased by 118 billion 8 million yuan annually, with an average annual growth of 12.5%. The proportion of Guizhou's GDP in the whole country increased from 1.13% in 2010 to 1.55% in 2015.

Of the provincial GDP, the added value of the primary industry was 164.062 billion yuan, up by 6.5% over the previous year; the added value of the secondary industry was 414.694 billion yuan, up by 11.4% over the previous year; The added value of the tertiary industry was 471.5 billion yuan, up by 11.1% over the previous year. The added value of the primary, secondary and tertiary industries accounted for 15.6%, 39.5% and 44.9% of the GDP, respectively. The per capita GDP of the province was 29847 yuan, an increase of 10.3% over the previous year.

| Table 1 GDP of the whole province from 2011 to 2015 | | | | | | |
| --- | --- | --- | --- | --- | --- | --- |
| Unit: 100 million yuan | | | | | | |
| **Indicator name** | **2011** | **2012** | **2013** | **2014** | **2015** | **2015 vs 2014**  **Growth (%)** |
| Gross regional product | 5701.84 | 6852.20 | 8086.86 | 9266.39 | 10502.56 | 10.7 |
| Added value of the primary industry | 726.22 | 891.91 | 998.47 | 1280.45 | 1640.62 | 6.5 |
| Added value of the secondary industry | 2194.33 | 2677.54 | 3276.24 | 3857.44 | 4146.94 | 11.4 |
| Industry | 1829.20 | 2217.06 | 2686.52 | 3140.88 | 3315.58 | 9.8 |
| Construction | 365.13 | 460.48 | 590.69 | 717.69 | 832.55 | 18.3 |
| Added value of the tertiary industry | 2781.29 | 3282.75 | 3812.15 | 4128.50 | 4715.00 | 11.1 |
| Wholesale and retail trade | 448.77 | 514.49 | 582.05 | 624.17 | 671.39 | 8.4 |
| Transportation, storage and postal services | 590.91 | 687.45 | 772.44 | 828.69 | 920.36 | 9.4 |
| Accommodation and catering | 224.40 | 266.58 | 294.86 | 322.71 | 360.38 | 9.7 |
| The financial industry | 297.27 | 365.87 | 444.53 | 491.65 | 607.11 | 19.2 |
| Real estate | 160.30 | 176.75 | 202.94 | 220.48 | 232.07 | 6.6 |
| Other services |  |  |  |  |  |  |
| For-profit service industry | 398.09 | 467.57 | 546.11 | 591.36 | 687.39 | 11.9 |
| Non-profit service industry | 661.55 | 804.04 | 935.02 | 1012.68 | 1163.07 | 11.5 |
| Per capita GDP (yuan) | 16413 | 19710 | 23151 | 26437 | 29847 | 10.3 |
| Note: 1. The data of 2013 is revised according to the data of the third economic census; 2. According to the unified requirements of the National Bureau of Statistics, the national economic accounting data for 2013-2014 adopt the Classification of National Economic Industries (GB/T4754-2011) and the Regulations on the Classification of Three Industries (Guo Tong Zi [2012] No.108). Since 2013, the added value of the primary industry is equal to the sum of the added value of agriculture, forestry, animal husbandry and fishery, excluding the service industry of agriculture, forestry, animal husbandry and fishery; the added value of the secondary industry is equal to the sum of the added value of industry and construction, minus the added value of mining auxiliary activities in industry and the added value of metal product machinery and equipment repair industry; Agriculture, forestry, animal husbandry and fishery services, mining auxiliary activities, metal products, machinery and equipment repair industry are classified into the tertiary industry. | | | | | | |

## II. Agriculture

In 2001, the added value of agriculture, forestry, animal husbandry and fishery was 171.266 billion yuan, up by 6.4% over the previous year. Among them, the added value of planting industry was 109.654 billion yuan, an increase of 7.8%; the added value of forestry was 9.287 billion yuan, an increase of 7.9%; the added value of animal husbandry was 41.594 billion yuan, an increase of 1.2%; the added value of fishery was 3.526 billion yuan, an increase of 17.1%; The added value of agriculture, forestry, animal husbandry and fishery services was 7.204 billion yuan, an increase of 4.6%.

| Table 2 The added value of agriculture, forestry, animal husbandry and fishery in the whole province from 2011 to 2015 | | | | | | |
| --- | --- | --- | --- | --- | --- | --- |
| Unit: 100 million yuan | | | | | | |
| **Indicator name** | **2011** | **2012** | **2013** | **2014** | **2015** | **2015 vs 2014**  **Growth (%)** |
| Added value of agriculture, forestry, animal husbandry and fishery | 726.21 | 891.91 | 1031.70 | 1316.08 | 1712.66 | 6.4 |
| Planting | 430.84 | 561.32 | 646.12 | 851.89 | 1096.54 | 7.8 |
| Forestry | 31.99 | 37.03 | 47.71 | 68.15 | 92.87 | 7.9 |
| Animal husbandry | 223.22 | 245.7 | 280.68 | 331.16 | 415.94 | 1.2 |
| Fishery | 12.78 | 17.83 | 23.96 | 29.25 | 35.26 | 17.1 |
| Agriculture, forestry, animal husbandry and fishery services | 27.38 | 30.03 | 33.23 | 35.63 | 72.04 | 4.6 |

The planting area of grain crops was 3,114.91 thousand hectares, a decrease of 0.8% over the previous year; the planting area of oil crops was 591.85 thousand hectares, an increase of 1.7% over the previous year; the planting area of flue-cured tobacco was 181.67 thousand hectares, a decrease of 16.1% over the previous year; the planting area of vegetables was 996.29 thousand hectares, an increase of 7.8% over the previous year; The planting area of traditional Chinese medicinal materials was 155.81 thousand hectares, an increase of 6.3% over the previous year; the actual area of tea plantations at the end of the year was 418.89 thousand hectares, an increase of 13.5% over the previous year; At the end of the year, the orchard area was 299.89 thousand hectares, an increase of 14.4% over the previous year. The total grain output was 11.8 million tons, an increase of 3.7% over the previous year, the highest level in history, including 2.6945 million tons of summer grain and 9.1055 million tons of autumn grain. The output of tea, fruits, vegetables and traditional Chinese medicines increased by 35.6%, 15.7%, 11.1% and 10.7% respectively over the previous year.

| Table 3 Output of Main Agricultural Products in the Province from 2011 to 2015 | | | | | | |
| --- | --- | --- | --- | --- | --- | --- |
|  |  |  |  |  |  | Unit: 10,000 tons |
| **Indicator name** | **2011** | **2012** | **2013** | **2014** | **2015** | **2015 vs 2014**  **Growth (%)** |
| Food crops | 876.90 | 1079.50 | 1029.99 | 1138.50 | 1180.00 | 3.7 |
| ^#^ Rice | 303.93 | 402.43 | 361.30 | 403.24 | 417.54 | 3.6 |
| Wheat | 50.38 | 52.39 | 51.51 | 61.50 | 61.67 | 0.3 |
| Corn | 243.71 | 342.25 | 298.03 | 313.81 | 324.08 | 3.3 |
| ^#^ Potato | 189.38 | 179.74 | 211.40 | 226.60 | 237.62 | 4.9 |
| Oil crops | 78.85 | 87.38 | 91.53 | 98.05 | 101.55 | 3.6 |
| ^#^ Rapeseed | 71.81 | 78.18 | 81.78 | 86.69 | 89.02 | 2.7 |
| Peanut | 6.07 | 7.86 | 8.25 | 9.71 | 10.71 | 11.3 |
| Flue-cured tobacco | 32.50 | 37.31 | 41.79 | 35.34 | 32.86 | -7.0 |
| Vegetables | 1250.05 | 1375.63 | 1500.45 | 1625.62 | 1805.89 | 11.1 |
| Sugarcane | 43.60 | 127.96 | 159.29 | 168.27 | 156.09 | -7.2 |
| Chinese herbal medicine | 8.36 | 14.06 | 24.80 | 36.06 | 39.91 | 10.7 |
| Tea | 5.84 | 7.44 | 8.94 | 10.71 | 14.53 | 35.6 |
| Fruit | 128.03 | 147.72 | 167.75 | 196.38 | 227.17 | 15.7 |

The annual output of pork was 1,607,500 tons, down by 2.9% over the previous year; the output of beef was 167,600 tons, up by 14.2% over the previous year; the output of mutton was 42,000 tons, up by 12.0% over the previous year; the output of poultry was 163,100 tons, up by 9.9% over the previous year; The output of other meat was 39,200 tons, up by 31.5% over the previous year. Milk output was 62 thousand tons, an increase of 8.6% over the previous year. The output of poultry eggs was 173 thousand and 300 tons, an increase of 7% over the previous year. At the end of the year, 17.9526 million pigs were slaughtered and 15.5896 million pigs were kept in stock, down 2.7% and 2.6% respectively from the previous year. 96.1819 million poultry were sold and 84.0278 million were kept in stock, up 5.0% and 5.9% respectively over the previous year.

| Table 4 Livestock and Poultry Production in the Province from 2011 to 2015 | | | | | | |
| --- | --- | --- | --- | --- | --- | --- |
| **Indicator name** | **2011** | **2012** | **2013** | **2014** | **2015** | **2015 vs 2014**  **Growth (%)** |
| Number of sales in the current year |  |  |  |  |  |  |
| Pigs (10,000) | 1689.66 | 1734.76 | 1832.28 | 1845.27 | 1795.26 | -2.7 |
| Cattle (10,000) | 97.21 | 105.99 | 115.22 | 117.35 | 133.26 | 13.6 |
| Sheep (10,000) | 197.31 | 206.78 | 205.39 | 220.38 | 246.14 | 11.7 |
| Birds (ten thousand feathers) | 8878.57 | 9632.03 | 9681.62 | 9162.14 | 9618.19 | 5.0 |
| Number on hand at the end of the year |  |  |  |  |  |  |
| Pigs (10,000) | 1521.60 | 1604.09 | 1604.10 | 1600.57 | 1558.96 | -2.6 |
| Cattle (10,000) | 467.11 | 461.04 | 460.62 | 495.86 | 535.95 | 8.1 |
| Sheep (10,000) | 256.49 | 290.09 | 299.59 | 337.40 | 354.67 | 5.1 |
| Birds (ten thousand feathers) | 7698.14 | 8355.25 | 8154.72 | 7932.05 | 8402.78 | 5.9 |
| Output of animal products |  |  |  |  |  |  |
| Pork (10,000 tons) | 148.29 | 156.13 | 163.73 | 165.55 | 160.75 | -2.9 |
| Beef (10,000 tons) | 12.00 | 13.04 | 14.13 | 14.68 | 16.76 | 14.2 |
| Mutton (10,000 tons) | 3.37 | 3.53 | 3.51 | 3.75 | 4.20 | 12.0 |
| Poultry meat (10,000 tons) | 14.35 | 15.41 | 15.48 | 14.84 | 16.31 | 9.9 |
| Milk (10,000 tons) | 4.85 | 5.10 | 5.45 | 5.71 | 6.20 | 8.6 |
| Eggs (10,000 tons) | 13.65 | 14.65 | 15.44 | 16.20 | 17.33 | 7.0 |
| Honey (tons) | 2029 | 2052 | 2468 | 2733 | 3017 | 10.4 |
|  | | | | | | |

## III. Industry and Construction

The added value of industries above designated size in the whole year was 355.013 billion yuan, an increase of 9.9% over the previous year. Among them, the added value of light and heavy industries was 137 billion 419 million yuan and 217 billion 594 million yuan respectively, increasing by 8.3% and 10.9% respectively. During the "Twelfth Five-Year Plan" period, the added value of industries above the provincial scale increased by 14.3% annually.

In the whole year, the four traditional industries of coal, electricity, tobacco and alcohol realized an added value of 206.907 billion yuan, accounting for 58.3% of the industrial added value above the scale. Among them, the added value of wine, beverage and refined tea manufacturing industry was 71.605 billion yuan, an increase of 10.2% over the previous year; the added value of coal mining and washing industry was 68.468 billion yuan, an increase of 5.6%; the added value of power, heat production and supply industry was 36.453 billion yuan, an increase of 4.2%. The added value of pharmaceutical manufacturing, computer, communication and other electronic equipment manufacturing exceeded 10 billion yuan and 5 billion yuan respectively, up 6.9% and 102.0% respectively over the previous year. The industrial added value of equipment manufacturing industry and high-tech industry increased by 24.0% and 22.5% respectively over the previous year.

| Table 5 The added value of industries above designated size of the province from 2011 to 2015 | | | | | | |
| --- | --- | --- | --- | --- | --- | --- |
| Unit: 100 million yuan | | | | | | |
| **Indicator name** | **2011** | **2012** | **2013** | **2014** | **2015** | **2015 vs 2014**  **Growth (%)** |
| Industrial added value above designated size | 1638.71 | 2055.46 | 2531.92 | 3117.60 | 3550.13 | 9.9 |
| # Coal mining and washing industry | 411.35 | 472.21 | 558.35 | 676.28 | 684.68 | 5.6 |
| Wine, beverage and refined tea manufacturing | 238.88 | 378.82 | 495.62 | 613.85 | 716.05 | 10.2 |
| Tobacco products industry | 175.81 | 246.89 | 274.74 | 302.19 | 303.81 | -2.3 |
| Chemical raw materials and chemical products manufacturing industry | 96.68 | 109.43 | 124.84 | 141.46 | 159.75 | 9.4 |
| Pharmaceutical manufacturing | 52.02 | 52.70 | 68.45 | 85.53 | 101.63 | 6.9 |
| Non-metallic mineral products industry | 61.41 | 66.34 | 106.79 | 190.56 | 248.63 | 13.0 |
| Ferrous metal smelting and calendering industry | 66.70 | 58.92 | 64.19 | 79.42 | 79.11 | 5.4 |
| Non-ferrous metal smelting and calendering industry | 57.68 | 75.20 | 94.07 | 140.13 | 151.11 | 18.4 |
| Computer, communication and other electronic equipment manufacturing | 10.50 | 14.52 | 20.93 | 20.73 | 52.51 | 102.0 |
| Production and supply of electricity and heat | 234.89 | 295.39 | 318.03 | 340.17 | 364.53 | 4.2 |

In the whole year, 432 new enterprises (excluding growth enterprises) were included in the scope of industrial statistics above the scale, of which 417 were non-public holding industrial enterprises, accounting for 96.5% of the new enterprises included in the scope of industrial statistics above the scale. From the perspective of product distribution, industrial enterprises above the provincial scale produce 282 kinds of industrial products within the statistical scope of 567 kinds, with a product coverage rate of 49.7%.

| Table 6 Output of Major Industrial Products above Designated Size of the Province from 2011 to 2015 | | | | | | |
| --- | --- | --- | --- | --- | --- | --- |
| **Indicator name** | **2011** | **2012** | **2013** | **2014** | **2015** | **2015 vs 2014**  **Growth (%)** |
| Power generation (100 million kWh) | 1359.01 | 1548.44 | 1620.08 | 1682.27 | 1740.92 | 3.3 |
| Phosphate rock (containing 30% of phosphorus pentoxide) (10,000 tons) | 2084.18 | 2281.95 | 2905.44 | 3397.42 | 4323.10 | 15.1 |
| Beverage wine (thousands of liters) | 61.80 | 67.14 | 91.21 | 116.99 | 139.81 | 15.7 |
| # White wine | 25.49 | 26.83 | 30.49 | 38.05 | 42.79 | 11.1 |
| Cigarettes (10,000 cartons) | 245.23 | 249.35 | 254.29 | 258.36 | 252.34 | -2.3 |
| Chinese patent medicine (10,000 tons) | 4.92 | 5.74 | 7.92 | 7.68 | 8.53 | 10.8 |
| Multicolor printed matter (ten thousand folio color order) | 129.57 | 401.14 | 415.62 | 1231.89 | 1469.03 | 19.1 |
| Coke (10,000 tons) | 624.25 | 754.54 | 827.41 | 733.21 | 729.46 | -4.2 |
| Agricultural nitrogen, phosphorus and potassium chemical fertilizers (converted into pure) (10,000 tons) | 360.37 | 503.82 | 524.26 | 533.52 | 582.47 | 8.2 |
| Rubber tire casing (10,000 pieces) | 536.77 | 622.78 | 602.15 | 546.46 | 484.78 | -11.3 |
| Cement (10,000 tons) | 5250.89 | 6100.45 | 8352.95 | 9386.89 | 9909.52 | 5.4 |
| Pig iron (10,000 tons) | 482.35 | 552.93 | 539.22 | 498.64 | 407.58 | -18.2 |
| Steel (10,000 tons) | 462.77 | 560.22 | 573.28 | 552.39 | 463.04 | -16.2 |
| Ferroalloy (10,000 tons) | 263.90 | 309.68 | 327.25 | 348.18 | 332.61 | -4.4 |
| Ten kinds of non-ferrous metals (10,000 tons) | 94.55 | 110.61 | 119.87 | 71.89 | 91.41 | 27.1 |
| Primary aluminum (electrolytic aluminum) (10,000 tons) | 90.36 | 104.35 | 112.28 | 65.21 | 85.52 | 31.1 |
| Household refrigerators (10,000 units) | 169.54 | 159.04 | 155.33 | 168.22 | 174.14 | 3.5 |
| Integrated circuit (10,000 pieces) | 1189.82 | 1558.83 | 1812.71 | 1991.79 | 3079.68 | 51.2 |
| Color TV sets (10,000 sets) | 77.87 | 90.61 | 121.97 | 115.68 | 138.64 | 14.5 |

In 2001, the main business income of industrial enterprises above designated size was 937.620 billion yuan, up by 11.4% over the previous year; The total profit was 61.610 billion yuan, an increase of 10.7% over the previous year.

At the end of the year, there were 892 qualified general contracting enterprises and specialized contracting enterprises in the construction industry, an increase of 6.1% over the end of the previous year. Among them, there are 59 enterprises with Grade I qualification, an increase of 2; and 266 enterprises with Grade II qualification, an increase of 33. In 2001, the total output value of the construction industry was 194.774 billion yuan, up by 18.7% over the previous year. The main business income of construction enterprises was 181.544 billion yuan, an increase of 19.0% over the previous year, and the total profit was 3.908 billion yuan, an increase of 11.4% over the previous year. The total tax was 7.521 billion yuan, an increase of 19.1% over the previous year.

## IV. Investment in fixed assets

In 2001, the 10676 of investment in fixed assets was 0.70 billion yuan, up by 21.6% over the previous year. During the Twelfth Five-Year Plan period, the province completed a total of 330 million yuan of 36089 in fixed assets investment, with an average annual growth of 29.5% from 2012 to 2015.

The investment in infrastructure in the whole year was 413.735 billion yuan, an increase of 22.3% over the previous year, accounting for 38.8% of the province's fixed assets investment. Industrial investment was 274.622 billion yuan, an increase of 17.5% over the previous year, accounting for 25.7% of the province's fixed assets investment. Among them, the manufacturing industry invested 179 billion 567 million yuan, an increase of 18.4%.

| Table 7 Proportion of investment in three major areas in fixed assets investment in the province from 2011 to 2015 | | | | | | |
| --- | --- | --- | --- | --- | --- | --- |
| Unit: 100 million yuan | | | | | | |
| **Year** | **Infrastructure investment** | | **Industrial investment** | | **Investment in real estate development** | |
|  | **Absolute number (100 million yuan)** | **Proportion to investment in fixed assets (%)** | **Absolute number (100 million yuan)** | **Proportion to investment in fixed assets (%)** | **Absolute number (100 million yuan)** | **Proportion to investment in fixed assets (%)** |
| 2011 | 1530.60 | 38.0 | 1332.81 | 33.1 | 873.48 | 21.7 |
| 2012 | 1969.42 | 35.8 | 1614.00 | 29.3 | 1467.60 | 26.7 |
| 2013 | 2587.55 | 36.4 | 1950.07 | 27.5 | 1942.54 | 27.3 |
| 2014 | 3382.19 | 38.5 | 2337.81 | 26.6 | 2187.67 | 24.9 |
| 2015 | 4137.35 | 38.8 | 2746.22 | 25.7 | 2205.09 | 20.7 |

The annual investment in real estate development was 220.509 billion yuan, an increase of 0.8% over the previous year, accounting for 20.7% of the province's fixed assets investment. Housing construction area 20877 67 thousand square meters, an increase of 2.5% over the previous year. Among them, the residential construction area was 13592 65 thousand square meters, down 1.5%. The area of land acquisition was 6.012 million square meters, down 35.8% from the previous year. Commercial housing sales area of 3559.81 square meters, an increase of 12.0% over the previous year; The sales volume of commercial houses was 157.168 billion yuan, an increase of 14.7% over the previous year.

## V. Market and Price

Retail sales of consumer goods totaled 328.302 billion yuan, up by 11.8% over the previous year. According to the location of business units, the retail sales of consumer goods in cities and towns was 269 billion 166 million yuan, an increase of 11.8%. Retail sales of rural consumer goods was 59.136 billion yuan, an increase of 11.7%. During the "Twelfth Five-Year Plan" period, the total retail sales of social consumer goods in the province increased by 17.2% annually.

| Table 8 Total Retail Sales of Social Consumer Goods in the Province from 2011 to 2015 | | | | | | |
| --- | --- | --- | --- | --- | --- | --- |
|  |  |  |  |  | Unit: 100 million yuan | |
| **Indicator name** | **2011** | **2012** | **2013** | **2014** | **2015** | **2015 vs 2014**  **Growth (%)** |
| Total retail sales of consumer goods | 1899.92 | 2266.27 | 2601.20 | 2936.85 | 3283.02 | 11.8 |
| By location of business unit |  |  |  |  |  |  |
| Towns | 1561.07 | 1863.59 | 2148.26 | 2425.25 | 2691.66 | 11.8 |
| Rural | 338.85 | 402.68 | 452.94 | 511.60 | 591.36 | 11.7 |
| By type of consumption |  |  |  |  |  |  |
| Food and beverage income | 205.59 | 233.14 | 245.96 | 266.13 | 300.33 | 12.8 |
| Merchandise retail | 1694.33 | 2033.13 | 2355.24 | 2670.72 | 2982.69 | 11.7 |
| # Retail sales of commodities by units above designated size | 716.24 | 925.83 | 1160.29 | 1461.75 | 1736.76 | 10.0 |
| # Cereals, oils and foodstuffs | 26.98 | 34.47 | 50.38 | 78.16 | 103.77 | 19.4 |
| Alcohol and tobacco | 40.40 | 72.65 | 75.50 | 90.32 | 115.12 | 13.0 |
| Clothing, shoes and hats, knitted textiles | 46.38 | 50.19 | 58.66 | 68.07 | 75.87 | 5.0 |
| Cosmetics | 5.84 | 6.44 | 10.12 | 13.42 | 14.32 | 2.9 |
| Gold, silver and jewelry | 6.25 | 7.34 | 9.51 | 9.60 | 10.40 | -2.2 |
| Daily necessities | 13.28 | 13.75 | 17.40 | 25.83 | 31.48 | 4.9 |
| Sports and entertainment | 1.73 | 1.73 | 2.67 | 2.99 | 3.12 | -2.3 |
| Books, newspapers and magazines | 7.98 | 8.93 | 9.36 | 11.77 | 13.68 | 6.7 |
| Household appliances and audio equipment | 31.13 | 36.13 | 50.34 | 57.53 | 61.03 | 4.0 |
| Chinese and Western medicines | 10.96 | 24.59 | 41.15 | 55.23 | 73.63 | 16.1 |
| Cultural office supplies | 6.92 | 7.23 | 7.98 | 10.12 | 11.63 | 13.7 |
| Furniture | 0.24 | 0.46 | 0.49 | 2.13 | 3.32 | 2.8 |
| Communication equipment | 4.83 | 3.98 | 4.47 | 4.45 | 7.32 | 22.1 |
| Petroleum and its products | 280.13 | 367.10 | 430.30 | 503.31 | 538.90 | 4.1 |
| Construction and decoration materials | 1.64 | 0.80 | 2.06 | 3.20 | 6.25 | 19.5 |
| Cars | 201.35 | 267.75 | 346.02 | 452.85 | 562.87 | 12.6 |

Among the retail sales of commodities above designated size, communication equipment, construction and decoration materials, grain and oil, food, Chinese and Western medicines, tobacco and alcohol, and automobiles increased by 22.1%, 19.5%, 19.4%, 16.1%, 13.0% and 12.6% respectively over the previous year. Enterprises above designated size sold 47.674 billion yuan of commodities through public networks, an increase of 94.4% over the previous year.

Consumer prices for the whole year rose by 1.8% over the previous year. By category, food rose by 2.6%, tobacco and alcohol by 3.2%, clothing by 0.8%, household equipment and services by 1.3%, medical care and personal goods by 0.7%, transportation and communications by 0.6%, entertainment, education, cultural goods and services by 3.4%, and housing by 0.6%. The ex-factory price of industrial producers dropped by 3.9% and the purchasing price of industrial producers dropped by 2.5% over the previous year.

| Table 9 Price Index of the Province from 2011 to 2015 | | | | | |
| --- | --- | --- | --- | --- | --- |
|  |  |  |  | Previous year = 100 | |
| **Indicator name** | **2011** | **2012** | **2013** | **2014** | **2015** |
| Consumer Price Index | 105.1 | 102.7 | 102.5 | 102.4 | 101.8 |
| Food | 113.5 | 104.7 | 104.1 | 104.2 | 102.6 |
| # Food | 116.8 | 104.5 | 103.3 | 102.6 | 102.6 |
| Meat and poultry and their products | 124.5 | 101.3 | 103.8 | 101.3 | 106.6 |
| Aquatic products | 111.1 | 107.6 | 103.1 | 103.0 | 101.7 |
| Eggs | 114.7 | 95.7 | 104.7 | 104.6 | 99.6 |
| Fresh vegetables | 108.3 | 110.8 | 101.3 | 104.5 | 101.5 |
| Dried and fresh melon and fruit | 112.0 | 100.0 | 104.4 | 116.2 | 96.6 |
| Tobacco and alcohol | 102.4 | 102.8 | 101.5 | 99.8 | 103.2 |
| Clothes | 100.3 | 103.8 | 102.3 | 102.1 | 100.8 |
| Household equipment, supplies and services | 100.2 | 101.1 | 101.1 | 100.7 | 101.3 |
| Medical care and personal products | 102.6 | 102.5 | 101.5 | 101.6 | 100.7 |
| Transportation and communications | 100.1 | 99.6 | 99.6 | 100.2 | 100.6 |
| Entertainment, education, cultural goods and services | 99.9 | 101.2 | 101.8 | 102.4 | 103.4 |
| Live | 102.8 | 101.4 | 103.0 | 101.8 | 100.6 |
| Producer Price Index | 105.4 | 101.0 | 97.4 | 98.3 | 96.1 |
| Purchasing price index of industrial producers | 115.0 | 102.3 | 96.4 | 98.6 | 97.5 |
| Price index of investment in fixed assets | 105.4 | 101.5 | 100.9 | 101.1 | 98.4 |
| Price index of construction and installation works | 107.5 | 102.0 | 101.5 | 101.3 | 98.1 |

## VI. Foreign Economy

The total import and export volume of the year was 76.122 billion yuan, an increase of 14.9% over the previous year. The total import volume was 14.266 billion yuan, an increase of 69.0% over the previous year, of which the import of general trade was 4.885 billion yuan, a decrease of 27.5%, and the import of processing trade was 3.033 billion yuan, an increase of 187.1%. The total export volume was 61.856 billion yuan, an increase of 7.0% over the previous year, of which the export of general trade was 54.258 billion yuan, a decrease of 1.4%. The export of processing trade was 4.140 billion yuan, an increase of 143.4%.

In the whole year, the actual capital in place outside the province was 721.351 billion yuan, an increase of 20.1% over the previous year. 4322 projects were introduced from outside the province, an increase of 84.6% over the previous year. The 11912 of investment in the imported projects agreed upon in the contracts was 1.44 billion yuan, an increase of 58.4% over the previous year. 187 foreign investment projects were newly approved, up 8.7% over the previous year.

## VII. Transportation, Posts and Telecommunications, and Tourism

At the end of the year, the mileage of highways open to traffic in the whole province was 183812 kilometers, an increase of 2.6% over the end of the previous year, of which the mileage of expressways open to traffic was 5128 kilometers, and the number of expressways out of the province increased to 15. The railway mileage is 3037 kilometers, of which the high-speed railway mileage is 701 kilometers, and the number of railway corridors out of the province has increased to 12. Fifty-six new encrypted routes have been started, and the number of navigable cities of Guiyang Airport has increased to 81. The length of inland waterways is 3661 kilometers, and the length of high-grade waterways is 690 kilometers.

The freight turnover volume of railway, highway and waterway in the whole year was 45.825 billion ton kilometers, 89.710 billion ton kilometers and 3.715 billion ton kilometers respectively, accounting for 32.9%, 64.4% and 2.7% of the freight turnover volume of the whole province respectively. The passenger turnover of railway, highway and waterway was 20.754 billion person-kilometers, 44.042 billion person-kilometers and 552 million person-kilometers respectively, accounting for 31.8%, 67.4% and 0.8% of the total passenger turnover of the province. Civil aviation handled 89,600 tons of cargo and mail, up by 7.9% over the previous year; Civil aviation handled 15.6328 million passengers, an increase of 10.0% over the previous year.

| Table 10 Freight and Passenger Transport Volume of the Province from 2011 to 2015 | | | | | | |
| --- | --- | --- | --- | --- | --- | --- |
| **Indicator name** | **2011** | **2012** | **2013** | **2014** | **2015** | **Increase from 2014 to 2015 (%)** |
|
| Cargo turnover (100 million ton-kilometers) | 1060.69 | 1177.78 | 1292.11 | 1442.24 | 1392.51 | 5.0 |
| Railway | 696.36 | 693.68 | 655.85 | 634.35 | 458.25 | -11.6 |
| Highway | 350.10 | 467.60 | 610.64 | 776.95 | 897.10 | 15.5 |
| Water transport | 14.23 | 16.50 | 25.62 | 30.94 | 37.15 | 20.1 |
| Cargo and mail throughput of civil aviation (10,000 tons) | 6.93 | 7.97 | 7.76 | 8.31 | 8.96 | 7.9 |
| Passenger turnover (100 million person-km) | 631.74 | 718.22 | 593.62 | 635.50 | 653.48 | 8.1 |
| Railway | 204.58 | 199.20 | 211.21 | 217.39 | 207.54 | 11.2 |
| Highway | 422.01 | 513.07 | 377.87 | 412.92 | 440.42 | 6.7 |
| Water transport | 5.15 | 5.95 | 4.54 | 5.19 | 5.52 | 6.4 |
| Civil aviation passenger throughput (10,000 person-times) | 747.02 | 890.99 | 1125.46 | 1420.68 | 1563.28 | 10.0 |

In 2001, the business volume of posts and telecommunications was 51.487 billion yuan, up by 34.9% over the previous year. Of this, the volume of telecommunications business was 48.110 billion yuan, up 35.9%, and the volume of postal business was 3.377 billion yuan, up 21.7%. The total volume of express business was 70.3425 million pieces, an increase of 50.7% over the previous year; the revenue of express business was 1.324 billion yuan, an increase of 34.9% over the previous year. At the end of the year, there were 34.8485 million telephone users in the whole province, including 31.7231 million mobile phone users, 101 telephone penetration rate/100 people; there were 3.913 million fixed Internet users, the length of optical cable lines exceeded 600,000 km, and 9.167 million Internet broadband access ports. There are 163,000 mobile communication base stations.

The total number of tourists in the whole year was 376 million, an increase of 17.1% over the previous year. Among them, 375 million domestic tourists were received, an increase of 17.1%; The number of inbound tourists was 940900, an increase of 10.0%. The total tourism revenue reached 351.282 billion yuan, an increase of 21.3% over the previous year.

| Table 11 Total number and total income of tourism industry in the province from 2011 to 2015 | | | | | |
| --- | --- | --- | --- | --- | --- |
| **Indicator name** | **2011** | **2012** | **2013** | **2014** | **2015** |
| Total number of tourists (10,000 person-times) | 17019.36 | 21401.18 | 26761.28 | 32134.94 | 37630.01 |
| Total number of tourists increased over the previous year (%) | 31.8 | 25.7 | 25.0 | 20.1 | 17.1 |
| Total tourism revenue (100 million yuan) | 1429.48 | 1860.16 | 2370.65 | 2895.98 | 3512.82 |
| Total tourism revenue increased over the previous year (%) | 34.7 | 30.1 | 27.4 | 22.2 | 21.3 |

## VIII. Finance and Banking

The total fiscal revenue of the year was 229.425 billion yuan, an increase of 7.7% over the previous year. The general public budget revenue was 150.335 billion yuan, an increase of 10.0% over the previous year, of which tax revenue was 112.596 billion yuan, an increase of 9.7%.

| Table 12 Main Fiscal Revenue of the Province from 2011 to 2015 | | | | | | |
| --- | --- | --- | --- | --- | --- | --- |
|  |  |  |  |  |  | Unit: 100 million yuan |
| **Indicator name** | **2011** | **2012** | **2013** | **2014** | **2015** | **Increase from 2014 to 2015 (%)** |
| Total fiscal revenue | 1329.99 | 1644.48 | 1918.23 | 2130.90 | 2294.25 | 7.7 |
| # General public budget revenue | 773.08 | 1014.05 | 1206.41 | 1366.67 | 1503.35 | 10.0 |
| # Domestic VAT (including VAT) | 76.41 | 85.74 | 96.06 | 117.03 | 120.44 | 2.9 |
| Business tax | 181.73 | 241.53 | 300.78 | 344.49 | 353.08 | 2.5 |
| Corporate income tax | 70.69 | 86.53 | 103.15 | 123.84 | 127.31 | 2.8 |
| Personal income tax | 33.24 | 32.29 | 34.65 | 32.52 | 33.27 | 2.3 |
| Urban maintenance and construction tax | 37.82 | 45.79 | 53.71 | 57.64 | 59.80 | 3.7 |
| Deed tax | 23.91 | 33.00 | 41.42 | 66.93 | 73.40 | 9.7 |
| Income from state-owned capital operation | 6.38 | 7.21 | 10.97 | 12.91 | 15.80 | 22.4 |

The annual general public budget expenditure was 393 billion 21 million yuan, an increase of 10.9% over the previous year. Of this total, 77.062 billion yuan was spent on education, up 21.0%; 33.957 billion yuan on social security and employment, up 13.3%; and 35.750 billion yuan on medical and health care and family planning, up 17.9%. Expenditure on agriculture, forestry and water was 53.461 billion yuan, an increase of 19.5%.

| Table 13 Main Financial Expenditures of the Province from 2011 to 2015 | | | | | | |
| --- | --- | --- | --- | --- | --- | --- |
|  |  |  |  |  |  | Unit: 100 million yuan |
| **Indicator name** | **2011** | **2012** | **2013** | **2014** | **2015** | **Increase from 2014 to 2015 (%)** |
| General public budget expenditure | 2249.40 | 2755.68 | 3082.66 | 3542.80 | 3930.21 | 10.9 |
| # Expenditure on general public services | 307.21 | 430.16 | 488.78 | 422.49 | 432.94 | 2.5 |
| Expenditure on education | 376.86 | 500.51 | 560.67 | 637.03 | 770.62 | 21.0 |
| Expenditure on science and technology | 21.68 | 28.98 | 34.27 | 44.34 | 58.19 | 31.2 |
| Expenditure on culture, sports and media | 35.31 | 49.85 | 48.68 | 54.69 | 61.37 | 12.2 |
| Expenditure on social security and employment | 194.78 | 235.40 | 264.52 | 299.72 | 339.57 | 13.3 |
| Expenditure on health care and family planning | 173.26 | 201.05 | 228.71 | 303.25 | 357.50 | 17.9 |
| Expenditure on energy conservation and environmental protection | 55.45 | 65.73 | 66.44 | 85.34 | 95.87 | 12.3 |
| Expenditure on agriculture, forestry and water | 278.47 | 361.87 | 400.31 | 447.19 | 534.61 | 19.5 |
| Transportation expenditure | 305.16 | 288.56 | 299.79 | 432.01 | 392.56 | -9.1 |

At the end of the year, the balance of various kinds of RMB deposits in the financial institutions of the whole province was 19438 1.64 billion yuan, an increase of 26.9% over the end of the previous year. Among them, household deposits were 739.486 billion yuan, accounting for 38.0% of the RMB deposit balance of financial institutions; non-financial enterprise deposits were 679.574 billion yuan, accounting for 35.0%; Broad government deposits amounted to 461 billion 466 million yuan, accounting for 23.7%.

At the end of the year, the balance of RMB loans of financial institutions in the whole province was 1505.194 billion yuan, an increase of 21.7% over the end of last year. Among them, household loans amounted to 442.917 billion yuan, accounting for 29.4% of the balance of RMB loans of financial institutions. Loans from non-financial enterprises and institutions amounted to 106.2093 billion yuan, accounting for 70.6%.

| Table 14 Balance of RMB deposits of financial institutions in the province at the end of 2015 | | |
| --- | --- | --- |
|  |  | Unit: 100 million yuan |
| **Indicator name** | **Absolute number** | **Increase over the beginning of the year** |
| Balance of RMB deposits of financial institutions | 19438.64 | 4128.20 |
| Domestic deposits | 19433.26 | 4128.16 |
| Household deposits | 7394.86 | 631.74 |
| Demand deposit | 3877.60 | 281.12 |
| Time and other deposits | 3517.27 | 350.62 |
| Deposits of non-financial enterprises | 6795.74 | 1670.15 |
| Demand deposit | 4489.54 | 1470.95 |
| Time and other deposits | 2306.20 | 199.20 |
| Government deposits in broad sense | 4614.66 | 1336.28 |
| Fiscal deposits | 761.66 | 227.12 |
| Deposits of organs and organizations | 3853.00 | 1109.16 |
| Deposits of non-banking financial institutions | 627.99 | 489.99 |
| Offshore deposits | 5.38 | 0.04 |

| Table 15 Balance of RMB loans of financial institutions in the province at the end of 2015 | | |
| --- | --- | --- |
|  |  | Unit: 100 million yuan |
| **Indicator name** | **Absolute number** | **Increase over the beginning of the year** |
| Balance of RMB loans of financial institutions | 15051.94 | 2683.42 |
| Domestic loans | 15051.32 | 2683.46 |
| Household loans | 4429.17 | 656.10 |
| Short-term loan | 1073.89 | 291.66 |
| Consumer loans | 285.32 | 94.39 |
| Operating loans | 788.58 | 197.27 |
| Medium and long-term loans | 3355.28 | 364.43 |
| Consumer loans | 2228.82 | 286.00 |
| Operating loans | 1126.46 | 78.44 |
| Loans to non-financial enterprises and institutions | 10620.93 | 2026.37 |
| Short-term loan | 2069.44 | 47.78 |
| Medium and long-term loans | 8324.97 | 1912.02 |
| Bill financing | 196.28 | 61.83 |
| Various advances | 30.23 | 4.94 |
| Loans from non-banking financial institutions | 1.22 | 1.00 |
| Offshore loans | 0.62 | -0.05 |

The original insurance premium income of the whole year was 25.780 billion yuan, an increase of 21.0% over the previous year. Among them, property insurance income was 13.395 billion yuan, an increase of 19.1%; life insurance income was 12.385 billion yuan, an increase of 23.1%. Insurance compensation expenditure was 10.697 billion yuan, an increase of 19.3% over the previous year. Among them, property insurance compensation expenditure was 6 billion 664 million yuan, an increase of 19.1%; Life insurance compensation expenditure was 4.033 billion yuan, an increase of 19.5%.

| Table 16 Development of Insurance Industry in the Province in 2015 | | |
| --- | --- | --- |
| Unit: 100 million yuan | | |
| **Indicator name** | **Absolute number** | **Year-on-year growth (%)** |
| Original premium income | 257.80 | 21.0 |
| # Property insurance | 133.95 | 19.1 |
| # Motor Vehicle Insurance | 110.35 | 18.7 |
| Personal insurance | 123.85 | 23.1 |
| Accident Insurance | 9.90 | 15.6 |
| Health insurance | 16.87 | 28.1 |
| Life Insurance | 97.08 | 23.1 |
| Compensation expenses | 106.97 | 19.3 |
| # Property insurance | 66.64 | 19.1 |
| # Motor Vehicle Insurance | 56.47 | 15.0 |
| Personal insurance | 40.33 | 19.5 |
| Accident Insurance | 3.26 | 0.6 |
| Health insurance | 8.18 | 21.5 |
| Life Insurance | 28.89 | 21.6 |

In 2001, funds raised from the securities market totaled 43.733 billion yuan, up by 12.1% over the previous year. At the end of the year, there were 20 listed companies in the province, down 4.8% from the end of the previous year, with a total capital stock of 17.637 billion yuan, up 39.4% from the end of the previous year. The total market value was 527.930 billion yuan, an increase of 31.9% over the end of last year. Securities and futures investors opened 887 thousand and 300 accounts, an increase of 33.67% over the previous year. The 23592 from securities and futures transactions was 0.39 billion yuan, an increase of 97.1% over the previous year.

## Ix. People's livelihood

The annual per capita disposable income of residents in the province was 13696. 61 yuan, a nominal increase of 10.7% over the previous year. By permanent residence, the per capita disposable income of urban residents and rural residents was 24579 64 yuan and 7386.87 yuan, respectively, an increase of 9.0% and 10.7% over the previous year.

| Table 17 Per capita disposable income of urban and rural residents in 2015 | | | |
| --- | --- | --- | --- |
| Unit: yuan | | | |
| **Indicator name** | **Absolute number** | **Share of disposable income (%)** | **Year-on-year growth (%)** |
| Per capita disposable income of urban residents | 24579.64 |  | 9.0 |
| Wage income | 14166.15 | 57.6 | 7.8 |
| Net income from operations | 3729.81 | 15.2 | 17.6 |
| Net income from property | 1868.13 | 7.6 | 7.0 |
| Net income transferred | 4815.55 | 19.6 | 7.5 |
| Per capita disposable income of rural residents | 7386.87 |  | 10.7 |
| Wage income | 2897.14 | 39.2 | 14.9 |
| Net income from operations | 2878.71 | 39.0 | 8.9 |
| Net income from property | 83.7 | 1.1 | 17.9 |
| Net income transferred | 1527.32 | 20.7 | 6.4 |

The annual per capita consumption expenditure of permanent urban residents was 16914. 20 yuan, an increase of 10.9% over the previous year. The per capita consumption expenditure of rural residents was 6644.93 yuan, an increase of 11.3% over the previous year.

| Table 18 Per capita consumption expenditure of urban and rural residents in 2015 | | | |
| --- | --- | --- | --- |
| Unit: yuan | | | |
| **Indicator name** | **Absolute number** | **As a percentage of consumer spending**  **Proportion of (%)** | **Year-on-year growth (%)** |
| Per capita consumption expenditure of permanent urban residents | 16914.20 |  | 10.9 |
| Food, tobacco and alcohol | 5757.29 | 34.0 | 8.2 |
| Clothes | 1346.73 | 8.0 | 8.1 |
| Live | 2993.81 | 17.7 | 23.1 |
| Daily necessities and services | 1078.55 | 6.4 | -1.1 |
| Health care | 872.24 | 5.2 | -5.4 |
| Traffic and communication | 2248.35 | 13.3 | 20.0 |
| Education, culture and entertainment | 2312.69 | 13.7 | 11.7 |
| Other supplies and services | 304.53 | 1.8 | 3.5 |
| Per capita consumption expenditure of rural residents | 6644.93 |  | 11.3 |
| Food, tobacco and alcohol | 2644.56 | 39.8 | 6.3 |
| Clothes | 355.36 | 5.3 | 4.0 |
| Live | 1355.57 | 20.4 | 12.8 |
| Daily necessities and services | 379.8 | 5.7 | 7.0 |
| Health care | 449.46 | 6.8 | 20.5 |
| Traffic and communication | 784.23 | 11.8 | 23.2 |
| Education, culture and entertainment | 584.75 | 8.8 | 21.5 |
| Other supplies and services | 91.2 | 1.4 | -1.0 |

| Table 19 Number of durable consumer goods owned per 100 urban households in the province in 2015 | | |
| --- | --- | --- |
| **Indicator name** | **Absolute number** | **Year-on-year growth (%)** |
| Water heater (set) | 82.3 | 7.4 |
| Air conditioner (set) | 27.81 | 35.8 |
| Camera (part) | 4.62 | 8.8 |
| Computer (set) | 64.44 | 1.4 |
| Mobile phone (unit) | 236.52 | 3.7 |
| Color TV set | 106.23 | 1.1 |
| Refrigerator (cabinet) | 93.94 | 3.6 |
| Motorcycle (unit) | 17.91 | -14.0 |
| Family car (unit) | 23.74 | 20.7 |

| Table 20 Number of durable consumer goods owned by every 100 permanent rural households in the province in 2015 | | |
| --- | --- | --- |
| **Indicator name** | **Absolute number** | **Year-on-year growth (%)** |
| Washing machine (set) | 77.8 | 3.5 |
| Motorcycle (unit) | 54.9 | 13.4 |
| Color TV set | 101.8 | 2.9 |
| Water heater (set) | 21.6 | 10.2 |
| Fixed telephone (unit) | 8.4 | -36.8 |
| Mobile phone (unit) | 227.3 | 4.2 |
| Refrigerator (cabinet) | 62.4 | 6.4 |

## X. Education, Science and Technology

At the end of the year, there were 26 thousand and 600 schools at all levels in the province, down 3.7% from the end of last year. There were 10.8107 million students in schools at all levels, an increase of 1.5% over the end of the previous year, and 487,200 full-time teachers in schools at all levels, an increase of 5.3% over the end of the previous year. The enrollment rate of primary school-age children was 99.5%, an increase of 0.4 percentage points over the previous year; the gross enrollment rate of junior middle school students was 104.0%, an increase of 1.5 percentage points over the previous year; and the gross enrollment rate of senior middle school students was 86.1%, an increase of 8.1 percentage points over the previous year. The gross enrollment rate of higher education was 31.2%, an increase of 1.8 percentage points over the previous year.

| Table 21 Number of Enrollment, Students and Graduates in the Province in 2015 | | | | | | |
| --- | --- | --- | --- | --- | --- | --- |
| Unit: 10,000 persons | | | | | | |
| **Indicator name** | **Number of students enrolled** | | **Number of students in school** | | **Number of graduates** | |
|  | **Absolute number** | **Compared with the previous year**  **Growth (%)** | **Absolute number** | **Compared with the previous year**  **Growth (%)** | **Absolute number** | **Compared with the previous year**  **Growth (%)** |
| Graduate Education | 0.54 | 6.1 | 1.55 | 5.6 | 0.45 | 3.2 |
| General Higher Education | 16.02 | 9.8 | 50.09 | 8.8 | 11.68 | 17.3 |
| Higher Vocational Colleges | 8.44 | 15.9 | 19.87 | 15.6 | 4.71 | 17.9 |
| Secondary Vocational Education  (School) | 22.81 | -3.3 | 60.25 | 10.7 | 11.88 | 9.5 |
| Ordinary high school | 34.35 | -1.6 | 97.89 | 3.8 | 28.07 | 17.7 |
| Junior high school | 63.27 | -5.2 | 197.97 | -4.3 | 70.24 | 4.2 |
| Ordinary primary school | 57.24 | 3.2 | 346.31 | 0.0 | 63.69 | -4.2 |

In the whole year, 115 scientific and technological achievements at or above the provincial and ministerial levels were registered, down 21.8% from the previous year. Among them, there were 25 basic theoretical achievements, an increase of 13.6%; 89 technical achievements were applied, down by 28.2%. 654 technology contracts were signed, down 0.6% from the previous year, with a turnover of 260 million yuan, an increase of 29.8%. There were 18295 patent applications, down 18.6% from the previous year. The number of authorized patent 14115 increased by 39.7% over the previous year. 545 enterprises completed product certification, an increase of 21.1% over the previous year.

| Table 22 Main Situation of Science and Technology Development in the Province in 2015 | | |
| --- | --- | --- |
| **Indicator name** | **Absolute number** | **Year-on-year growth (%)** |
| Registration of scientific and technological achievements at or above the provincial and ministerial levels (items) | 115 | -21.8 |
| # Achievements in basic theory | 25 | 13.6 |
| Application of technical achievements | 89 | -28.2 |
| Soft science achievements | 1 | Flat |
| Sign technical contract (item) | 654 | -0.6 |
| Transaction amount (ten thousand yuan) | 260190 | 29.8 |
| Professional and technical personnel of public economic enterprises and institutions (10,000 persons) | 66.6 | 3.7 |
| # Personnel with intermediate and above professional and technical titles | 29.47 | 3.7 |
| Ethnic minorities | 26.04 | 3.7 |
| Female | 30.73 | 3.7 |
| Number of intermediate and above skilled personnel in the province (10,000 persons) | 33.92 | 5.0 |
| Patent application (piece) | 18295 | -18.6 |
| Authorized patent (piece) | 14115 | 39.7 |
| Product quality supervision organization (unit) | 48 | -14.3 |
| # National Product Quality Supervision and Inspection Center | 4 | 100 |
| Product quality and system certification organization (unit) | 1 | Flat |
| Enterprises that have completed product certification (units) | 545 | 21.1 |
| Legal metrological technical institutions (unit) | 113 | Flat |
| Compulsory verification of measuring instruments (10,000 sets) | 41.81 | 39.5 |

## Xi. Culture, Health and Sports

At the end of the year, there were 41 art performance groups, 98 mass art galleries and cultural centers, 96 public libraries, 107 archives, 74 museums and memorials, 6 art performance venues and 1565 comprehensive cultural stations in towns and townships. The number of books published in the whole year is 10361, and the number of magazines published is 17.5098 million copies, and the number of newspapers published is 322 million copies. At the end of the year, the comprehensive population coverage rate of radio was 92.3%, and that of television was 96.0%.

At the end of the year, there were 28,700 health institutions in the province. Among them, there were 0.26 million hospitals and health centers, an increase of 4.0% over the end of last year; There were 102 maternal and child health care centers (institutes and stations), an increase of 2.0%. The number of beds in health institutions was 197 thousand and 100, an increase of 8.5% over the end of last year. There were 186900 health technicians, an increase of 10.1% over the end of last year. Among them, there were 63,400 practicing (assistant) doctors, an increase of 9.7%, and 76,000 registered nurses, an increase of 13.3%. There were 32.9233 million farmers participating in the new rural cooperative medical system, with a participation rate of 99.12%, an increase of 0.22 percentage points over the previous year.

Athletes won 41 awards in major international and domestic sports competitions throughout the year, an increase of 17.1% over the previous year. At the end of the year, there were 59 stadiums in the province.

## XII. Ecology, Environment and Safety in Production

The afforestation area in the whole year was 280000 hectares, and the forest coverage rate at the end of the year was 50.0%, an increase of 1.0 percentage points over the previous year. The water quality of centralized drinking water sources in the central cities of nine cities (prefectures) reached the standard rate of 100%, the excellent rate of air quality index was higher than 90%, and the number of days with good air quality in cities above county level exceeded 95%. At the end of the year, 628 provincial ecological civilization construction demonstration zones were approved, an increase of 66.1% over the end of last year. There are 123 nature reserves, including 9 national nature reserves; the area of nature reserves accounts for 5.6% of the total land area of the province.

The annual investment in environmental protection was 9.574 billion yuan, an increase of 5.0% over the previous year. The treatment capacity of sewage treatment plants in county towns and above was 2.4658 million cubic meters per day, an increase of 28.8% over the previous year, and the sewage treatment rate reached 89.3%, an increase of 2.0 percentage points over the previous year. The 33060 of green area in urban built-up areas was 0.77 hectares, an increase of 12.7% over the previous year, and the green rate of built-up areas was 23.1%, an increase of 1.7 percentage points over the previous year. The harmless treatment rate of municipal solid waste was 82.8%, the comprehensive utilization rate of industrial solid waste was 58.0%, and the industrial reuse rate was 95.0%, which increased by 3.9, 1.1 and 0.8 percentage points respectively over the previous year. The energy consumption of 10000 yuan GDP decreased by 7.46% compared with the previous year.

A total of 1,142 production safety accidents occurred in the whole year, a decrease of 10.2% over the previous year, and 877 people died, a decrease of 10.6%. Among them, 1035 road traffic accidents occurred, down 9.6%; There were 741 deaths, down 6.8%.

## XIII. Population, employment and social security

At the end of the year, the permanent population of the province was 35.295 million. By urban and rural areas, the urban population is 14.8274 million, and the rural population is 20.4676 million. The urban population accounted for 42.01% of the permanent population at the end of the year, an increase of 2 percentage points over the previous year. By gender, the male population is 18.2063 million and the female population is 17.0887 million. The birth rate of the whole province was 13.00 per thousand, an increase of 0.02 per thousand over the previous year, and the death rate was 7.20 per thousand, an increase of 0.02 per thousand over the previous year. The natural population growth rate was 5.80 per thousand, the same as the previous year.

726800 new jobs were created in cities and towns throughout the year, an increase of 6.3% over the previous year. Among them, 145 thousand and 500 unemployed people were re-employed, down 4.8% from the previous year. At the end of the year, the number of registered unemployed people in cities and towns was 144900, and the registered unemployment rate in cities and towns was 3.29%.

At the end of the year, 3.9209 million people in the province participated in the basic old-age insurance for urban employees, an increase of 8.5% over the end of the previous year, of which 2.8801 million were enterprise employees, an increase of 7.3%; 16.4903 million people participated in the basic old-age insurance for urban and rural residents, an increase of 3.9% over the end of the previous year; The number of people participating in unemployment insurance was 2.0531 million, an increase of 7.0% over the end of the previous year; the number of people participating in industrial injury insurance was 2.9028 million, an increase of 5.4% over the end of the previous year; The number of people participating in maternity insurance was 2.6367 million, an increase of 6.0% over the end of last year. We implemented 502,200 government-subsidized housing units in cities and towns, completed 225600 government-subsidized housing units in cities and towns, and renovated 350000 dilapidated houses in rural areas.

At the end of the year, a total of 402800 people in the province enjoyed the minimum living security for urban residents, down 15.4% from the end of the previous year. 3.3148 million people enjoyed the minimum living security for rural residents, down 20.5% from the end of last year. All kinds of social service institutions providing accommodation adopted and assisted 27800 people, of which 23600 were adopted and assisted by old-age service institutions. The province sold 2.498 billion yuan of social welfare lottery tickets, an increase of 16.1% over the previous year. It raised 78100 yuan of social welfare funds, an increase of 12.1%.

Note:

[1] Data for 2015 are preliminary statistics, and data for 2014 and previous years are annual reports. Due to rounding, some data are not equal to the total of sub-items. "#" indicates the item.

[2] The absolute figures of GDP, industrial added value and per capita GDP are calculated at current prices, and the growth rate is calculated at comparable prices.

[3] The statistical caliber of industries above designated size is industrial enterprises with annual main business income of 20 million yuan or more. The statistical caliber of fixed assets investment is the investment in fixed assets projects and real estate development projects with a total planned investment of 5 million yuan or more. The statistical caliber of the wholesale industry above the designated size is the wholesale enterprises and individual households with annual main business income of 20 million yuan or more, the statistical caliber of the retail industry above the designated size is the retail enterprises and individual households with annual main business income of 5 million yuan or more, and the statistical caliber of the accommodation and catering industry above the designated size is the accommodation and catering enterprises and individual households with annual main business income of 2 million yuan or more.

[4] According to the provisions of the national statistical system, the statistical caliber of fixed assets investment projects has been increased from 500,000 yuan to 5 million yuan since 2011. Therefore, the average annual growth rate of fixed assets investment in the same caliber during the 12th Five-Year Plan period can only be calculated from 2012 to 2015.
