# Supplementary material for: Carbon peaking prediction scenarios based on different neural network models: A case study of Guizhou Province
Source: PLoS One. 2024 Jun 25;19(6):e0296596. doi: 10.1371/journal.pone.0296596 (PMC11198898; doi:10.1371/journal.pone.0296596)
Supplement: Attachment — Submitted filename: Response to Reviewers.doc [file pone.0296596.s002.doc]

Dear editor：

Thank you very much for your letter. We have learned much from your and three reviewers’ comments, which are fair, encouraging and constructive. After carefully studying the comments and your advice, we have made corresponding changes. The main revisions are listed below.

# For reviewer one:

1. It is noted that your manuscript needs careful editing by someone with expertise in technical English editing paying particular attention to English grammar, spelling, and sentence structure so that the goals and results of the study are clear to the reader

. We have carefully revised the article and edited it in English in the attachment

2. Suggest reading these literature to expand research ideas

Thank you very much. This is a great recommendation. We have studied these high-quality articles carefully, and have been inspired by their ideas. We have also made targeted modifications to the articles and added them to the references

# For reviewer two:

1 Abstract. The review part should be more concise, and the findings part should be more accurate and detailedly. The summary can be accurately summarized in several points

The abstract has been streamlined and modified
2 Tons should be tonnes

Conducted a full text review and made modifications
3 Introduction: The novelty of this paper should be further justified by highlighting main contributions to the existing introduction and literature review. For example, what are the other researches on the prediction model? There are many articles, e.g., one about the ARIMA method entitled “Ecological footprint simulation and prediction by ARIMA model-A case study in Henan Province of China. <https://doi.org/> 10.1016/j.ecolind.2009.06.007”.

This is a great recommendation. We have studied the article carefully, made modifications and adjustments to this section, and also cited the literature
4 Yuan should be CHY

Conducted a full text review and made modifications
5 The formula part of the method of the full text, as detailed and specific as possible, at present, the formula is few and unclear

Made modifications and adjustments
6 There are still unclear in the graphics

Unclear images removed
7 Grammar problems. Need to check the polish carefully

Made modifications and adjustments
8 There should be a conclusion. The conclusion can also be clearly and concisely divided into several points.
9 The conclusion can be preceded by a discussion section. The section should enhance the relevant discussion. For example, the comparison of the results of this paper with those of its predecessor could be strengthened with regard to the results of carbon emissions in China or World or GUIZHOU. One China’s article entitled “China's CO2 emissions: A systematical decomposition concurrently from multi-sectors and multi-stages since 1980 by an extended logarithmic mean divisia index. <https://doi.org/10.1016/j.esr.2023.101141>”. Next world one entitled “Contribution of Renewable Energy Consumption to CO2 Emission Mitigation: A Comparative Analysis from a Global Geographic Perspective, <https://doi.org/> 10.3390/su13073853”

We have added these two parts and made additions and modifications. Carefully read the provided references. This is constructive feedback, thank you for the reviewer's suggestions.we also cited the article

# For reviewer three:

1  There are instances of colloquial English expressions and language issues. Please review carefully

Made modifications and adjustments
2 The motivation for using ELM and WOA to enhance the neural network must be established. It would be beneficial to elaborate on why these optimization strategies were chosen and whether they have been used in the field.

An explanation was added to the text:It is worth noting that in ELM, the input data is transformed by the hidden layer, and then the output layer produces the result. This process is "forward propagation", that is, information flows from the input layer to the output layer. However, the most important thing in this process is backpropagation, that is, how to adjust network parameters to improve performance when the output does not meet expectations. Back-propagation algorithm is an important optimization technique, which calculates how much the weight of each layer needs to be adjusted according to the difference between the actual output and the expected output of the network, and then optimizes the network. In ELM, due to its single-layer feedforward feature, backpropagation is mainly used to adjust the weights and biases, so that the network can better adapt to the training data. Whale optimization algorithm WOA is used to optimize the learning rate of convolutional neural network CNN and the number of hidden layer neural networks. It has higher optimization efficiency and better optimization results. Especially when the data used is only a single column of time series data, WOA algorithm may show better optimization results when dealing with more complex data. Therefore, the WOA algorithm is expected to become an important tool in the optimization of convolutional neural networks.
3  It is necessary to provide the training curves and convergence of the machine learning models to ensure they do not suffer overfitting on such a limited dataset.

Convergence explanation has been added to the article

Eg:

1.When its value is small, although it can make the simulation results of the model more accurate, it significantly increases the training time. In general studies, the learning rate γ is usually set between 0 and 1. In this paper, through continuous debugging and comparison of the training effect in the training process, the learning rate γ = 0.1 was selected. The accuracy of the network training was required to be 0.001, and the maximum number of training sessions was 500.

2.When setting the initial weights and thresholds of the neural network, a set of randomly generated initial values was selected, because there was no relevant setting principle. The BP neural network can learn the mapping relationship between the input and output automatically, generate initial parameters randomly, and modify the weights and thresholds of the network continuously through error back propagation; however, randomly selected initial weights and thresholds are usually inversely proportional to the convergence speed of neural network training; that is, the larger the value, the slower the convergence speed. In this case, the final training results easily fall into the local optimum, and it is difficult to obtain ideal calculation and prediction results.

At the same time, the data table is given in the paper.

Thank you to the judges for their hard work. During the traditional Chinese Spring Festival, I also sincerely wish everyone a happy holiday

Yang Wu
